# Genomic Insights into ANI-dDDH Relationships in *Nocardiopsis* and the Novel Species *Nocardiopsis camelliae* sp. nov

**DOI:** 10.3390/biology15141119

**Published:** 2026-07-10

**Authors:** Ting Tang, Wenguang Huang, Huiping Zhong, Ping Mo, Yaxi Zheng, Li Fu, Kaiqin Li, Jian Gao

**Affiliations:** 1 Science and Technology Innovation Team for Efficient Agricultural Production and Deep Processing at General University in Hunan Province, College of Life and Environmental Sciences, Hunan University of Arts and Science, Changde 415000, China; tangting202512@163.com (T.T.); wenguanghuang05@163.com (W.H.); 18373576156@163.com (H.Z.); yaxi2005@126.com (Y.Z.); 2 College of Furong, Hunan University of Arts and Science, Changde 415000, China; 3 College of Life Sciences, Wuhan University, Wuhan 430072, China; fuli24@whu.edu.cn; 4 School of Computer Science and Engineering, Hunan University of Science and Technology, Xiangtan 411201, China; 5 School of Life and Health Sciences, Hunan University of Science and Technology, Xiangtan 411201, China; xtgojian@hnust.edu.cn

**Keywords:** ANI, ANIm/ANIb, dDDH, polyphasic taxonomy, *Nocardiopsis camelliae*

## Abstract

Average nucleotide identity (ANI) values of 95–96% and digital DNA–DNA hybridization (dDDH) values of 70% are currently regarded as the gold standards for bacterial species delineation. However, the accuracy and applicability of ANI thresholds may vary among genera and depend on genome quality. In this study, three genome quality standards were applied to re-evaluate ANI classification thresholds for the genus *Nocardiopsis*: >90% completeness and <5% contamination (13 strains, 78 genome pairs), >90% completeness and <10% contamination (34 strains, 561 genome pairs), and >50% completeness and <10% contamination (40 strains, 780 genome pairs). Based on these analyses, we preliminarily estimated the species delineation thresholds for the genus *Nocardiopsis* as 96.68% for ANIm and 96.15% for ANIb, using the currently available genome datasets. Strain HUAS JQ3^T^ was isolated from leaves of *Camellia oleifera* Abel collected in Taoyuan County, northwestern Hunan Province, China. Phylogenetic, genomic, morphological, cultural, and physiological analyses were conducted to determine its taxonomic status. Full-length 16S rRNA gene sequence analysis showed that strain HUAS JQ3^T^ belongs to the genus *Nocardiopsis* and shares the highest sequence similarities with *N. dassonvillei* subsp. *crassaminis* D1^T^ (100.00%), *N. alborubida* NBRC 13392^T^ (99.86%), *N. synnemataformans* DSM 44143^T^ (99.73%), and *N. akebiae* HDS 12^T^ (99.73%). Phylogenetic analysis based on 16S rRNA gene sequences clustered strain HUAS JQ3^T^ with *N. alborubida*, *N. synnemataformans*, and *N. dassonvillei* subsp. *crassaminis*. In contrast, whole-genome phylogenetic analysis indicated that strain HUAS JQ3^T^ is most closely related to *N. akebiae* HDS 12^T^. Nevertheless, the ANIm/ANIb and dDDH values between strain HUAS JQ3^T^ and *N. akebiae* HDS 12^T^ were below the proposed species delineation thresholds of 70% dDDH and 96.68%/96.15% ANIm/b for the genus *Nocardiopsis*. In addition, strain HUAS JQ3^T^ exhibited distinct morphological, cell culture, physiological, and biochemical characteristics compared with *N. akebiae* HDS 12^T^. The combined genotypic and phenotypic evidence gathered in this study demonstrates that strain HUAS JQ3^T^ (= MCCC 1K08696^T^ = JCM 36305^T^) represents a novel species of the genus *Nocardiopsis*, for which the name *Nocardiopsis camelliae* sp. nov. is proposed.

## Simple Summary

Scientists need clear rules to decide when a group of bacteria belongs to a new species. Currently, they often use DNA similarity cut offs—for example, around 95–96% average nucleotide identity or 70% digital DNA–DNA hybridization. However, these values may not work perfectly for all bacterial families. In this study, we focused on the genus *Nocardiopsis*, which includes bacteria found in various environments. By analyzing all available high-quality genomes, we preliminarily estimated that for this genus, the average nucleotide identity (ANI) threshold should be set at 96.68% (using one calculation method) or 96.15% (using another), together with the 70% dDDH standard. We then examined a strain isolated from camellia plant leaves in China. Its DNA similarity to its closest known relative fell below these new thresholds, and it also showed distinct physical and growth features. Based on this combined evidence, we propose it as a new species, named *Nocardiopsis camelliae*. Our work provides more accurate tools for identifying these bacteria, which may help in future discoveries of useful natural products.

## 1. Introduction

Actinomycetes are a diverse group of bacteria known for their ability to produce a wide range of bioactive compounds. These microorganisms have been extensively studied for their potential applications in medicine, agriculture, and biotechnology [1,2,3]. Among the various habitats in which actinomycetes are found, plants are recognized as important reservoirs of these bacteria. Plant-associated endophytic actinomycetes, in particular, have attracted significant attention due to their ability to colonize internal plant tissues without causing harm. Endophytes are microorganisms that inhabit healthy plant organs, including roots, stems, leaves, flowers, and fruits, without inducing disease [4,5]. They occupy specific ecological niches and represent an important component of plant microecosystems [6,7]. Endophytes play critical roles in plant growth, health, and responses to biotic stress [8,9]. In addition, the secondary metabolites produced by plant endophytes are valuable sources of compounds with anticancer, antioxidant, antiviral, bacteriostatic, and insecticidal activities [10]. Endophytic actinomycetes have been isolated from a wide range of plant species [11,12] and have been shown to contribute to plant growth promotion, disease suppression, and stress tolerance [13,14]. These bacteria enhance plant health by producing bioactive compounds that stimulate growth, inhibit pathogens, and improve tolerance to environmental stress. Accordingly, there is increasing interest in exploring the diversity of plant endophytic actinomycetes, particularly in Chinese medicinal plants, to identify novel species with unique bioactive properties and potential applications in agriculture and medicine [15]. Investigating the composition of endophytic communities is therefore important for identifying novel microbial resources and developing plant-associated biotechnological applications.

In the course of screening for novel actinobacteria capable of producing bioactive compounds, we isolated hundreds of actinomycete strains from rhizosphere soil and endophytic tissues of *Camellia oleifera Abel*. Among these, strain HUAS JQ3^T^ showed the highest genomic similarity to *Nocardiopsis akebiae* HDS 12^T^ within the genus *Nocardiopsis*. Notably, the average nucleotide identity (ANI) and digital DNA–DNA hybridization (dDDH) values between strain HUAS JQ3^T^ and *N. akebiae* HDS 12^T^ were >96% and <70%, respectively. Based on the dDDH threshold of 70% for species delineation [16], strain HUAS JQ3^T^ would be considered a novel species. However, based on the ANI threshold of 95–96% [17], it would be classified as an existing species. Therefore, the first objective of this study was to resolve this apparent discrepancy within the genus *Nocardiopsis*. In addition, the taxonomic status of strain HUAS JQ3^T^ was determined using a polyphasic approach, integrating the findings from this analysis.

## 2. Materials and Methods

### 2.1. Understanding the Relationship Between ANI and dDDH Values in the Genus Nocardiopsis

As of March 9, 2026, genome data for 40 type strains of the genus *Nocardiopsis* were available in the NCBI database, including three synonyms and one effectively but not validly published name (excluding unverified source organisms; Supplementary Table S1). Although 53 species have been validly described in this genus, many lack genome data or are of insufficient quality (i.e., <50% completeness and >10% contamination). Therefore, we initially downloaded all 40 accessible genomes to accurately evaluate the ANI–dDDH relationship. Genome quality was assessed using CheckM (v1.2.3) [18] based on completeness and contamination. To examine the impact of genome quality on the derived thresholds, the strains were divided into three groups according to different quality criteria: (1) >90% completeness and <5% contamination (13 strains; Supplementary Table S2); (2) >90% completeness and <10% contamination (34 strains; Supplementary Table S3); and (3) >50% completeness and <10% contamination (40 strains; Supplementary Table S1). Then, they were retained for subsequent analyses [19]. Average nucleotide identity (ANI), including ANI based on MUMmer (ANIm) and BLAST (ANIb), and digital DNA–DNA hybridization (dDDH) values were calculated using the JSpeciesWS web server (https://jspecies.ribohost.com/jspeciesws/#analyse, © 2014–2025. Ribocon GmbH-Version: 5.0.3, accessed from 3 August 2025 to 30 August 2025) [20] and the Genome-to-Genome Distance Calculator 3.0 with local alignment tool (BLAST+, recommended) (http://ggdc.dsmz.de/, accessed from 28 June 2025 to 15 August 2025) [21], respectively. The relationship between ANI and dDDH values was evaluated using the method described by Hu et al. (2022) [22].

To establish species delineation thresholds for the genus *Nocardiopsis*, we analyzed the relationship between ANI (ANIm and ANIb) and dDDH values using nonlinear regression. All curve-fitting analyses were performed using OriginPro 2025 (OriginLab Corporation, Northampton, MA, USA).

For each of the three genome-quality datasets (13, 34, and 40 strains) and for each ANI type (ANIm and ANIb), we independently tested multiple nonlinear models available in OriginPro. The best-fitting model for each curve was selected based on the highest coefficient of determination (R^2^) and lowest reduced Chi-Sqr value.

To derive species delineation thresholds, we used the “Find X from Y” tool in OriginPro. For each fitted curve, we interpolated the ANIm and ANIb values corresponding to the standard species demarcation cutoff of 70% dDDH. The 95% confidence bands for the fitted curves were generated by OriginPro to visualize the uncertainty of the estimates.

### 2.2. Evaluation of the Taxonomic Status of Strain HUAS JQ3^T^

#### 2.2.1. Isolation and Maintenance

Strain HUAS JQ3^T^ was isolated from the leaves of *Camellia oleifera* Abel collected in Taoyuan County, northwestern Hunan Province, South China (28°57′13.57″ N, 111°26′3.38″ E). Leaf samples were processed according to the method described by Deng et al. [23]. Actinobacteria were isolated using modified Gause’s synthetic No. 1 medium supplemented with 1.0 mL phenol (100 g/L) and 1.0 mL nystatin (50 g/L) [24].

Strain HUAS JQ3^T^ was maintained on Gause’s synthetic No. 1 medium [25] at 4 °C and preserved in 30% glycerol at −80 °C. The type strain *N. akebiae* HDS 12^T^ was obtained from our laboratory collection. Strains HUAS JQ3^T^ and *N. akebiae* HDS 12^T^ were cultured under identical conditions for comparative analyses.

#### 2.2.2. Morphological, In Vitro Culture, and Physio-Biochemical Properties

The spore chain morphology, spore surface, and spore shape of strain HUAS JQ3^T^ were examined on Gause’s synthetic No. 1 medium [25] after incubation at 28 °C for 21 days using light microscopy (NE620, Ningbo Yongxin Optics Co., Ltd., Ningbo, China) and scanning electron microscopy (FEI Quanta 450, Hillsboro, Oregon, USA). Morphological characteristics of strains HUAS JQ3^T^ and *N. akebiae* HDS 12^T^ were further assessed on Gause’s synthetic No. 1 medium [25], Reasoner’ 2A [26], ISP media 2–7 following incubation at 28 °C for 21 days, as described by Shirling and Gottlieb [27]. The colors of aerial mycelium, substrate mycelium, and diffusible pigments were determined using the Color Standards and Color Nomenclature [28].

Growth was evaluated at temperatures ranging from 4 to 55 °C (4, 10, 15, 20, 25, 30, 35, 37, 40, 45, 50, and 55 °C) on Gause’s synthetic No. 1 medium for 14 days. Growth was evaluated at pH 4.0–11.0 (in increments of 1.0 pH unit) and in the presence of 0–15.0% NaCl (*w*/*v*, in increments of 1.0%) using ISP 2 medium after incubation at 28 °C for 14 days. Carbon and nitrogen source utilization was determined according to the methods of Shirling and Gottlieb [27]. Milk peptonization, gelatin liquefaction, starch hydrolysis, and Tweens (20, 40, 60, and 80) hydrolysis was assessed following the procedures described by Ruan and Huang [29]. All tests were performed under the same conditions and repeated three times.

#### 2.2.3. Chemotaxonomic Analysis

For chemotaxonomic analysis, HUAS JQ3^T^ cells were obtained from cultures grown in tryptic soy broth in shaking flasks after incubation at 28 °C for 14 days. Cellular fatty acid analysis was performed using the Sherlock MIDI protocol [30] by the Marine Culture Collection of China (MCCC). Diaminopimelic acid and the whole-cell sugar composition were determined using the procedures described by Hasegawa et al. (1983) and Lechevalier and Lechevalier (1970) [31,32], respectively. Menaquinones and phospholipids were extracted and analyzed as described by Ruan and Huang [29].

#### 2.2.4. Phylogenetic Analysis

Genomic DNA was extracted and sequenced using the NovaSeq 6000 platform (Illumina Inc., CA, USA) and the PromethION platform (Oxford Nanopore Technologies, Oxford, UK). Sequencing was performed by Wuhan Benagen Technology Co., Ltd. (Wuhan, China). The genome completeness and contamination of strain HUAS JQ3^T^ were 98.82% and 1.96%, respectively, which meet the criteria (Supplementary Table S1). The complete 16S rRNA gene sequence of strain HUAS JQ3^T^ was obtained from the whole-genome sequence using the NCBI Prokaryotic Genome Annotation Pipeline (PGAP, v4.4) and compared against the EzBioCloud database [33]. The 16S rRNA gene sequences of strain HUAS JQ3^T^ and 20 closely related type strains were then retrieved and used for phylogenetic analysis. Phylogenetic trees were constructed using the neighbor-joining (NJ) [34], maximum-likelihood (ML) [35], and maximum-parsimony (MP) [36] methods in MEGA version 11 [37], with 1000 bootstrap replicates. ANI, including ANIb (BLAST-based) and ANIm (MUMmer-based), and dDDH values between strain HUAS JQ3^T^ and type strains sharing ≥ 98.7% 16S rRNA gene sequence similarity were calculated using the JSpeciesWS web server (https://jspecies.ribohost.com/jspeciesws/#analyse, © 2014–2025. Ribocon GmbH-Version: 5.0.3, accessed from 3 August 2025 to 30 August 2025) [20] and the Genome-to-Genome Distance Calculator 3.0 with local alignment tool (BLAST+, recommended) (http://ggdc.dsmz.de/distcalc2.php, 28 June 2025 to 15 August 2025) [21], respectively. Phylogenomic analysis was performed using the Type (Strain) Genome Server (TYGS; https://tygs.dsmz.de/, accessed from 28 June 2025 to 15 August 2025) [38]. Genome annotation for strains HUAS JQ3^T^ and *N. akebiae* HDS 12^T^ was carried out using the NCBI Prokaryotic Genome Annotation Pipeline (PGAP) and the Rapid Annotation using Subsystem Technology (RAST) server [39]. Secondary metabolite biosynthetic gene clusters of strains HUAS JQ3^T^ and *N. akebiae* HDS 12^T^ were predicted using antiSMASH version 6.0.1 [40]. Antibiotic resistance genes were identified using the Comprehensive Antibiotic Resistance Database (CARD) [41]. CRISPR arrays and genomic islands were predicted using CRISPRCasFinder [42] and IslandViewer 4 [43], respectively.

## 3. Results

### 3.1. Relationship Between ANI and dDDH Values in the Genus Nocardiopsis

To evaluate the robustness of species delineation thresholds in the genus *Nocardiopsis*, we analyzed three datasets with different genome quality criteria: (1) >90% completeness and <5% contamination (13 strains, 78 pairs) (Table S4) [44]; (2) >90% completeness and <10% contamination (34 strains, 561 pairs) (Table S5) [45]; and (3) >50% completeness and <10% contamination (40 strains, 780 pairs) (Table S6). The coefficient of determination (R^2^) for the ANIm-dDDH relationship was 0.99878, 0.9861, and 0.98337 for the three datasets, respectively, while that for the ANIb-dDDH relationship was 0.99878, 0.98321, and 0.98106, respectively.

For the 13-strain high-quality dataset (>90% completeness, <5% contamination), the ExpGrow1 model [y = y0 + A1 × exp ((x − x0)/t1)] was selected for ANIm-dDDH (R^2^ = 0.99882, Adj. R^2^ = 0.99878, n = 78), and the ExpDec1 model [y = y0 + A1 × exp(−x/t1)] was selected for ANIb-dDDH (R^2^ = 0.99882, Adj. R^2^ = 0.99878, n = 78). Using the 70% dDDH threshold as the reference, the predicted ANIm threshold was 96.68%, and the predicted ANIb threshold was 96.15%.

**Figure 1:**
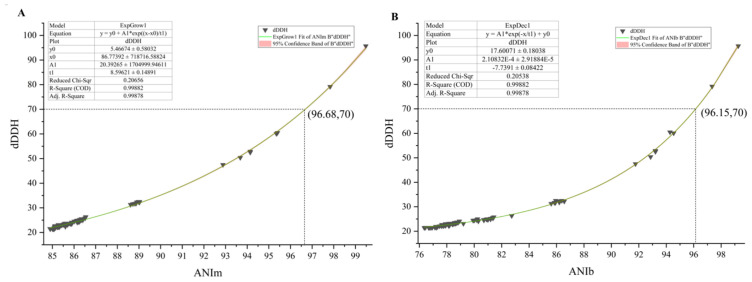
Correlations between ANIm/b and dDDH from 13 *Nocardiopsis* species (78 pairs). Note: (**A**) ANIm-dDDH; (**B**) ANIb-dDDH. The shaded area indicates the 95% confidence band of the fitted regression model. The dashed line denotes the estimated species delineation threshold (ANIm = 96.70%).

**Figure 2:**
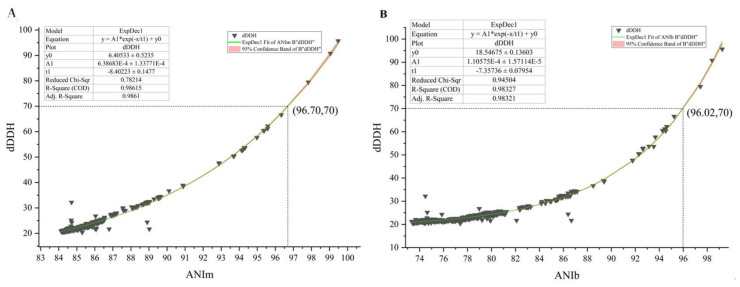
Correlations between ANIm/b and dDDH from 34 *Nocardiopsis* species (561 pairs). Note: (**A**) ANIm-dDDH; (**B**) ANIb-dDDH.

**Figure 3:**
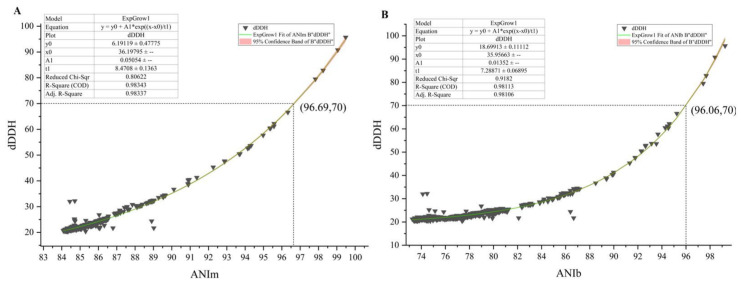
Correlations between ANIm/b and dDDH from 40 *Nocardiopsis* species (780 pairs). Note: (**A**) ANIm-dDDH; (**B**) ANIb-dDDH.

The 95% confidence bands of the fitted curves (shown as shaded areas in Figure 1, Figure 2 and Figure 3) are narrow around the threshold point, and the consistently high R^2^ values (≥0.983) indicate that the data points cluster tightly around the fitted curves. Sensitivity analysis using the three different genome quality filters yielded highly consistent ANIm thresholds of 96.68%, 96.70%, and 96.69%, respectively (Table S7), suggesting that the estimates are not artifacts of a particular dataset. Collectively, these results support the preliminary species delineation thresholds of 96.68% (ANIm) and 96.15% (ANIb) for the genus *Nocardiopsis*, based on currently available genome data.

It should be noted, however, that pairwise ANI and dDDH comparisons within each dataset are not statistically independent, as each genome contributes to multiple pairwise comparisons. While our cross-dataset sensitivity analysis using three independently assembled genome sets with different strain compositions yielded highly consistent thresholds, we acknowledge that this does not fully resolve the non-independence issue. Future studies with a more comprehensive set of *Nocardiopsis* genomes could employ bootstrap resampling at the genome or species level to provide more rigorous uncertainty estimates for genus-specific thresholds.

### 3.2. Evaluation of the Taxonomic Status of Strain HUAS JQ3^T^

#### 3.2.1. Phenotypic Analysis

**Figure 4:**
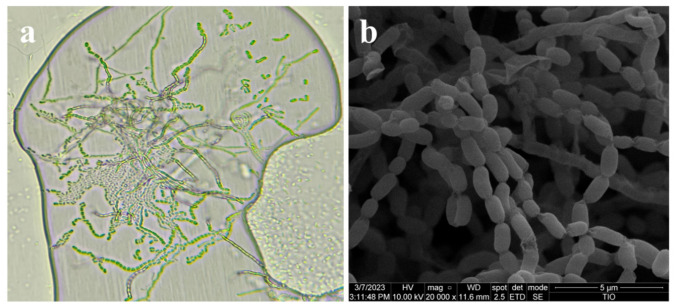
Light microscope image (100 × 40) (**a**) and scanning electron microscope image (20,000×) (**b**) of strain HUAS JQ3^T^ were observed on Gause’s synthetic No. 1 medium after incubating at 28 °C for 21 days.

Phenotypic analysis of strain HUAS JQ3^T^ showed that light drab diffusible pigments were produced on ISP 2 medium, whereas deep olive-buff diffusible pigments were produced on ISP 4 medium. Ivory yellow aerial mycelium and dark greenish-olive substrate mycelium were well produced on Gause’s synthetic No. 1 medium (Supplementary Table S8). In addition, spore chains were slightly flexuous and consisted of rod-shaped spores with smooth surfaces (Figure 4). We also found that strain growth occurs at 15–40 °C and pH 6.0–9.0, with optimal growth at 28 °C and pH 7.0. These cells could tolerate up to 13.0% NaCl (*w*/*v*) on ISP 2, with optimal growth at 0–1.0% (*w*/*v*) NaCl. This strain tested negative for starch hydrolysis and positive for gelatin liquefaction.

Importantly, our data showed that this strain can utilize cellulose, d-fructose, d-ribose, d-xylose, l-arabinose, l-rhamnose, *myo*-inositol, starch, sucrose, and trehalose as sole carbon sources, but not d-galactose, d-mannose, maltose, lactose, and raffinose. Further, strain HUAS JQ3^T^ used the following compounds as sole nitrogen sources: l-cysteine, l-glutamic acid, l-hydroxyproline, l-ornithine, l-phenylalanine, and p-hydroxyphenylalanine. Glycine, histidine, l-asparagine, and l-alanine cannot be utilized as sole nitrogen sources (Table 1).

#### 3.2.2. Chemotaxonomic Characterization

We found that the predominant cellular fatty acids of strain HUAS JQ3^T^ (>10.0%) were *iso*-C_16:0_ (33.6%), C_18:1_ ω9c (17.1%) and C_18:0_ 10-methyl (TBSA) (10.9%) (Supplementary Table S9). Whole-cell sugars were non-diagnostic sugars, with the cell-wall diamino acid being *meso*-diaminopimelic acid (*mes*-DAP).

The menaquinones were MK-10(H_2_) (50.3%), MK-10(H_4_) (34.7%), and MK-10(H_6_) (12.4%), and the polar lipids consisted of diphosphatidylglycerol (DPG), phosphatidylinositol mannosides (PIMs) and unidentified lipids (L1 and L2) (Supplementary Figure S1).

#### 3.2.3. Genomic Analysis

Genome sequencing revealed that strain HUAS JQ3^T^ consisted of 6,813,696 bp, with a DNA G + C content of 72.4%. A total of 5953 genes were predicted, including 5820 coding genes, 78 RNA genes, and 57 pseudogenes. In total, 5875 coding sequences (CDSs) were identified, comprising 5820 protein-coding CDSs and 55 non-protein-coding CDSs (Supplementary Table S10).

Genome annotation revealed 298 subsystems, representing an 18% subsystem coverage. Major functional categories included “Carbohydrates” (294 CDSs), “Amino Acids and Derivatives” (266 CDSs), “Protein Metabolism” (196 CDSs), “Cofactors, Vitamins, Prosthetic Groups, and Pigments” (165 CDSs), “Fatty Acids, Lipids, and Isoprenoids” (94 CDSs), and “Nucleosides and Nucleotides” (91 CDSs) (Supplementary Table S10). Comparative analysis of RAST-based subsystem annotations revealed that strain HUAS JQ3^T^ and *N. akebiae* HDS 12^T^ shared highly similar functional gene profiles across most categories. Notable differences were observed in Carbohydrates (294 vs. 301), Amino Acids and Derivatives (266 vs. 275), Fatty Acids and Lipids (94 vs. 101), DNA Metabolism (85 vs. 72), and Virulence (48 vs. 39), while other categories remained largely comparable (Table S11).

The HUAS JQ3^T^ genome also contained biosynthetic gene clusters associated with cyclodipeptide synthases (CDPSs), ectoine, class I lanthipeptides, class III lanthipeptides, lassopeptides, NRPS-independent siderophores (NI-siderophores), non-ribosomal peptide metallophores (NRP-metallophores), non-ribosomal peptide synthetases (NRPSs), oligosaccharides, phenazines, phosphonates, PKS-like compounds, RiPP-like compounds, type I polyketide synthases (T1PKS), type II polyketide synthases (T2PKS), terpenes, and other secondary metabolite-related proteins that could not be assigned to known categories (Supplementary Table S12). Genes associated with antibiotic resistance included the *Streptomyces venezuelae rox* gene and the *vanY* gene within the *vanF* cluster, which may confer resistance to rifamycin and glycopeptide antibiotics, respectively. Whether it has resistance or not needs to be further confirmed by experiments. In addition, 39 CRISPR repeats were identified in the HUAS JQ3^T^ genome. Finally, nine genomic islands ranging in size from 7000 to 50,696 bp were detected.

#### 3.2.4. Phylogeny and DNA–DNA Correlation Analysis

The full-length 16S rRNA gene sequence of strain HUAS JQ3^T^ was obtained from the whole-genome sequence and analyzed using the EzBioCloud server. The results indicated that this strain belongs to the genus *Nocardiopsis* and showed the highest sequence similarities to *N. dassonvillei* subsp. *crassaminis* D1^T^ (100.00%), *N. alborubida* NBRC 13392^T^ (99.86%), *N. synnemataformans* DSM 44143^T^ (99.73%), *N. akebiae* HDS 12^T^ (99.73%), *N. dassonvillei* subsp. *dassonvillei* DSM 43111^T^ (99.52%), *N. deserti* H13^T^ (99.45%), *N. lucentensis* DSM 44048^T^ (98.90%), *N. halotolerans* DSM 44410^T^ (98.83%), and *N. flavescens* CGMCC 4.5723^T^ (98.76%), whereas similarities to other type strains were ≤98.70%.

**Figure 5:**
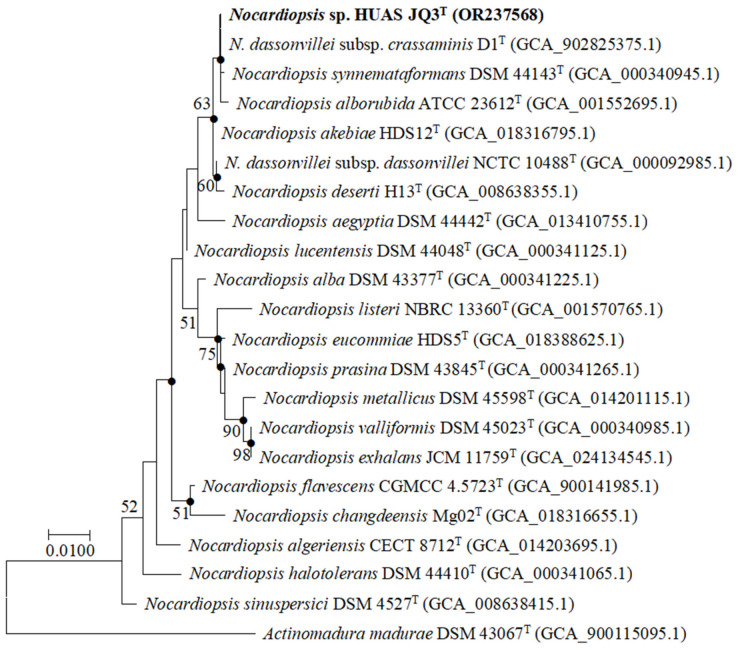
Maximum-likelihood phylogenetic tree based on 16S rRNA gene sequences showing the relationship between selected species of the genus *Nocardiopsis*. *Actinomadura madurae* DSM 43067^T^ was used as an outgroup. Bootstrap percentages over 50% derived from 1000 replications are shown at the node. Dots indicate branches that were also recovered in the neighbor-joining and maximum-parsimony trees. The scale bar represents 0.0100 changes per nucleotide position.

**Figure 6:**
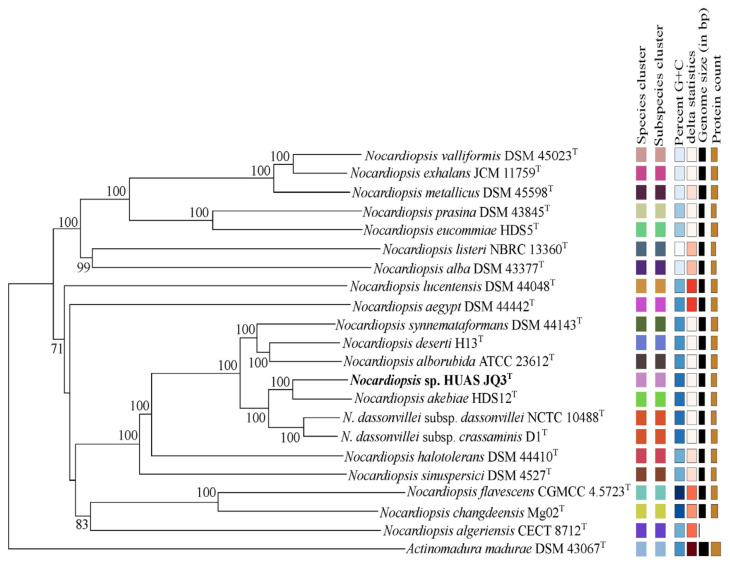
Phylogenetic tree based on whole genome sequences of HUAS JQ3^T^ and related reference strains. Tree inferred with FastME 2.1.6.1 [48] from GBDP distances calculated from genome sequences. The branch lengths are scaled in terms of GBDP distance formula d5. The numbers above branches are GBDP pseudo-bootstrap support values > 60% from 100 replications, with an average branch support of 96.0%. The tree was rooted at the midpoint [49].

The ML phylogenetic tree based on 16S rRNA gene sequences showed that strain HUAS JQ3^T^ was clustered with *N. alborubida* NBRC 13392^T^, *N. synnemataformans* DSM 44143^T^, and *N. dassonvillei* subsp. *crassaminis* D1^T^ (Figure 5). Similar topologies were also observed in the NJ and MP trees (Supplementary Figures S2 and S3). However, the phylogenomic tree based on whole-genome sequences indicated that strain HUAS JQ3^T^ was more closely related to *N. akebiae* HDS 12^T^ (Figure 6).

The dDDH and ANIm/ANIb values between strain HUAS JQ3^T^ and *N. akebiae* HDS 12^T^ were below the proposed species delineation thresholds of 70% dDDH and 96.68%/96.15% ANIm/b (Table 2). Because strain HUAS JQ3^T^ shared ≥98.7% 16S rRNA gene sequence similarity with several closely related type strains, including *N. dassonvillei* subsp. *crassaminis* D1^T^, *N. alborubida* NBRC 13392^T^, *N. synnemataformans* DSM 44143^T^, *N. dassonvillei* subsp. *dassonvillei* DSM 43111^T^, *N. deserti* H13^T^, *N. lucentensis* DSM 44048^T^, *N. halotolerans* DSM 44410^T^, and *N. flavescens* CGMCC 4.5723^T^, ANI and dDDH analyses were further performed, as recommended by Stackebrandt and Ebers (2006) [47]. The dDDH and ANIm/ANIb values between strain HUAS JQ3^T^ and these related strains ranged from 24.30 to 61.20% and 86.36 to 95.51%/79.99 to 94.62%, respectively, all below the species delineation thresholds (Table 2).

## 4. Discussion

### 4.1. Relationship Between ANI and dDDH Values in the Genus Nocardiopsis

Currently, ANI values of 95–96% [17] and dDDH values of 70% [16] are widely recognized as the gold standards for bacterial species delineation. However, as noted above, the taxonomic classification of strain HUAS JQ3^T^ remained contradictory when assessed against different criteria. To address this discrepancy, we propose that, given the continuous advancement of sequencing technologies, the computational accuracy and the associated ANI thresholds may warrant re-evaluation. Therefore, it is necessary to re-evaluate the ANI classification threshold for the genus *Nocardiopsis* and examine its correspondence with the 70% dDDH threshold, thereby providing a more reliable basis for clarifying the taxonomic status of the novel species. Obviously, in this work, the models fitted using three genome quality standards all produced good correlations, but the model fitted using the 13 high-quality genomes showed the strongest correlation. Therefore, we recommended using genomes with >90% completeness and <5% contamination as the standard for whole-genome quality assessment. Based on these results, we preliminarily proposed 96.68% ANIm and 96.15% ANIb as the classification thresholds for the genus *Nocardiopsis*. In addition, it should be noted that the thresholds proposed in this study are preliminary estimates based on currently available genome data and the exponential decay model. Future studies with more comprehensive datasets and alternative fitting approaches may further refine these values.

### 4.2. Evaluation of the Taxonomic Status of Strain HUAS JQ3^T^

According to the description of Yassin et al. [50], all of the *Nocardiopsis* species contain *meso*-DAP in their peptidoglycan and no characteristic sugars in whole-cell hydrolysates, have a type PIII phospholipid pattern (phosphatidylcholine is the characteristic phospholipid), contain characteristic menaquinones with 10 isoprene units, and have fatty acid profiles which include saturated, unsaturated, iso, and anteiso acids and tuberculostearic acid. However, in this work, phosphatidylcholine, as the characteristic phospholipid, was not detected in the polar lipid composition of strain HUAS JQ3^T^. We think that this phenomenon can be attributed to interspecies differences, which form the foundation of biodiversity. More precisely, the chemotaxonomic analyses supported the assignment of strain HUAS JQ3^T^ to the genus of *Nocardiopsis*.

The phylogenetic tree based on 16S rRNA gene sequences showed that strain HUAS JQ3^T^ clustered with *N. alborubida* NBRC 13392^T^, *N. synnemataformans* DSM 44143^T^, and *N. dassonvillei* subsp. *crassaminis* D1^T^. However, the phylogenetic tree based on whole-genome sequences indicated that strain HUAS JQ3^T^ was more closely related to *N. akebiae* HDS 12^T^. This finding further demonstrated that 16S rRNA gene sequence-based phylogenetic analysis provides lower resolution than genome-based phylogenetic analysis [51]. This also implies the 16S rRNA gene has limitations in identification of the genus *Nocardiopsis*. The dDDH and ANIm/ANIb values between strain HUAS JQ3^T^ and *N. akebiae* HDS 12^T^ were below the proposed species delineation thresholds of 70% dDDH and 96.68%/96.15% ANIm/b, strengthening strain HUAS JQ3^T^ as a new species of the genus *Nocardiopsis*.

Comparative genomic analyses showed differences between strain HUAS JQ3^T^ and *N. akebiae* HDS 12^T^. The biosynthetic gene clusters (including NRPS, deazapurine, lassopeptide and phosphonate) of strain HUAS JQ3^T^ are more abundant than that of *N. akebiae* HDS 12^T^. These findings are consistent with *Actinomycetota* being a major source of natural products. The biosynthetic gene clusters, nonribosomal peptide synthetases (NRPS), deazapurine, lassopeptide and phosphonate may be associated with antibiotics and other active substance [52].

In addition, morphological, cultural, physiological, and biochemical analysis demonstrated that strain HUAS JQ3^T^ differs from *N. akebiae* HDS 12^T^. For example, strain HUAS JQ3^T^ forms an ivory yellow aerial mycelium and dark greenish-olive substrate mycelium were well produced on Gause’s synthetic No. 1 medium, while *N. akebiae* HDS 12^T^ develops white aerial mycelium and lasbella color substrate mycelium. Strain HUAS JQ3^T^ could utilize cellulose, starch, sucrose and trehalose as sole carbon sources and could utilize l-glutamic acid, l-hydroxyproline, l-ornithine, l-phenylalanine and p-hydroxyphenylalanine as sole nitrogen sources. While *N. akebiae* HDS 12^T^ could not utilize cellulose, starch, sucrose and trehalose as sole carbon sources and could not utilize l-glutamic acid, l-hydroxyproline, l-ornithine, l-phenylalanine and p-hydroxyphenylalanine as sole nitrogen sources.

## 5. Conclusions

### 5.1. Relationship Between ANI and dDDH Values in the Genus Nocardiopsis

We recommended using genomes with >90% completeness and <5% contamination as the standard for whole-genome quality assessment, and we preliminarily estimated thresholds based on currently available genomes 96.68% ANIm and 96.15% ANIb as the classification thresholds for the genus *Nocardiopsis*.

### 5.2. Description of Nocardiopsis camelliae sp. nov.

Taken together, the genotypic and phenotypic evidence supports the conclusion that strain HUAS JQ3^T^ represents a novel species of the genus *Nocardiopsis*, for which the name *Nocardiopsis camelliae* sp. nov. is proposed.

*Nocardiopsis camelliae* (ca.mel’li.ae. N.L. gen. fem. n. *camelliae*, of *Camellia*, referring to the strain isolated from leaves of *Camellia oleifera* Abel).

Light drab diffusible pigments are produced on ISP 2 medium and deep olive-buff diffusible pigments are produced on ISP 4 medium. Ivory yellow aerial mycelium and dark greenish-olive substrate mycelium are produced on Gause’s synthetic No. 1 medium. Spore chains are slightly flexuous consisting of rod-shaped spores with a smooth surface. Growth occurs at 15–40 °C and pH 6.0–9.0, optimally at 28 °C and pH 7.0. Cells can tolerate up to 13.0% NaCl (*w*/*v*) on ISP 2 medium, with optimal growth at 0–1.0% NaCl. Cellulose, d-fructose, d-ribose, d-xylose, l-arabinose, l-rhamnose and *myo*-inositol, starch, sucrose, and trehalose can be utilized as sole carbon sources, while d-galactose, d-mannose, maltose, lactose, and raffinose are not utilized. The following compounds are utilized as sole nitrogen sources: l-cysteine, l-glutamic acid, l-hydroxyproline, l-ornithine, l-phenylalanine and p-hydroxyphenylalanine, whereas glycine, histidine, l-asparagine, and l-alanine are not utilized. The predominant cellular fatty acids are *iso*-C_16:0,_ C_18:1_ ω9c and C_18:0_ 10-methyl (TBSA). Starch hydrolysis, nitrate reduction and Tweens (20, 40, 60, 80) decomposition are negative and gelatin liquefaction is positive. The cell-wall diamino acid is *mes*-DAP, with no diagnostic sugars. The menaquinones are MK-10(H_2_), MK-10(H_4_) and MK-10(H_6_). Polar lipids comprise diphosphatidylglycerol (DPG), phosphatidylinositol mannosides (PIMs) and unidentified lipids (L1 and L2).

The type strain, strain HUAS JQ3^T^ (= MCCC 1K08696^T^ = JCM 36305^T^), was isolated from leaves of *Camellia oleifera* Abel. The NCBI accession numbers for the 16S rRNA gene sequence and genome sequence of strain HUAS JQ3^T^ are OR237566 and CP118614, respectively. The raw reads of strain HUAS JQ3^T^ are available in the SRA (Sequence Read Archive) under NCBI accession number PRJNA936281.

## Figures and Tables

**Table 1:** Characteristics of differentiating strains HUAS JQ3^T^ and *N. akebiae* HDS 12^T^.

Characteristics	1	2
Spore chain	Slightly flexuous	Straight to flexuous
Spore shape	Rod	Rod
Spore surface	Smooth	Smooth
Use of carbon sources:		
Cellulose, starch	+	–
d-Galactose, maltose	–	+
Sucrose, trehalose	+	–
Use of nitrogen source:		
Histidine	–	+
l-Glutamic acid, l-hydroxyproline	+	–
l-Ornithine, l-phenylalanine	+	–
p-Hydroxyphenylalanine	+	–
Starch hydrolysis	–	+
Temperature range (optimum, °C)	15–40 (28)	20–37 (28)
pH range (optimum)	6.0–9.0 (7.0)	6.0–8.0 (7.0)
NaCl tolerance (*w*/*v*) (optimum)	0–13.0% (0–1.0%)	0–11.0% (0–1.0%)
Gelatin liquefaction	+	–
Cell-wall diamino acid	*mes*-DAP	*mes*-DAP
Whole-cell sugars	Not detected	Not detected
Menaquinones	MK-10(H_2_) (50.3%) MK-10(H_4_) (34.7%) MK-10(H_6_) (12.4%)	MK-10(H_2_) (45.6%) MK-10(H_4_) (30.9%) MK-10(H_6_) (22.1%) ^a^
Polar lipids	DPG, PIM, L1, L2	DPG, PE, OH-PE, PC, PIM, PLS, NPG, N1, N2 ^a^

Note: 1, HUAS JQ3^T^; 2, *N. akebiae* HDS 12^T^. +, positive; –, negative. All data are from this study unless otherwise indicated. ^a^ Data are from Mo et al. (2022) [46]. All strains can utilize d-fructose, d-ribose, d-xylose l-arabinose, l-rhamnose and *myo*-inositol as sole carbon sources, but not d-mannose, lactose and raffinose. All strains were able to use l-cysteine as a nitrogen source; but not glycine, l-asparagine and l-alanine. All the strains were negative for nitrate reduction and Tweens (20, 40, 60, 80) decomposition. The polar lipids comprised diphosphatidylglycerol (DPG), phosphatidylethanolamine (PE), hydroxyl phosphatidylethanolamine (OH-PE), phosphatidyl choline (PC), phosphatidylinositol mannosides (PIMs), phospholipids (PLs), phospholipids of unknown structure containing glucosamine (NPG), unidentified lipid (L1, L2) and unidentified phospholipids (N1 and N2).

**Table 2:** Comparative genotypic analysis between strain HUAS JQ3^T^ and the closest related type strains, *N. dassonvillei* subsp. *crassaminis* D1^T^, *N. alborubida* NBRC 13392^T^, *N. synnemataformans* DSM 44143^T^, *N. akebiae* HDS 12^T^, *N. dassonvillei* subsp. *dassonvillei* DSM 43111T, *N. deserti* H13^T^, *N. lucentensis* DSM 44048^T^, *N. halotolerans* DSM 44410^T^ and *N. flavescens* CGMCC 4.5723^T^.

Closest Related Species	Strain HUAS JQ3^T^			
	16S rRNA Gene Sequence Similarity	ANIm (%)	ANIb (%)	dDDH (%)
*N. dassonvillei* subsp. *crassaminis* D1^T^	100.00%	95.51	94.51	61.20
*N. alborubida* NBRC 13392^T^	99.86%	93.09	93.03	51.60
*N. synnemataformans* DSM 44143^T^	99.73%	94.04	93.22	51.70
*N. akebiae* HDS 12^T^	99.73%	96.46	96.04	68.40
*N. dassonvillei* subsp. *dassonvillei* DSM 43111^T^	99.52%	95.43	94.62	60.50
*N. deserti* H13^T^	99.45%	93.89	92.91	51.10
*N. lucentensis* DSM 44048^T^	98.90%	86.36	79.99	25.10
*N. halotolerans* DSM 44410^T^	98.83%	89.39	86.67	33.80
*N. flavescens* CGMCC 4.5723^T^	98.76%	86.13	80.29	24.30

## Data Availability

Data will be made available on request.

## Supplementary Materials

The following supporting information can be downloaded at https://www.mdpi.com/article/10.3390/biology15141119/s1: Figure S1. Polar lipids composition of strain HUAS JQ3^T^. Figure S2. Neighbor-joining phylogenetic tree based on 16S rRNA gene sequences showing the relationship between selected species of the genus *Nocardiopsis*. Figure S3. Maximum-parsimony phylogenetic tree based on 16S rRNA gene sequences showing the relationship between selected species of the genus *Nocardiopsis*. Table S1. Quality analysis and GenBank assembly of genomes of *Nocardiopsis* species in this work (40 strains). Table S2. Quality analysis and GenBank assembly of genomes of *Nocardiopsis* species in this work (13 strains). Table S3. Quality analysis and GenBank assembly of genomes of *Nocardiopsis* species in this work (34 strains). Table S4. ANI and dDDH values of 78 pairs of *Nocardiopsis* species. Table S5. ANI and dDDH values of 561 pairs of *Nocardiopsis* species. Table S6. ANI and dDDH values of 780 pairs of *Nocardiopsis* species. Table S7. Estimated ANIm and ANIb species delineation thresholds for the genus *Nocardiopsis* based on different genome quality criteria. Table S8. Cultural characteristics of HUAS JQ3^T^ and *N. akebiae* HDS 12^T^. Table S9. The fatty acid composition of strains HUAS JQ3^T^ and *N. akebiae* HDS 12^T^. Table S10. Genome features of strains HUAS JQ3^T^ and *N. akebiae* HDS 12^T^. Table S11. The subsystem category number of genes of strains HUAS JQ3^T^ and *N. akebiae* HDS 12^T^ based on RAST annotation server. Table S12. The distribution of biosynthetic gene clusters in the genome of strains HUAS JQ3^T^ and *N. akebiae* HDS 12^T^ by antiSMASH analyses.

## Abbreviations

MCCC	Marine Culture Collection of China
JCM	the Japan Collection of Microorganisms
ISP	International *Streptomyces* Project
