# Supplementary material for: Genomic Insights into ANI-dDDH Relationships in Nocardiopsis and the Novel Species Nocardiopsis camelliae sp. nov
Source: Biology (Basel). 2026 Jul 10;15(14):1119. doi: 10.3390/biology15141119 (PMC13405408; doi:10.3390/biology15141119)
Supplement: Supplementary file 1 [file biology-15-01119-s001.zip › biology-4392736-supplementary.pdf]

Genomic Insights into ANI-dDDH Relationships in *Nocardiopsis* and the Novel Species *Nocardiopsis camelliae* sp. nov.

Ting Tang[1†], Wenguan Huang[1, 2†], Huiping Zhong[1, 2], Ping Mo[1, 2*],

Yaxi Zheng[1], Li Fu[3], Kaiqin Li[4*] and Jian Gao[5]

Author affiliations:

[1]Science and Technology Innovation Team for Efficient Agricultural Production and Deep Processing at General University in Hunan Province, College of Life and Environmental Sciences, Hunan University of Arts and Science, Changde 415000, Hunan Province, China.

[2]College of Furong, Hunan University of Arts and Science, Changde 415000, Hunan Province, China.

[3]College of Life Sciences, Wuhan University, Wuhan 430072, Hubei Province, China.

[4]School of Computer Science and Engineering, Hunan University of Science and Technology, Xiangtan 411201, Hunan Province, China.

[5]School of Life and Health Sciences, Hunan University of Science and Technology, Xiangtan 411201, Hunan Province, China.

†These authors contributed equally to this work.

[*]Correspondence:

Ping Mo, moping2015@126.com; Kaiqin Li, likaiqin2425@hnust.edu.cn

Figure S1. Polar lipids composition of strain HUAS JQ3^T.

The plate dotted with sample was subjected to two-dimensional development, with the first solvent of chloroform-methanol-water (65:25:4, v/v/v) followed by the second solvent of chloroform-methanol-acetic acid-water (80:18:12:5, v/v/v/v).

Molybdophosphoric acid, molybdenum blue reagent, anisaldehyde and ninhydrin were used to detect total lipids, phospholipids, phosphatidylinositol mannosides and aminolipids respectively. A, Molybdophosphoric acid (for total lipids); B, Molybdenum blue reagent (for phospholipids); C, Anisaldehyde (for phosphatidylinositol mannosides); D, Ninhydrin (for aminolipids). DPG, diphosphatidylglycerol; PIM, phosphatidylinositol mannosides, L1, L2, unidentified lipid.

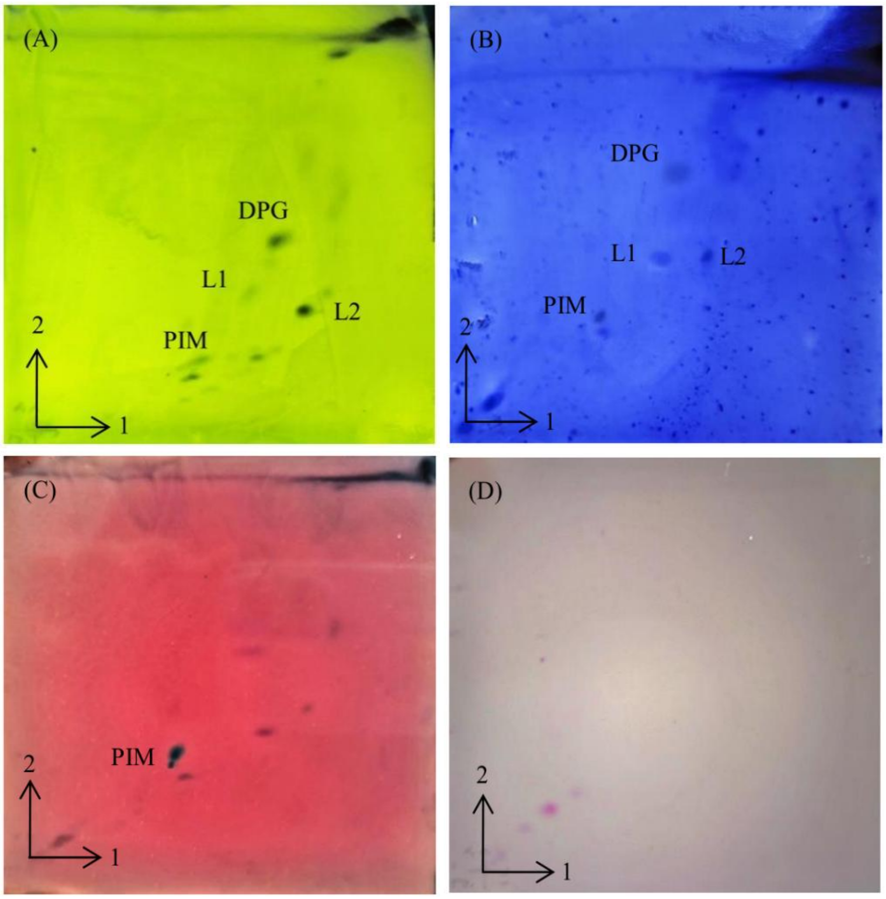

Figure S2. Neighbor-joining phylogenetic tree based on 16S rRNA gene sequences showing the relationship between selected species of the genus *Nocardiopsis*. *Actinomadura madurae* DSM 43067^T was used as an outgroup. Bootstrap percentages over 50% derived from 1,000 replications are shown at the node. Bar represents 0.0100 changes per nucleotide position

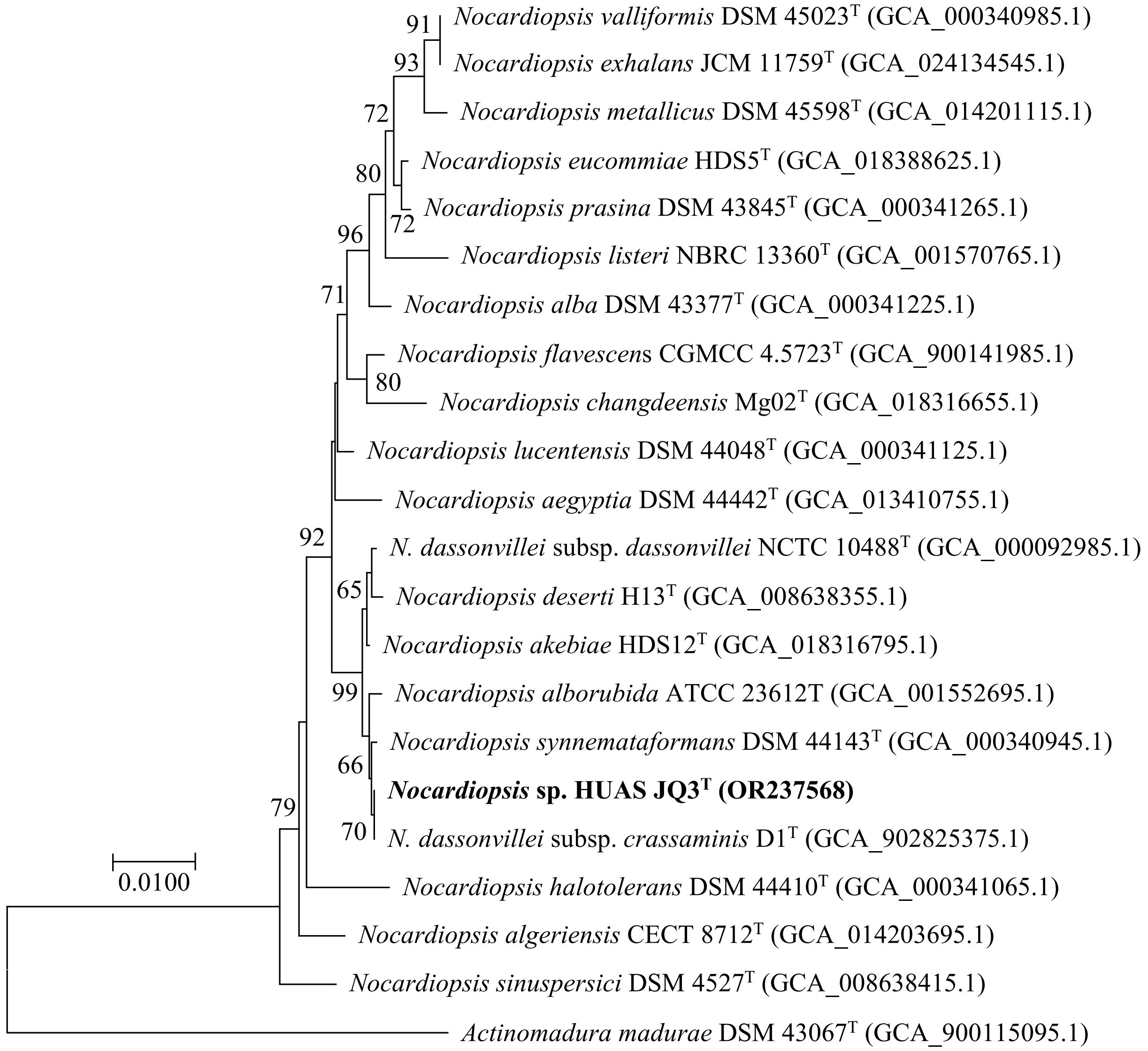

Figure S3. Maximum-parsimony phylogenetic tree based on 16S rRNA gene sequences showing the relationship between selected species of the genus *Nocardiopsis*. *Actinomadura madurae* DSM 43067^T was used as an outgroup. Bootstrap percentages over 50% derived from 1,000 replications are shown at the node. Bar represents 10 changes per nucleotide position

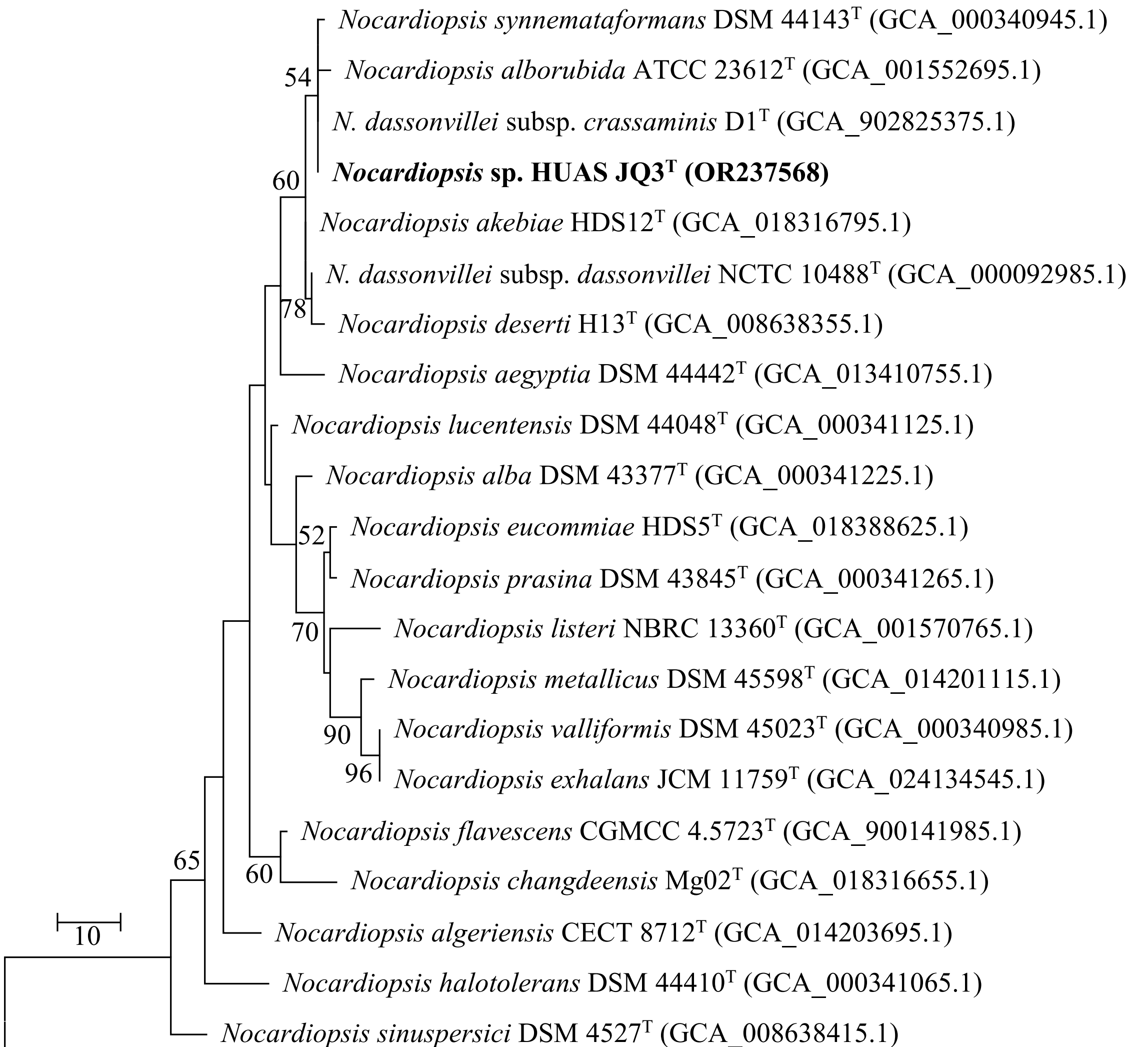

Table S1. Quality analysis and GenBank assembly of genomes of *Nocardiopsis* species in this work (40 strains).

No.	Type strain	GenBank assembly	COM (%)	CON (%)	Genome size (bp)	Genome coverage	Genes total
1.	*Nocardiopsis aegyptia* DSM 44442^T	GCA_013410755.1	96.38	8.97	6909152	138.0x	6,183
2.	*Nocardiopsis akebiae* HDS12^T	GCA_018316795.1	99.25	1.86	6602217	120.0x	5,704
3.	*Nocardiopsis alba* DSM 43377^T	GCA_000341225.1	98.97	1.83	5820411	153.0x	5,178
4.	*Nocardiopsis alborubida* ATCC 23612^T	GCA_012396365.1	99.18	5.35	7158456	139.0x	6,333
5.	*Nocardiopsis algeriensis* CECT 8712^T	GCA_014203695.1	91.76	1.1	4811278	311.0x	4,257
6.	*Nocardiopsis alkaliphila* YIM 80379^T	GCA_000341005.1	92.32	3.37	5209552	100.0x	4,759
7.	*Nocardiopsis arvandica* DSM 45278^T	GCA_013410445.1	96.56	5.12	6203486	156.0x	5,441
8.	*Nocardiopsis baichengensis* YIM 90130^T	GCA_000341205.1	92.64	9.15	6404110	106.0x	5,625
9.	*Nocardiopsis changdeensis* Mg02^T	GCA_018316655.1	97.38	8.5	144773	120.0x	6,687
10.	*Nocardiopsis codii* CT-R113^T	GCA_036327455.1	96.56	6.32	7240724	56.0x	6,365
11.	*Nocardiopsis coralli* HNM0947^T	GCA_014892575.1	94.07	6.74	6398852	180.0x	5,566
12.	*Nocardiopsis dassonvillei* subsp. *crassaminis* D1^T	GCA_902825375.1	98.25	1.93	6655292	ND	6,123
13.	*Nocardiopsis dassonvillei* subsp. *dassonvillei* DSM 43111^T	GCA_000092985.1	99.48	0.31	6541269	100.0x	5623
14.	*Nocardiopsis deserti* H13^T	GCA_008638355.1	98.84	4.7	7251566	100.0x	6,334
15.	*Nocardiopsis exhalans* JCM11759^T	GCA_024134545.1	96.92	6.3	7390049	130.0x	6,695

16.	*Nocardiopsis flavescens* CGMCC 4.5723^T	GCA_900141985.1	96.69	6.58	7171718	100.0x	6,251
17.	*Nocardiopsis ganjiahuensis* DSM 45031^T	GCA_000341085.1	98	7.12	7352287	105.0x	6,511
18.	*Nocardiopsis gilva* YIM 90087^T	GCA_002263495.1	88.58	6.73	6142152	208.0x	5,553
19.	*Nocardiopsis halophila* DSM 44494^T	GCA_000341245.1	89.48	8.55	6320069	90.0x	5,730
20.	*Nocardiopsis halotolerans* DSM 44410^T	GCA_000341065.1	92.94	5.58	6263929	65.0x	5,698
21.	*Nocardiopsis kunsanensis* DSM 44524^T	GCA_000340965.1	92	4.27	5312089	105.0x	4,690
22.	*Nocardiopsis lambiniae* DSM 44743^T	GCA_031845465.1	98.26	5.12	6249249	51.2773x	5,516
23.	*Nocardiopsis listeri* NBRC 13360^T	GCA_001570765.1	96.04	3.9	5685516	89.0x	5,103
24.	*Nocardiopsis litoralis* KCTC 19473^T	GCA_014651735.1	91.59	4.3	5188564	26.0x	4,628
25.	*Nocardiopsis lucentensis* DSM 44048^T	GCA_000341125.1	89.84	6.5	6325274	65.0x	6,290
26.	*Nocardiopsis mangrovi* CGMCC 4.7119^T	GCA_042653925.1	91.17	9.79	6986671	78.0x	6,032
27.	*Nocardiopsis metallicus* JCM 12409^T	GCA_039527755.1	97.64	8.68	7461741	82.0x	6,726
28.	*Nocardiopsis mwathae* DSM 46659^T	GCA_014201195.1	89.68	6.33	5936987	324.0x	5,270
29.	*Nocardiopsis potens* DSM 45234^T	GCA_000341105.1	92.66	8.82	6909969	95.0x	5,973
30.	*Nocardiopsis prasina* DSM 43845^T	GCA_000341265.1	94.04	5.17	5998803	120.0x	5,414
31.	*Nocardiopsis quinghaiensis* YIM 28A4^T	GCA_008638365.1	96.26	4.78	5915507	100.0x	5,216
32.	*Nocardiopsis rhodophaea* JCM 15313^T	GCA_039530945.1	90.54	8.47	5906158	102x	5,198
33.	*Nocardiopsis salina* YIM 90010^T	GCA_000341025.1	88.41	6.73	5808301	75.0x	5,348

34.	*Nocardiopsis sediminis* TBRC 1826^{T}	GCA_042650565.1	91.31	6.49	6673485	100x	5,877
35.	*Nocardiopsis sinuspersici* UTMC 00102^{T}	GCA_001998325.1	96.56	3.02	6114734	106.0x	5,355
36.	*Nocardiopsis terrae* KCTC 19431^{T}	GCA_014651695.1	98.87	3.74	6003841	162.0x	5,236
37.	*Nocardiopsis trehalosi* NBRC 14201^{T}	GCA_001552555.1	93.5	6.67	6379550	147.0x	5,710
38.	*Nocardiopsis tropica* JCM 10877^{T}	GCA_042660575.1	98.43	6.14	297158	55.0x	6,731
39.	*Nocardiopsis valliformis* DSM 45023^{T}	GCA_000340985.1	97.05	7.41	6003841	162.0x	5,236
40	*Nocardiopsis xinjiangensis* YIM 90004^{T}	GCA_000341145.1	88.84	4.93	5330432	130.0x	4,722

Note: COM, Completeness; CON, Contamination; ND, not detected.

Table S2. Quality analysis and GenBank assembly of genomes of *Nocardiopsis* species in this work (13 strains).

No.	Type strain	GenBank assembly	COM (%)	CON (%)
1.	*Nocardiopsis akebiae* HDS12^T	GCA_018316795.1	99.25	1.86
2.	*Nocardiopsis alba* DSM 43377^T	GCA_000341225.1	98.97	1.83
3.	*Nocardiopsis algeriensis* CECT 8712^T	GCA_014203695.1	91.76	1.1
4.	*Nocardiopsis alkaliphila* YIM 80379^T	GCA_000341005.1	92.32	3.37
5.	*Nocardiopsis dassonvillei* subsp. *crassaminis* D1^T	GCA_902825375.1	98.25	1.93
6.	*Nocardiopsis dassonvillei* subsp. *dassonvillei* DSM 43111^T	GCA_000092985.1	99.48	0.31
7.	*Nocardiopsis deserti* H13^T	GCA_008638355.1	98.84	4.7
8.	*Nocardiopsis kunsanensis* DSM 44524^T	GCA_000340965.1	92	4.27
9.	*Nocardiopsis listeri* NBRC 13360^T	GCA_001570765.1	96.04	3.9
10.	*Nocardiopsis litoralis* KCTC 19473^T	GCA_014651735.1	91.59	4.3
11.	*Nocardiopsis quinghaiensis* YIM 28A4^T	GCA_008638365.1	96.26	4.78
12.	*Nocardiopsis sinuspersici* UTMC 00102^T	GCA_001998325.1	96.56	3.02
13.	*Nocardiopsis terrae* KCTC 19431^T	GCA_014651695.1	98.87	3.74

Note: COM, Completeness; CON, Contamination.

Table S3. Quality analysis and GenBank assembly of genomes of *Nocardiopsis* species in this work (34 strains).

No.	Type strain	GenBank assembly	Completeness (%)	Contamination (%)
1.	*Nocardiopsis aegyptia* DSM 44442^T	GCA_013410755.1	96.38	8.97
2.	*Nocardiopsis akebiae* HDS12^T	GCA_018316795.1	99.25	1.86
3.	*Nocardiopsis alba* DSM 43377^T	GCA_000341225.1	98.97	1.83
4.	*Nocardiopsis alborubida* ATCC 23612^T	GCA_012396365.1	99.18	5.35
5.	*Nocardiopsis algeriensis* CECT 8712^T	GCA_014203695.1	91.76	1.1
6.	*Nocardiopsis alkaliphila* YIM 80379^T	GCA_000341005.1	92.32	3.37
7.	*Nocardiopsis arvandica* DSM 45278^T	GCA_013410445.1	96.56	5.12
8.	*Nocardiopsis baichengensis* YIM 90130^T	GCA_000341205.1	92.64	9.15
9.	*Nocardiopsis changdeensis* Mg02^T	GCA_018316655.1	97.38	8.5
10.	*Nocardiopsis codii* CT-R113^T	GCA_036327455.1	96.56	6.32
11.	*Nocardiopsis coralli* HNM0947^T	GCA_014892575.1	94.07	6.74
12.	*Nocardiopsis dassonvillei* subsp. *crassaminis* D1^T	GCA_902825375.1	98.25	1.93
13.	*Nocardiopsis dassonvillei* subsp. *dassonvillei* DSM 43111^T	GCA_000092985.1	99.48	0.31
14.	*Nocardiopsis deserti* H13^T	GCA_008638355.1	98.84	4.7
15.	*Nocardiopsis exhalans* JCM11759^T	GCA_024134545.1	96.92	6.3
16.	*Nocardiopsis flavescens* CGMCC 4.5723^T	GCA_900141985.1	96.69	6.58

17.	*Nocardiopsis ganjiahuensis* DSM 45031^T	GCA_000341085.1	98	7.12
18.	*Nocardiopsis halotolerans* DSM 44410^T	GCA_000341065.1	92.94	5.58
19.	*Nocardiopsis kunsanensis* DSM 44524^T	GCA_000340965.1	92	4.27
20.	*Nocardiopsis lambiniae* DSM 44743^T	GCA_031845465.1	98.26	5.12
21.	*Nocardiopsis listeri* NBRC 13360^T	GCA_001570765.1	96.04	3.9
22.	*Nocardiopsis litoralis* KCTC 19473^T	GCA_014651735.1	91.59	4.3
23.	*Nocardiopsis mangrovi* CGMCC 4.7119^T	GCA_042653925.1	91.17	9.79
24.	*Nocardiopsis metallicus* JCM 12409^T	GCA_039527755.1	97.64	8.68
25.	*Nocardiopsis potens* DSM 45234^T	GCA_000341105.1	92.66	8.82
26.	*Nocardiopsis prasina* DSM 43845^T	GCA_000341265.1	94.04	5.17
27.	*Nocardiopsis quinghaiensis* YIM 28A4^T	GCA_008638365.1	96.26	4.78
28.	*Nocardiopsis rhodophaea* JCM 15313^T	GCA_039530945.1	90.54	8.47
29.	*Nocardiopsis sediminis* TBRC 1826^T	GCA_042650565.1	91.31	6.49
30.	*Nocardiopsis sinuspersici* UTMC 00102^T	GCA_001998325.1	96.56	3.02
31.	*Nocardiopsis terrae* KCTC 19431^T	GCA_014651695.1	98.87	3.74
32.	*Nocardiopsis trehalosi* NBRC 14201^T	GCA_001552555.1	93.5	6.67
33.	*Nocardiopsis tropica* JCM 10877^T	GCA_042660575.1	98.43	6.14
34.	*Nocardiopsis valliformis* DSM 45023^T	GCA_000340985.1	97.05	7.41

Table S4. ANI and dDDH values of 78 pairs of *Nocardiopsis* species.

No.	Strain 1	Strain 2	ANIm	ANIb	dDDH
1.	*Nocardiopsis kunsanensis* DSM 44524	*Nocardiopsis litoralis* KCTC 19473	99.48	99.25	95.7
2.	*Nocardiopsis dassonvillei* subsp. *dassonvillei* DSM 43111	*Nocardiopsis dassonvillei* subsp. *crassaminis* D1	97.83	97.34	79.2
3.	*Nocardiopsis akebiae* HDS12	*Nocardiopsis dassonvillei* subsp. *crassaminis* D1	95.4	94.27	60.5
4.	*Nocardiopsis dassonvillei* subsp. *dassonvillei* DSM 43111	*Nocardiopsis akebiae* HDS12	95.35	94.54	60.1
5.	*Nocardiopsis deserti* H13	*Nocardiopsis dassonvillei* subsp. *crassaminis* D1	94.14	93.21	52.9
6.	*Nocardiopsis dassonvillei* subsp. *dassonvillei* DSM 43111	*Nocardiopsis deserti* H13	94.14	93.21	52.5
7.	*Nocardiopsis deserti* H13	*Nocardiopsis akebiae* HDS12	93.68	92.86	50.4
8.	*Nocardiopsis sinuspersici* UTMC 00102	*Nocardiopsis quinghaiensis* YIM 28A4	92.88	91.76	47.5
9.	*Nocardiopsis quinghaiensis* YIM 28A4	*Nocardiopsis dassonvillei* subsp. *crassaminis* D1	88.98	85.94	32.5
10.	*Nocardiopsis sinuspersici* UTMC 00102	*Nocardiopsis dassonvillei* subsp. *crassaminis* D1	89.02	86.38	32.5
11.	*Nocardiopsis deserti* H13	*Nocardiopsis quinghaiensis* YIM 28A4	88.9	86.16	32.3
12.	*Nocardiopsis sinuspersici* UTMC 00102	*Nocardiopsis deserti* H13	89.02	86.56	32.2
13.	*Nocardiopsis dassonvillei* subsp. *dassonvillei* DSM 43111	*Nocardiopsis sinuspersici* UTMC 00102	88.87	86.2	31.8
14.	*Nocardiopsis dassonvillei* subsp. *dassonvillei* DSM 43111	*Nocardiopsis quinghaiensis* YIM 28A4	88.81	85.84	31.7
15.	*Nocardiopsis sinuspersici* UTMC 00102	*Nocardiopsis akebiae* HDS12	88.72	85.91	31.6
16.	*Nocardiopsis quinghaiensis* YIM 28A4	*Nocardiopsis akebiae* HDS12	88.61	85.61	31.3
17.	*Nocardiopsis alkaliphila* YIM 80379	*Nocardiopsis listeri* NBRC 13360	86.54	82.73	26.3
18.	*Nocardiopsis algeriensis* CECT 8712	*Nocardiopsis dassonvillei* subsp. *crassaminis* D1	86.51	81.43	25.8
19.	*Nocardiopsis deserti* H13	*Nocardiopsis algeriensis* CECT 8712	86.34	81.35	25.5
20.	*Nocardiopsis dassonvillei* subsp. *dassonvillei* DSM 43111	*Nocardiopsis algeriensis* CECT 8712	86.39	81.36	25.3
21.	*Nocardiopsis algeriensis* CECT 8712	*Nocardiopsis akebiae* HDS12	86.26	81.2	25.2
22.	*Nocardiopsis terrae* KCTC 19431	*Nocardiopsis dassonvillei* subsp. *crassaminis* D1	86.33	80.24	25
23.	*Nocardiopsis alba* DSM 43377	*Nocardiopsis terrae* KCTC 19431	86.4	80.99	25

24.	*Nocardiopsis alba* DSM 43377	*Nocardiopsis listeri* NBRC 13360	86.23	80.89	24.9
25.	*Nocardiopsis alkaliphila* YIM 80379	*Nocardiopsis alba* DSM 43377	86.15	80.95	24.9
26.	*Nocardiopsis sinuspersici* UTMC 00102	*Nocardiopsis algeriensis* CECT 8712	86.2	81.19	24.9
27.	*Nocardiopsis listeri* NBRC 13360	*Nocardiopsis terrae* KCTC 19431	86.26	80.7	24.7
28.	*Nocardiopsis quinghaiensis* YIM 28A4	*Nocardiopsis algeriensis* CECT 8712	86.1	81	24.7
29.	*Nocardiopsis deserti* H13	*Nocardiopsis terrae* KCTC 19431	86.27	80.18	24.6
30.	*Nocardiopsis dassonvillei* subsp. *dassonvillei* DSM 43111	*Nocardiopsis terrae* KCTC 19431	86.2	80.01	24.5
31.	*Nocardiopsis alkaliphila* YIM 80379	*Nocardiopsis terrae* KCTC 19431	86.01	80.7	24.5
32.	*Nocardiopsis terrae* KCTC 19431	*Nocardiopsis akebiae* HDS12	86.17	79.92	24.4
33.	*Nocardiopsis sinuspersici* UTMC 00102	*Nocardiopsis terrae* KCTC 19431	86.23	80.29	24.3
34.	*Nocardiopsis alba* DSM 43377	*Nocardiopsis dassonvillei* subsp. *crassaminis* D1	85.89	78.92	24.1
35.	*Nocardiopsis quinghaiensis* YIM 28A4	*Nocardiopsis terrae* KCTC 19431	86.15	80.3	24.1
36.	*Nocardiopsis alba* DSM 43377	*Nocardiopsis deserti* H13	85.78	78.74	23.7
37.	*Nocardiopsis listeri* NBRC 13360	*Nocardiopsis dassonvillei* subsp. *crassaminis* D1	85.6	78.84	23.7
38.	*Nocardiopsis alkaliphila* YIM 80379	*Nocardiopsis dassonvillei* subsp. *crassaminis* D1	85.54	78.57	23.5
39.	*Nocardiopsis alba* DSM 43377	*Nocardiopsis akebiae* HDS12	85.84	78.67	23.5
40.	*Nocardiopsis dassonvillei* subsp. *dassonvillei* DSM 43111	*Nocardiopsis alba* DSM 43377	85.89	78.81	23.5
41.	*Nocardiopsis alba* DSM 43377	*Nocardiopsis sinuspersici* UTMC 00102	85.71	78.71	23.4
42.	*Nocardiopsis listeri* NBRC 13360	*Nocardiopsis deserti* H13	85.59	78.66	23.3
43.	*Nocardiopsis alkaliphila* YIM 80379	*Nocardiopsis akebiae* HDS12	85.51	78.24	23.2
44.	*Nocardiopsis listeri* NBRC 13360	*Nocardiopsis akebiae* HDS12	85.55	78.61	23.2
45.	*Nocardiopsis dassonvillei* subsp. *dassonvillei* DSM 43111	*Nocardiopsis listeri* NBRC 13360	85.55	78.64	23.2
46.	*Nocardiopsis litoralis* KCTC 19473	*Nocardiopsis dassonvillei* subsp. *crassaminis* D1	85.29	78.13	23.1
47.	*Nocardiopsis dassonvillei* subsp. *dassonvillei* DSM 43111	*Nocardiopsis alkaliphila* YIM 80379	85.49	78.37	23.1
48.	*Nocardiopsis alkaliphila* YIM 80379	*Nocardiopsis deserti* H13	85.4	78.45	23.1

49.	*Nocardiopsis listeri* NBRC 13360	*Nocardiopsis sinuspersici* UTMC 00102	85.57	78.52	23.1
50.	*Nocardiopsis alba* DSM 43377	*Nocardiopsis quinghaiensis* YIM 28A4	85.7	78.53	23.1
51.	*Nocardiopsis algeriensis* CECT 8712	*Nocardiopsis terrae* KCTC 19431	85.55	79.21	23.1
52.	*Nocardiopsis kunsanensis* DSM 44524	*Nocardiopsis dassonvillei* subsp. *crassaminis* D1	85.27	77.91	23
53.	*Nocardiopsis alkaliphila* YIM 80379	*Nocardiopsis sinuspersici* UTMC 00102	85.42	78.23	22.9
54.	*Nocardiopsis listeri* NBRC 13360	*Nocardiopsis quinghaiensis* YIM 28A4	85.6	78.43	22.8
55.	*Nocardiopsis kunsanensis* DSM 44524	*Nocardiopsis deserti* H13	85.14	77.64	22.7
56.	*Nocardiopsis alkaliphila* YIM 80379	*Nocardiopsis quinghaiensis* YIM 28A4	85.36	78.17	22.7
57.	*Nocardiopsis litoralis* KCTC 19473	*Nocardiopsis akebiae* HDS12	85.12	77.93	22.6
58.	*Nocardiopsis deserti* H13	*Nocardiopsis litoralis* KCTC 19473	85.12	77.95	22.6
59.	*Nocardiopsis kunsanensis* DSM 44524	*Nocardiopsis akebiae* HDS12	85.11	77.7	22.5
60.	*Nocardiopsis dassonvillei* subsp. *dassonvillei* DSM 43111	*Nocardiopsis litoralis* KCTC 19473	85.29	77.86	22.4
61.	*Nocardiopsis alba* DSM 43377	*Nocardiopsis algeriensis* CECT 8712	85.6	78.2	22.4
62.	*Nocardiopsis sinuspersici* UTMC 00102	*Nocardiopsis litoralis* KCTC 19473	85.13	77.89	22.3
63.	*Nocardiopsis alkaliphila* YIM 80379	*Nocardiopsis algeriensis* CECT 8712	85.22	77.6	22.2
64.	*Nocardiopsis kunsanensis* DSM 44524	*Nocardiopsis sinuspersici* UTMC 00102	85.11	77.62	22.2
65.	*Nocardiopsis dassonvillei* subsp. *dassonvillei* DSM 43111	*Nocardiopsis kunsanensis* DSM 44524	85.26	77.73	22.2
66.	*Nocardiopsis quinghaiensis* YIM 28A4	*Nocardiopsis litoralis* KCTC 19473	85.15	77.75	22.2
67.	*Nocardiopsis listeri* NBRC 13360	*Nocardiopsis algeriensis* CECT 8712	85.32	78.14	22.2
68.	*Nocardiopsis kunsanensis* DSM 44524	*Nocardiopsis quinghaiensis* YIM 28A4	85.12	77.47	22.1
69.	*Nocardiopsis kunsanensis* DSM 44524	*Nocardiopsis terrae* KCTC 19431	85.08	77.2	21.9
70.	*Nocardiopsis terrae* KCTC 19431	*Nocardiopsis litoralis* KCTC 19473	85.14	77.33	21.9
71.	*Nocardiopsis alkaliphila* YIM 80379	*Nocardiopsis litoralis* KCTC 19473	84.89	76.44	21.5
72.	*Nocardiopsis kunsanensis* DSM 44524	*Nocardiopsis listeri* NBRC 13360	85	76.74	21.5
73.	*Nocardiopsis listeri* NBRC 13360	*Nocardiopsis litoralis* KCTC 19473	85.01	76.94	21.5

74.	*Nocardiopsis kunsanensis* DSM 44524	*Nocardiopsis algeriensis* CECT 8712	85.07	77.14	21.5
75.	*Nocardiopsis algeriensis* CECT 8712	*Nocardiopsis litoralis* KCTC 19473	85.05	77.26	21.5
76.	*Nocardiopsis kunsanensis* DSM 44524	*Nocardiopsis alkaliphila* YIM 80379	84.9	76.36	21.4
77.	*Nocardiopsis kunsanensis* DSM 44524	*Nocardiopsis alba* DSM 43377	85.03	76.68	21.4
78	*Nocardiopsis alba* DSM 43377	*Nocardiopsis litoralis* KCTC 19473	85.08	76.84	21.4

Note: Completeness >90%, Contamination<5%.

Table S5. ANI and dDDH values of 561 pairs of *Nocardiopsis* species.

No.	Strain 1	Strain 2	ANIm	ANIb	dDDH
1.	*Nocardiopsis kunsanensis* DSM 44524	*Nocardiopsis litoralis* KCTC 19473	99.48	99.25	95.7
2.	*Nocardiopsis arvandica* DSM 45278	*Nocardiopsis sinuspersici* UTMC 00102	99.05	98.41	90.8
3.	*Nocardiopsis dassonvillei* subsp. *crassaminis* D1	*Nocardiopsis dassonvillei* subsp. *dassonvillei* DSM 43111	97.83	97.43	79.5
4.	*Nocardiopsis exhalans* JCM11759	*Nocardiopsis valliformis* DSM 45023	96.33	95.24	66.6
5.	*Nocardiopsis alborubida* ATCC 23612	*Nocardiopsis deserti* H13	95.58	94.61	62.2
6.	*Nocardiopsis akebiae* HDS12	*Nocardiopsis dassonvillei* subsp. *dassonvillei* DSM 43111	95.35	94.54	60.4
7.	*Nocardiopsis exhalans* JCM11759	*Nocardiopsis metallicus* JCM 12409	95.57	94.46	61.2
8.	*Nocardiopsis akebiae* HDS12	*Nocardiopsis dassonvillei* subsp. *crassaminis* D1	95.39	94.27	60.5
9.	*Nocardiopsis metallicus* JCM 12409	*Nocardiopsis valliformis* DSM 45023	94.98	93.67	57.7
10.	*Nocardiopsis alborubida* ATCC 23612	*Nocardiopsis dassonvillei* subsp. *dassonvillei* DSM 43111	94.27	93.54	53.6
11.	*Nocardiopsis alborubida* ATCC 23612	*Nocardiopsis dassonvillei* subsp. *crassaminis* D1	94.32	93.14	53.7
12.	*Nocardiopsis dassonvillei* subsp. *crassaminis* D1	*Nocardiopsis deserti* H13	94.21	92.67	52.9
13.	*Nocardiopsis dassonvillei* subsp. *dassonvillei* DSM 43111	*Nocardiopsis deserti* H13	94.14	92.63	52.6
14.	*Nocardiopsis akebiae* HDS12	*Nocardiopsis alborubida* ATCC 23612	93.72	92.32	50.5
15.	*Nocardiopsis akebiae* HDS12	*Nocardiopsis deserti* H13	93.68	92.28	50.4
16.	*Nocardiopsis arvandica* DSM 45278	*Nocardiopsis quinghaiensis* YIM 28A4	92.89	91.78	47.7
17.	*Nocardiopsis quinghaiensis* YIM 28A4	*Nocardiopsis sinuspersici* UTMC 00102	92.88	91.78	47.5
18.	*Nocardiopsis codii* CT-R113	*Nocardiopsis tropica* JCM 10877	90.90	89.40	38.5
19.	*Nocardiopsis mangrovi* CGMCC 4.7119	*Nocardiopsis sediminis* TBRC 1826	90.88	89.34	38.9
20.	*Nocardiopsis changdeensis* Mg02	*Nocardiopsis flavescens* CGMCC 4.5723	90.11	88.49	36.7
21.	*Nocardiopsis deserti* H13	*Nocardiopsis halotolerans* DSM 44410	89.57	87.15	34.2
22.	*Nocardiopsis alborubida* ATCC 23612	*Nocardiopsis halotolerans* DSM 44410	89.53	87.02	34.1

23.	*Nocardiopsis dassonvillei* subsp. *crassaminis* D1	*Nocardiopsis halotolerans* DSM 44410	89.61	86.95	34.5
24.	*Nocardiopsis ganjiahuensis* DSM 45031	*Nocardiopsis metallicus* JCM 12409	89.57	86.92	34
25.	*Nocardiopsis dassonvillei* subsp. *dassonvillei* DSM 43111	*Nocardiopsis halotolerans* DSM 44410	89.49	86.81	34.1
26.	*Nocardiopsis ganjiahuensis* DSM 45031	*Nocardiopsis valliformis* DSM 45023	89.53	86.69	33.7
27.	*Nocardiopsis exhalans* JCM11759	*Nocardiopsis ganjiahuensis* DSM 45031	89.65	86.67	34.3
28.	*Nocardiopsis halotolerans* DSM 44410	*Nocardiopsis sinuspersici* UTMC 00102	89.02	86.66	21.7
29.	*Nocardiopsis deserti* H13	*Nocardiopsis sinuspersici* UTMC 00102	89.02	86.56	32.2
30.	*Nocardiopsis alborubida* ATCC 23612	*Nocardiopsis arvandica* DSM 45278	88.94	86.39	32.1
31.	*Nocardiopsis ganjiahuensis* DSM 45031	*Nocardiopsis terrae* KCTC 19431	88.85	86.39	32.2
32.	*Nocardiopsis halotolerans* DSM 44410	*Nocardiopsis quinghaiensis* YIM 28A4	88.90	86.39	24.4
33.	*Nocardiopsis akebiae* HDS12	*Nocardiopsis halotolerans* DSM 44410	89.28	86.38	33.5
34.	*Nocardiopsis dassonvillei* subsp. *crassaminis* D1	*Nocardiopsis sinuspersici* UTMC 00102	89.02	86.38	32.5
35.	*Nocardiopsis alborubida* ATCC 23612	*Nocardiopsis sinuspersici* UTMC 00102	88.93	86.37	32.1
36.	*Nocardiopsis dassonvillei* subsp. *dassonvillei* DSM 43111	*Nocardiopsis sinuspersici* UTMC 00102	88.87	86.20	32
37.	*Nocardiopsis deserti* H13	*Nocardiopsis quinghaiensis* YIM 28A4	88.90	86.16	32.3
38.	*Nocardiopsis arvandica* DSM 45278	*Nocardiopsis halotolerans* DSM 44410	88.99	86.07	32.5
39.	*Nocardiopsis alborubida* ATCC 23612	*Nocardiopsis quinghaiensis* YIM 28A4	88.92	85.99	32
40.	*Nocardiopsis dassonvillei* subsp. *crassaminis* D1	*Nocardiopsis quinghaiensis* YIM 28A4	88.98	85.94	32.5
41.	*Nocardiopsis akebiae* HDS12	*Nocardiopsis sinuspersici* UTMC 00102	88.72	85.91	31.6
42.	*Nocardiopsis arvandica* DSM 45278	*Nocardiopsis dassonvillei* subsp. *dassonvillei* DSM 43111	88.87	85.88	32
43.	*Nocardiopsis dassonvillei* subsp. *dassonvillei* DSM 43111	*Nocardiopsis quinghaiensis* YIM 28A4	88.81	85.84	31.9
44.	*Nocardiopsis arvandica* DSM 45278	*Nocardiopsis dassonvillei* subsp. *crassaminis* D1	89.01	85.82	32.5
45.	*Nocardiopsis akebiae* HDS12	*Nocardiopsis arvandica* DSM 45278	88.69	85.81	31.5
46.	*Nocardiopsis arvandica* DSM 45278	*Nocardiopsis deserti* H13	89.02	85.62	32.2
47.	*Nocardiopsis akebiae* HDS12	*Nocardiopsis quinghaiensis* YIM 28A4	88.61	85.61	31.3

48.	*Nocardiopsis metallicus* JCM 12409	*Nocardiopsis terrae* KCTC 19431	88.35	85.50	30.5
49.	*Nocardiopsis exhalans* JCM11759	*Nocardiopsis terrae* KCTC 19431	88.37	85.38	30.5
50.	*Nocardiopsis ganjiahuensis* DSM 45031	*Nocardiopsis prasina* DSM 43845	88.40	85.37	30.9
51.	*Nocardiopsis changdeensis* Mg02	*Nocardiopsis lambiniae* DSM 44743	88.04	85.32	30.4
52.	*Nocardiopsis coralli* HNM0947	*Nocardiopsis litoralis* KCTC 19473	87.55	84.98	30
53.	*Nocardiopsis exhalans* JCM11759	*Nocardiopsis prasina* DSM 43845	88.21	84.93	30.2
54.	*Nocardiopsis metallicus* JCM 12409	*Nocardiopsis prasina* DSM 43845	88.13	84.92	30.1
55.	*Nocardiopsis coralli* HNM0947	*Nocardiopsis kunsanensis* DSM 44524	87.56	84.90	29.9
56.	*Nocardiopsis terrae* KCTC 19431	*Nocardiopsis valliformis* DSM 45023	88.28	84.57	30.3
57.	*Nocardiopsis flavescens* CGMCC 4.5723	*Nocardiopsis lambiniae* DSM 44743	87.58	84.50	29.1
58.	*Nocardiopsis prasina* DSM 43845	*Nocardiopsis valliformis* DSM 45023	88.10	84.19	29.8
59.	*Nocardiopsis prasina* DSM 43845	*Nocardiopsis terrae* KCTC 19431	87.69	84.16	29.0
60.	*Nocardiopsis codii* CT-R113	*Nocardiopsis dassonvillei* subsp. *dassonvillei* DSM 43111	87.01	83.27	27.5
61.	*Nocardiopsis dassonvillei* subsp. *crassaminis* D1	*Nocardiopsis tropica* JCM 10877	87.19	83.11	28
62.	*Nocardiopsis codii* CT-R113	*Nocardiopsis sinuspersici* UTMC 00102	86.93	83.08	27.5
63.	*Nocardiopsis dassonvillei* subsp. *dassonvillei* DSM 43111	*Nocardiopsis tropica* JCM 10877	87.07	83.08	27.3
64.	*Nocardiopsis deserti* H13	*Nocardiopsis tropica* JCM 10877	87.20	83.04	27.7
65.	*Nocardiopsis codii* CT-R113	*Nocardiopsis dassonvillei* subsp. *crassaminis* D1	87.16	83.03	27.9
66.	*Nocardiopsis alborubida* ATCC 23612	*Nocardiopsis tropica* JCM 10877	87.19	83.00	27.6
67.	*Nocardiopsis codii* CT-R113	*Nocardiopsis quinghaiensis* YIM 28A4	87.00	82.96	27.4
68.	*Nocardiopsis akebiae* HDS12	*Nocardiopsis tropica* JCM 10877	87.04	82.87	27.3
69.	*Nocardiopsis codii* CT-R113	*Nocardiopsis deserti* H13	87.10	82.70	27.6
70.	*Nocardiopsis alborubida* ATCC 23612	*Nocardiopsis codii* CT-R113	87.07	82.63	27.5
71.	*Nocardiopsis arvandica* DSM 45278	*Nocardiopsis codii* CT-R113	86.95	82.50	27.4
72.	*Nocardiopsis arvandica* DSM 45278	*Nocardiopsis tropica* JCM 10877	86.96	82.47	27.2

73.	*Nocardiopsis akebiae* HDS12	*Nocardiopsis codii* CT-R113	86.95	82.46	27
74.	*Nocardiopsis sinuspersici* UTMC 00102	*Nocardiopsis tropica* JCM 10877	86.96	82.44	27.1
75.	*Nocardiopsis alkaliphila* YIM 80379	*Nocardiopsis listeri* NBRC 13360	86.54	82.36	26.3
76.	*Nocardiopsis codii* CT-R113	*Nocardiopsis halotolerans* DSM 44410	86.87	82.34	27.2
77.	*Nocardiopsis quinghaiensis* YIM 28A4	*Nocardiopsis tropica* JCM 10877	86.94	82.34	27.0
78.	*Nocardiopsis halotolerans* DSM 44410	*Nocardiopsis tropica* JCM 10877	86.79	82.09	21.7
79.	*Nocardiopsis aegyptia* DSM 44442	*Nocardiopsis arvandica* DSM 45278	86.47	81.25	25.5
80.	*Nocardiopsis alborubida* ATCC 23612	*Nocardiopsis algeriensis* CECT 8712	86.33	81.23	25.3
81.	*Nocardiopsis aegyptia* DSM 44442	*Nocardiopsis sinuspersici* UTMC 00102	86.48	81.22	25.4
82.	*Nocardiopsis akebiae* HDS12	*Nocardiopsis algeriensis* CECT 8712	86.26	81.20	25.2
83.	*Nocardiopsis ganjiahuensis* DSM 45031	*Nocardiopsis listeri* NBRC 13360	86.24	81.17	25.1
84.	*Nocardiopsis sediminis* TBRC 1826	*Nocardiopsis trehalosi* NBRC 14201	86.00	81.14	24.4
85.	*Nocardiopsis mangrovi* CGMCC 4.7119	*Nocardiopsis trehalosi* NBRC 14201	85.89	81.10	24.5
86.	*Nocardiopsis aegyptia* DSM 44442	*Nocardiopsis quinghaiensis* YIM 28A4	86.26	81.08	25
87.	*Nocardiopsis exhalans* JCM11759	*Nocardiopsis listeri* NBRC 13360	86.29	81.05	25.1
88.	*Nocardiopsis alba* DSM 43377	*Nocardiopsis terrae* KCTC 19431	86.39	81.01	25
89.	*Nocardiopsis aegyptia* DSM 44442	*Nocardiopsis dassonvillei* subsp. *dassonvillei* DSM 43111	86.45	80.98	25.3
90.	*Nocardiopsis listeri* NBRC 13360	*Nocardiopsis terrae* KCTC 19431	86.26	80.97	24.7
91.	*Nocardiopsis alba* DSM 43377	*Nocardiopsis alkaliphila* YIM 80379	86.15	80.95	24.9
92.	*Nocardiopsis alba* DSM 43377	*Nocardiopsis listeri* NBRC 13360	86.23	80.89	24.9
93.	*Nocardiopsis algeriensis* CECT 8712	*Nocardiopsis sinuspersici* UTMC 00102	86.20	80.80	24.9
94.	*Nocardiopsis aegyptia* DSM 44442	*Nocardiopsis akebiae* HDS12	86.43	80.79	25.2
95.	*Nocardiopsis aegyptia* DSM 44442	*Nocardiopsis dassonvillei* subsp. *crassaminis* D1	86.51	80.77	25.8
96.	*Nocardiopsis algeriensis* CECT 8712	*Nocardiopsis dassonvillei* subsp. *dassonvillei* DSM 43111	86.39	80.77	25.4
97.	*Nocardiopsis changdeensis* Mg02	*Nocardiopsis dassonvillei* subsp. *dassonvillei* DSM 43111	86.29	80.74	24.7

98.	*Nocardiopsis algeriensis* CECT 8712	*Nocardiopsis arvandica* DSM 45278	86.21	80.72	24.9
99.	*Nocardiopsis algeriensis* CECT 8712	*Nocardiopsis dassonvillei* subsp. *crassaminis* D1	86.51	80.61	25.8
100.	*Nocardiopsis alba* DSM 43377	*Nocardiopsis exhalans* JCM11759	86.50	80.59	25.1
101.	*Nocardiopsis alba* DSM 43377	*Nocardiopsis prasina* DSM 43845	86.44	80.59	24.7
102.	*Nocardiopsis dassonvillei* subsp. *crassaminis* D1	*Nocardiopsis flavescens* CGMCC 4.5723	86.23	80.54	24.8
103.	*Nocardiopsis algeriensis* CECT 8712	*Nocardiopsis quinghaiensis* YIM 28A4	86.10	80.52	24.7
104.	*Nocardiopsis ganjiahuensis* DSM 45031	*Nocardiopsis sinuspersici* UTMC 00102	86.20	80.50	24.5
105.	*Nocardiopsis alkaliphila* YIM 80379	*Nocardiopsis terrae* KCTC 19431	86.01	80.49	24.5
106.	*Nocardiopsis changdeensis* Mg02	*Nocardiopsis sinuspersici* UTMC 00102	86.19	80.48	24.2
107.	*Nocardiopsis listeri* NBRC 13360	*Nocardiopsis metallicus* JCM 12409	86.29	80.47	25
108.	*Nocardiopsis aegyptia* DSM 44442	*Nocardiopsis deserti* H13	86.46	80.45	25.4
109.	*Nocardiopsis dassonvillei* subsp. *dassonvillei* DSM 43111	*Nocardiopsis flavescens* CGMCC 4.5723	86.17	80.43	24.2
110.	*Nocardiopsis changdeensis* Mg02	*Nocardiopsis dassonvillei* subsp. *crassaminis* D1	86.39	80.41	25.2
111.	*Nocardiopsis aegyptia* DSM 44442	*Nocardiopsis alborubida* ATCC 23612	86.49	80.39	25.3
112.	*Nocardiopsis alba* DSM 43377	*Nocardiopsis ganjiahuensis* DSM 45031	86.47	80.39	25.3
113.	*Nocardiopsis alba* DSM 43377	*Nocardiopsis metallicus* JCM 12409	86.47	80.39	25.1
114.	*Nocardiopsis alborubida* ATCC 23612	*Nocardiopsis changdeensis* Mg02	86.23	80.37	24.7
115.	*Nocardiopsis alborubida* ATCC 23612	*Nocardiopsis flavescens* CGMCC 4.5723	86.17	80.36	24.4
116.	*Nocardiopsis deserti* H13	*Nocardiopsis flavescens* CGMCC 4.5723	86.19	80.33	24.5
117.	*Nocardiopsis sinuspersici* UTMC 00102	*Nocardiopsis terrae* KCTC 19431	86.23	80.29	24.3
118.	*Nocardiopsis aegyptia* DSM 44442	*Nocardiopsis halotolerans* DSM 44410	86.22	80.28	25.1
119.	*Nocardiopsis algeriensis* CECT 8712	*Nocardiopsis deserti* H13	86.34	80.28	25.5
120.	*Nocardiopsis akebiae* HDS12	*Nocardiopsis flavescens* CGMCC 4.5723	86.16	80.27	24.2
121.	*Nocardiopsis exhalans* JCM11759	*Nocardiopsis sinuspersici* UTMC 00102	86.18	80.26	24.6
122.	*Nocardiopsis listeri* NBRC 13360	*Nocardiopsis valliformis* DSM 45023	86.22	80.26	24.9

123.	*Nocardiopsis akebiae* HDS12	*Nocardiopsis changdeensis* Mg02	86.34	80.25	24.7
124.	*Nocardiopsis dassonvillei* subsp. *crassaminis* D1	*Nocardiopsis terrae* KCTC 19431	86.33	80.24	25
125.	*Nocardiopsis exhalans* JCM11759	*Nocardiopsis quinghaiensis* YIM 28A4	86.16	80.24	24.4
126.	*Nocardiopsis listeri* NBRC 13360	*Nocardiopsis prasina* DSM 43845	86.00	80.24	24.2
127.	*Nocardiopsis quinghaiensis* YIM 28A4	*Nocardiopsis terrae* KCTC 19431	86.16	80.23	24.1
128.	*Nocardiopsis changdeensis* Mg02	*Nocardiopsis quinghaiensis* YIM 28A4	85.99	80.22	23.9
129.	*Nocardiopsis aegyptia* DSM 44442	*Nocardiopsis tropica* JCM 10877	86.14	80.21	24.8
130.	*Nocardiopsis dassonvillei* subsp. *crassaminis* D1	*Nocardiopsis lambiniae* DSM 44743	86.18	80.21	24.8
131.	*Nocardiopsis algeriensis* CECT 8712	*Nocardiopsis changdeensis* Mg02	86.37	80.20	24.6
132.	*Nocardiopsis metallicus* JCM 12409	*Nocardiopsis sinuspersici* UTMC 00102	86.08	80.19	24.3
133.	*Nocardiopsis alkaliphila* YIM 80379	*Nocardiopsis exhalans* JCM11759	86.12	80.18	24.9
134.	*Nocardiopsis deserti* H13	*Nocardiopsis terrae* KCTC 19431	86.27	80.18	24.6
135.	*Nocardiopsis algeriensis* CECT 8712	*Nocardiopsis flavescens* CGMCC 4.5723	86.11	80.17	24.1
136.	*Nocardiopsis alkaliphila* YIM 80379	*Nocardiopsis metallicus* JCM 12409	86.07	80.17	24.9
137.	*Nocardiopsis arvandica* DSM 45278	*Nocardiopsis terrae* KCTC 19431	86.25	80.17	24.3
138.	*Nocardiopsis ganjiahuensis* DSM 45031	*Nocardiopsis quinghaiensis* YIM 28A4	86.14	80.17	24.2
139.	*Nocardiopsis changdeensis* Mg02	*Nocardiopsis deserti* H13	86.26	80.16	24.7
140.	*Nocardiopsis codii* CT-R113	*Nocardiopsis flavescens* CGMCC 4.5723	85.76	80.15	23.6
141.	*Nocardiopsis alkaliphila* YIM 80379	*Nocardiopsis ganjiahuensis* DSM 45031	86.10	80.13	24.9
142.	*Nocardiopsis lambiniae* DSM 44743	*Nocardiopsis sinuspersici* UTMC 00102	85.82	80.13	24.1
143.	*Nocardiopsis alborubida* ATCC 23612	*Nocardiopsis lambiniae* DSM 44743	86.06	80.11	24.5
144.	*Nocardiopsis alkaliphila* YIM 80379	*Nocardiopsis valliformis* DSM 45023	86.13	80.11	24.8
145.	*Nocardiopsis alborubida* ATCC 23612	*Nocardiopsis terrae* KCTC 19431	86.21	80.09	24.5
146.	*Nocardiopsis flavescens* CGMCC 4.5723	*Nocardiopsis sinuspersici* UTMC 00102	85.96	80.09	23.8
147.	*Nocardiopsis alba* DSM 43377	*Nocardiopsis valliformis* DSM 45023	86.40	80.08	24.9

148.	*Nocardiopsis metallicus* JCM 12409	*Nocardiopsis quinghaiensis* YIM 28A4	86.12	80.06	24.3
149.	*Nocardiopsis arvandica* DSM 45278	*Nocardiopsis flavescens* CGMCC 4.5723	85.95	80.05	23.9
150.	*Nocardiopsis dassonvillei* subsp. *dassonvillei* DSM 43111	*Nocardiopsis lambiniae* DSM 44743	86.02	80.04	24.4
151.	*Nocardiopsis arvandica* DSM 45278	*Nocardiopsis changdeensis* Mg02	86.12	80.03	24.2
152.	*Nocardiopsis algeriensis* CECT 8712	*Nocardiopsis halotolerans* DSM 44410	86.15	80.02	25
153.	*Nocardiopsis dassonvillei* subsp. *dassonvillei* DSM 43111	*Nocardiopsis terrae* KCTC 19431	86.20	80.01	24.5
154.	*Nocardiopsis deserti* H13	*Nocardiopsis lambiniae* DSM 44743	86.02	79.99	24.3
155.	*Nocardiopsis algeriensis* CECT 8712	*Nocardiopsis lambiniae* DSM 44743	86.10	79.95	24.2
156.	*Nocardiopsis aegyptia* DSM 44442	*Nocardiopsis codii* CT-R113	86.12	79.93	24.8
157.	*Nocardiopsis akebiae* HDS12	*Nocardiopsis terrae* KCTC 19431	86.17	79.92	24.4
158.	*Nocardiopsis codii* CT-R113	*Nocardiopsis terrae* KCTC 19431	86.03	79.91	24.2
159.	*Nocardiopsis flavescens* CGMCC 4.5723	*Nocardiopsis quinghaiensis* YIM 28A4	85.86	79.91	23.5
160.	*Nocardiopsis halotolerans* DSM 44410	*Nocardiopsis terrae* KCTC 19431	86.07	79.91	21.6
161.	*Nocardiopsis ganjiahuensis* DSM 45031	*Nocardiopsis tropica* JCM 10877	86.02	79.90	24
162.	*Nocardiopsis akebiae* HDS12	*Nocardiopsis lambiniae* DSM 44743	86.09	79.89	24.3
163.	*Nocardiopsis lambiniae* DSM 44743	*Nocardiopsis quinghaiensis* YIM 28A4	85.82	79.87	23.7
164.	*Nocardiopsis arvandica* DSM 45278	*Nocardiopsis lambiniae* DSM 44743	85.84	79.83	24.1
165.	*Nocardiopsis prasina* DSM 43845	*Nocardiopsis sinuspersici* UTMC 00102	85.90	79.83	24.2
166.	*Nocardiopsis baichengensis* YIM 90130	*Nocardiopsis potens* DSM 45234	85.74	79.82	23.6
167.	*Nocardiopsis changdeensis* Mg02	*Nocardiopsis tropica* JCM 10877	85.99	79.82	24
168.	*Nocardiopsis dassonvillei* subsp. *crassaminis* D1	*Nocardiopsis prasina* DSM 43845	86.20	79.82	24.9
169.	*Nocardiopsis alborubida* ATCC 23612	*Nocardiopsis metallicus* JCM 12409	86.53	79.81	25.3
170.	*Nocardiopsis aegyptia* DSM 44442	*Nocardiopsis algeriensis* CECT 8712	85.73	79.79	23.8
171.	*Nocardiopsis deserti* H13	*Nocardiopsis prasina* DSM 43845	86.11	79.77	24.6
172.	*Nocardiopsis algeriensis* CECT 8712	*Nocardiopsis tropica* JCM 10877	85.87	79.73	24.4

173.	*Nocardiopsis ganjiahuensis* DSM 45031	*Nocardiopsis halotolerans* DSM 44410	86.09	79.73	24.5
174.	*Nocardiopsis codii* CT-R113	*Nocardiopsis lambiniae* DSM 44743	85.74	79.72	23.7
175.	*Nocardiopsis dassonvillei* subsp. *dassonvillei* DSM 43111	*Nocardiopsis prasina* DSM 43845	86.15	79.72	24.4
176.	*Nocardiopsis flavescens* CGMCC 4.5723	*Nocardiopsis tropica* JCM 10877	85.87	79.72	23.8
177.	*Nocardiopsis prasina* DSM 43845	*Nocardiopsis quinghaiensis* YIM 28A4	85.93	79.72	23.9
178.	*Nocardiopsis dassonvillei* subsp. *crassaminis* D1	*Nocardiopsis ganjiahuensis* DSM 45031	86.29	79.71	25
179.	*Nocardiopsis changdeensis* Mg02	*Nocardiopsis codii* CT-R113	85.88	79.7	23.9
180.	*Nocardiopsis alborubida* ATCC 23612	*Nocardiopsis exhalans* JCM11759	86.47	79.69	25.2
181.	*Nocardiopsis alborubida* ATCC 23612	*Nocardiopsis prasina* DSM 43845	86.08	79.69	24.5
182.	*Nocardiopsis akebiae* HDS12	*Nocardiopsis prasina* DSM 43845	85.99	79.68	24.3
183.	*Nocardiopsis changdeensis* Mg02	*Nocardiopsis halotolerans* DSM 44410	86.07	79.68	24.2
184.	*Nocardiopsis codii* CT-R113	*Nocardiopsis prasina* DSM 43845	85.87	79.66	24
185.	*Nocardiopsis dassonvillei* subsp. *crassaminis* D1	*Nocardiopsis exhalans* JCM11759	86.32	79.64	25.3
186.	*Nocardiopsis alkaliphila* YIM 80379	*Nocardiopsis prasina* DSM 43845	85.88	79.63	24
187.	*Nocardiopsis halotolerans* DSM 44410	*Nocardiopsis lambiniae* DSM 44743	85.86	79.62	22.5
188.	*Nocardiopsis dassonvillei* subsp. *dassonvillei* DSM 43111	*Nocardiopsis ganjiahuensis* DSM 45031	86.17	79.61	24.5
189.	*Nocardiopsis terrae* KCTC 19431	*Nocardiopsis tropica* JCM 10877	85.95	79.60	24
190.	*Nocardiopsis aegyptia* DSM 44442	*Nocardiopsis terrae* KCTC 19431	86.12	79.59	23.8
191.	*Nocardiopsis dassonvillei* subsp. *crassaminis* D1	*Nocardiopsis metallicus* JCM 12409	86.33	79.59	25.2
192.	*Nocardiopsis alborubida* ATCC 23612	*Nocardiopsis ganjiahuensis* DSM 45031	86.20	79.57	24.7
193.	*Nocardiopsis prasina* DSM 43845	*Nocardiopsis tropica* JCM 10877	85.81	79.57	23.8
194.	*Nocardiopsis arvandica* DSM 45278	*Nocardiopsis ganjiahuensis* DSM 45031	86.18	79.56	24.6
195.	*Nocardiopsis arvandica* DSM 45278	*Nocardiopsis exhalans* JCM11759	86.17	79.52	24.6
196.	*Nocardiopsis dassonvillei* subsp. *dassonvillei* DSM 43111	*Nocardiopsis exhalans* JCM11759	86.22	79.52	24.8
197.	*Nocardiopsis metallicus* JCM 12409	*Nocardiopsis tropica* JCM 10877	85.99	79.52	24

198.	*Nocardiopsis deserti* H13	*Nocardiopsis ganjiahuensis* DSM 45031	86.24	79.51	24.6
199.	*Nocardiopsis exhalans* JCM11759	*Nocardiopsis halotolerans* DSM 44410	86.05	79.47	24.6
200.	*Nocardiopsis arvandica* DSM 45278	*Nocardiopsis prasina* DSM 43845	85.92	79.45	24.1
201.	*Nocardiopsis dassonvillei* subsp. *dassonvillei* DSM 43111	*Nocardiopsis metallicus* JCM 12409	86.24	79.45	24.6
202.	*Nocardiopsis akebiae* HDS12	*Nocardiopsis ganjiahuensis* DSM 45031	86.20	79.43	24.3
203.	*Nocardiopsis codii* CT-R113	*Nocardiopsis ganjiahuensis* DSM 45031	86.05	79.43	24.2
204.	*Nocardiopsis flavescens* CGMCC 4.5723	*Nocardiopsis halotolerans* DSM 44410	85.80	79.41	23.8
205.	*Nocardiopsis algeriensis* CECT 8712	*Nocardiopsis codii* CT-R113	85.88	79.40	24.4
206.	*Nocardiopsis arvandica* DSM 45278	*Nocardiopsis valliformis* DSM 45023	86.04	79.40	24.5
207.	*Nocardiopsis dassonvillei* subsp. *crassaminis* D1	*Nocardiopsis valliformis* DSM 45023	86.28	79.40	25
208.	*Nocardiopsis deserti* H13	*Nocardiopsis exhalans* JCM11759	86.20	79.40	24.8
209.	*Nocardiopsis exhalans* JCM11759	*Nocardiopsis tropica* JCM 10877	86.01	79.40	24.1
210.	*Nocardiopsis lambiniae* DSM 44743	*Nocardiopsis tropica* JCM 10877	85.78	79.39	23.7
211.	*Nocardiopsis deserti* H13	*Nocardiopsis metallicus* JCM 12409	86.19	79.37	24.8
212.	*Nocardiopsis arvandica* DSM 45278	*Nocardiopsis metallicus* JCM 12409	86.08	79.36	24.4
213.	*Nocardiopsis akebiae* HDS12	*Nocardiopsis exhalans* JCM11759	86.23	79.33	24.6
214.	*Nocardiopsis akebiae* HDS12	*Nocardiopsis metallicus* JCM 12409	86.22	79.29	24.5
215.	*Nocardiopsis sinuspersici* UTMC 00102	*Nocardiopsis valliformis* DSM 45023	86.01	79.27	24.4
216.	*Nocardiopsis dassonvillei* subsp. *dassonvillei* DSM 43111	*Nocardiopsis valliformis* DSM 45023	86.17	79.26	24.6
217.	*Nocardiopsis deserti* H13	*Nocardiopsis valliformis* DSM 45023	86.13	79.25	24.6
218.	*Nocardiopsis halotolerans* DSM 44410	*Nocardiopsis prasina* DSM 43845	86.01	79.22	21.9
219.	*Nocardiopsis codii* CT-R113	*Nocardiopsis exhalans* JCM11759	86.00	79.20	24.2
220.	*Nocardiopsis halotolerans* DSM 44410	*Nocardiopsis metallicus* JCM 12409	86.08	79.20	22
221.	*Nocardiopsis alborubida* ATCC 23612	*Nocardiopsis valliformis* DSM 45023	86.13	79.18	24.6
222.	*Nocardiopsis aegyptia* DSM 44442	*Nocardiopsis changdeensis* Mg02	85.91	79.17	23.5

223.	*Nocardiopsis codii* CT-R113	*Nocardiopsis metallicus* JCM 12409	86.02	79.14	24.2
224.	*Nocardiopsis codii* CT-R113	*Nocardiopsis valliformis* DSM 45023	85.95	79.13	24.1
225.	*Nocardiopsis akebiae* HDS12	*Nocardiopsis valliformis* DSM 45023	86.18	79.12	24.4
226.	*Nocardiopsis quinghaiensis* YIM 28A4	*Nocardiopsis valliformis* DSM 45023	86.02	79.11	24.1
227.	*Nocardiopsis aegyptia* DSM 44442	*Nocardiopsis prasina* DSM 43845	85.88	79.02	23.5
228.	*Nocardiopsis aegyptia* DSM 44442	*Nocardiopsis exhalans* JCM11759	85.97	79.00	24
229.	*Nocardiopsis alba* DSM 43377	*Nocardiopsis dassonvillei* subsp. *dassonvillei* DSM 43111	85.89	78.98	23.7
230.	*Nocardiopsis halotolerans* DSM 44410	*Nocardiopsis valliformis* DSM 45023	86.04	78.98	26.8
231.	*Nocardiopsis tropica* JCM 10877	*Nocardiopsis valliformis* DSM 45023	85.90	78.94	23.9
232.	*Nocardiopsis ganjiahuensis* DSM 45031	*Nocardiopsis lambiniae* DSM 44743	85.77	78.93	23
233.	*Nocardiopsis aegyptia* DSM 44442	*Nocardiopsis lambiniae* DSM 44743	85.72	78.91	23.4
234.	*Nocardiopsis alba* DSM 43377	*Nocardiopsis sinuspersici* UTMC 00102	85.71	78.91	23.4
235.	*Nocardiopsis aegyptia* DSM 44442	*Nocardiopsis metallicus* JCM 12409	85.97	78.90	24
236.	*Nocardiopsis aegyptia* DSM 44442	*Nocardiopsis flavescens* CGMCC 4.5723	85.64	78.89	23.2
237.	*Nocardiopsis listeri* NBRC 13360	*Nocardiopsis sinuspersici* UTMC 00102	85.56	78.89	23.1
238.	*Nocardiopsis aegyptia* DSM 44442	*Nocardiopsis ganjiahuensis* DSM 45031	86.09	78.88	24.2
239.	*Nocardiopsis alba* DSM 43377	*Nocardiopsis arvandica* DSM 45278	85.73	78.86	23.4
240.	*Nocardiopsis lambiniae* DSM 44743	*Nocardiopsis terrae* KCTC 19431	85.70	78.86	22.7
241.	*Nocardiopsis changdeensis* Mg02	*Nocardiopsis terrae* KCTC 19431	85.63	78.84	22.7
242.	*Nocardiopsis dassonvillei* subsp. *crassaminis* D1	*Nocardiopsis listeri* NBRC 13360	85.60	78.84	23.7
243.	*Nocardiopsis aegyptia* DSM 44442	*Nocardiopsis valliformis* DSM 45023	85.92	78.79	23.9
244.	*Nocardiopsis exhalans* JCM11759	*Nocardiopsis flavescens* CGMCC 4.5723	85.61	78.79	23
245.	*Nocardiopsis exhalans* JCM11759	*Nocardiopsis lambiniae* DSM 44743	85.68	78.77	23.1
246.	*Nocardiopsis listeri* NBRC 13360	*Nocardiopsis quinghaiensis* YIM 28A4	85.61	78.76	22.8
247.	*Nocardiopsis alborubida* ATCC 23612	*Nocardiopsis listeri* NBRC 13360	85.59	78.72	23.2

248.	*Nocardiopsis akebiae* HDS12	*Nocardiopsis alba* DSM 43377	85.84	78.67	23.5
249.	*Nocardiopsis alba* DSM 43377	*Nocardiopsis quinghaiensis* YIM 28A4	85.70	78.67	23.1
250.	*Nocardiopsis deserti* H13	*Nocardiopsis listeri* NBRC 13360	85.59	78.66	23.3
251.	*Nocardiopsis dassonvillei* subsp. *dassonvillei* DSM 43111	*Nocardiopsis listeri* NBRC 13360	85.55	78.64	23.3
252.	*Nocardiopsis alba* DSM 43377	*Nocardiopsis dassonvillei* subsp. *crassaminis* D1	85.89	78.63	24.1
253.	*Nocardiopsis akebiae* HDS12	*Nocardiopsis listeri* NBRC 13360	85.55	78.61	23.2
254.	*Nocardiopsis algeriensis* CECT 8712	*Nocardiopsis terrae* KCTC 19431	85.55	78.56	23.1
255.	*Nocardiopsis arvandica* DSM 45278	*Nocardiopsis listeri* NBRC 13360	85.57	78.56	23.2
256.	*Nocardiopsis alkaliphila* YIM 80379	*Nocardiopsis arvandica* DSM 45278	85.41	78.53	23.1
257.	*Nocardiopsis changdeensis* Mg02	*Nocardiopsis prasina* DSM 43845	85.73	78.5	22.9
258.	*Nocardiopsis flavescens* CGMCC 4.5723	*Nocardiopsis terrae* KCTC 19431	85.50	78.50	22.6
259.	*Nocardiopsis changdeensis* Mg02	*Nocardiopsis ganjiahuensis* DSM 45031	85.8	78.47	23.1
260.	*Nocardiopsis alba* DSM 43377	*Nocardiopsis alborubida* ATCC 23612	85.80	78.44	23.6
261.	*Nocardiopsis alba* DSM 43377	*Nocardiopsis deserti* H13	85.79	78.44	23.7
262.	*Nocardiopsis codii* CT-R113	*Nocardiopsis listeri* NBRC 13360	85.47	78.39	23
263.	*Nocardiopsis alba* DSM 43377	*Nocardiopsis tropica* JCM 10877	85.60	78.37	22.9
264.	*Nocardiopsis coralli* HNM0947	*Nocardiopsis dassonvillei* subsp. *dassonvillei* DSM 43111	85.47	78.33	23.2
265.	*Nocardiopsis flavescens* CGMCC 4.5723	*Nocardiopsis prasina* DSM 43845	85.57	78.33	22.7
266.	*Nocardiopsis alkaliphila* YIM 80379	*Nocardiopsis dassonvillei* subsp. *crassaminis* D1	85.54	78.32	23.5
267.	*Nocardiopsis coralli* HNM0947	*Nocardiopsis sinuspersici* UTMC 00102	85.40	78.32	22.9
268.	*Nocardiopsis halotolerans* DSM 44410	*Nocardiopsis listeri* NBRC 13360	85.54	78.29	23.9
269.	*Nocardiopsis alborubida* ATCC 23612	*Nocardiopsis alkaliphila* YIM 80379	85.42	78.28	23
270.	*Nocardiopsis alkaliphila* YIM 80379	*Nocardiopsis dassonvillei* subsp. *dassonvillei* DSM 43111	85.49	78.25	23.3
271.	*Nocardiopsis alkaliphila* YIM 80379	*Nocardiopsis sinuspersici* UTMC 00102	85.41	78.25	22.9
272.	*Nocardiopsis akebiae* HDS12	*Nocardiopsis alkaliphila* YIM 80379	85.51	78.24	23.2

273.	*Nocardiopsis alba* DSM 43377	*Nocardiopsis halotolerans* DSM 44410	85.78	78.24	23.5
274.	*Nocardiopsis lambiniae* DSM 44743	*Nocardiopsis prasina* DSM 43845	85.63	78.23	22.6
275.	*Nocardiopsis listeri* NBRC 13360	*Nocardiopsis tropica* JCM 10877	85.46	78.22	22.8
276.	*Nocardiopsis alba* DSM 43377	*Nocardiopsis algeriensis* CECT 8712	85.60	78.20	22.4
277.	*Nocardiopsis coralli* HNM0947	*Nocardiopsis dassonvillei* subsp. *crassaminis* D1	85.52	78.20	23.6
278.	*Nocardiopsis changdeensis* Mg02	*Nocardiopsis metallicus* JCM 12409	85.75	78.19	23.1
279.	*Nocardiopsis coralli* HNM0947	*Nocardiopsis quinghaiensis* YIM 28A4	85.35	78.18	22.6
280.	*Nocardiopsis changdeensis* Mg02	*Nocardiopsis valliformis* DSM 45023	85.65	78.16	23.1
281.	*Nocardiopsis alba* DSM 43377	*Nocardiopsis codii* CT-R113	85.70	78.15	23
282.	*Nocardiopsis flavescens* CGMCC 4.5723	*Nocardiopsis ganjiahuensis* DSM 45031	85.64	78.15	22.9
283.	*Nocardiopsis flavescens* CGMCC 4.5723	*Nocardiopsis metallicus* JCM 12409	85.65	78.15	23
284.	*Nocardiopsis lambiniae* DSM 44743	*Nocardiopsis metallicus* JCM 12409	85.62	78.15	22.9
285.	*Nocardiopsis algeriensis* CECT 8712	*Nocardiopsis ganjiahuensis* DSM 45031	85.72	78.14	23.6
286.	*Nocardiopsis algeriensis* CECT 8712	*Nocardiopsis prasina* DSM 43845	85.57	78.14	23.1
287.	*Nocardiopsis dassonvillei* subsp. *crassaminis* D1	*Nocardiopsis litoralis* KCTC 19473	85.29	78.13	23.1
288.	*Nocardiopsis potens* DSM 45234	*Nocardiopsis trehalosi* NBRC 14201	85.10	78.13	22.1
289.	*Nocardiopsis alkaliphila* YIM 80379	*Nocardiopsis quinghaiensis* YIM 28A4	85.36	78.12	22.7
290.	*Nocardiopsis changdeensis* Mg02	*Nocardiopsis exhalans* JCM11759	85.75	78.1	23.2
291.	*Nocardiopsis aegyptia* DSM 44442	*Nocardiopsis alba* DSM 43377	85.7	78.08	23.1
292.	*Nocardiopsis algeriensis* CECT 8712	*Nocardiopsis exhalans* JCM11759	85.72	78.08	23.6
293.	*Nocardiopsis algeriensis* CECT 8712	*Nocardiopsis valliformis* DSM 45023	85.59	78.08	23.4
294.	*Nocardiopsis algeriensis* CECT 8712	*Nocardiopsis metallicus* JCM 12409	85.73	78.07	23.6
295.	*Nocardiopsis lambiniae* DSM 44743	*Nocardiopsis valliformis* DSM 45023	85.71	78.02	23.2
296.	*Nocardiopsis rhodophaea* JCM 15313	*Nocardiopsis trehalosi* NBRC 14201	85.24	78.02	22.5
297.	*Nocardiopsis aegyptia* DSM 44442	*Nocardiopsis listeri* NBRC 13360	85.45	77.99	22.7

298.	*Nocardiopsis alba* DSM 43377	*Nocardiopsis changdeensis* Mg02	85.65	77.98	22.5
299.	*Nocardiopsis alkaliphila* YIM 80379	*Nocardiopsis deserti* H13	85.41	77.98	23.1
300.	*Nocardiopsis deserti* H13	*Nocardiopsis litoralis* KCTC 19473	85.12	77.95	22.6
301.	*Nocardiopsis akebiae* HDS12	*Nocardiopsis litoralis* KCTC 19473	85.12	77.93	22.6
302.	*Nocardiopsis alkaliphila* YIM 80379	*Nocardiopsis halotolerans* DSM 44410	85.52	77.92	23
303.	*Nocardiopsis dassonvillei* subsp. *crassaminis* D1	*Nocardiopsis kunsanensis* DSM 44524	85.27	77.91	23
304.	*Nocardiopsis coralli* HNM0947	*Nocardiopsis halotolerans* DSM 44410	85.47	77.86	23.1
305.	*Nocardiopsis dassonvillei* subsp. *dassonvillei* DSM 43111	*Nocardiopsis litoralis* KCTC 19473	85.29	77.86	22.6
306.	*Nocardiopsis alba* DSM 43377	*Nocardiopsis flavescens* CGMCC 4.5723	85.44	77.85	22.1
307.	*Nocardiopsis coralli* HNM0947	*Nocardiopsis deserti* H13	85.42	77.85	23.1
308.	*Nocardiopsis coralli* HNM0947	*Nocardiopsis terrae* KCTC 19431	85.34	77.85	22.3
309.	*Nocardiopsis flavescens* CGMCC 4.5723	*Nocardiopsis valliformis* DSM 45023	85.55	77.84	22.8
310.	*Nocardiopsis litoralis* KCTC 19473	*Nocardiopsis sinuspersici* UTMC 00102	85.13	77.84	22.3
311.	*Nocardiopsis akebiae* HDS12	*Nocardiopsis coralli* HNM0947	85.48	77.82	23
312.	*Nocardiopsis alborubida* ATCC 23612	*Nocardiopsis litoralis* KCTC 19473	85.13	77.81	22.7
313.	*Nocardiopsis changdeensis* Mg02	*Nocardiopsis listeri* NBRC 13360	85.32	77.8	22.1
314.	*Nocardiopsis alborubida* ATCC 23612	*Nocardiopsis coralli* HNM0947	85.50	77.78	23.2
315.	*Nocardiopsis arvandica* DSM 45278	*Nocardiopsis litoralis* KCTC 19473	85.07	77.78	22.3
316.	*Nocardiopsis dassonvillei* subsp. *dassonvillei* DSM 43111	*Nocardiopsis kunsanensis* DSM 44524	85.26	77.73	22.5
317.	*Nocardiopsis kunsanensis* DSM 44524	*Nocardiopsis sinuspersici* UTMC 00102	85.12	77.73	22.2
318.	*Nocardiopsis alba* DSM 43377	*Nocardiopsis lambiniae* DSM 44743	85.57	77.72	22.4
319.	*Nocardiopsis coralli* HNM0947	*Nocardiopsis prasina* DSM 43845	85.14	77.72	22.3
320.	*Nocardiopsis akebiae* HDS12	*Nocardiopsis kunsanensis* DSM 44524	85.11	77.70	22.5
321.	*Nocardiopsis halotolerans* DSM 44410	*Nocardiopsis litoralis* KCTC 19473	85.40	77.69	23.2
322.	*Nocardiopsis coralli* HNM0947	*Nocardiopsis tropica* JCM 10877	85.21	77.68	22.5

323.	*Nocardiopsis exhalans* JCM11759	*Nocardiopsis litoralis* KCTC 19473	85.14	77.68	22.5
324.	*Nocardiopsis alborubida* ATCC 23612	*Nocardiopsis kunsanensis* DSM 44524	85.13	77.67	22.7
325.	*Nocardiopsis arvandica* DSM 45278	*Nocardiopsis coralli* HNM0947	85.45	77.67	22.9
326.	*Nocardiopsis alkaliphila* YIM 80379	*Nocardiopsis codii* CT-R113	85.33	77.66	22.5
327.	*Nocardiopsis alkaliphila* YIM 80379	*Nocardiopsis tropica* JCM 10877	85.25	77.66	22.6
328.	*Nocardiopsis coralli* HNM0947	*Nocardiopsis exhalans* JCM11759	85.44	77.64	22.8
329.	*Nocardiopsis deserti* H13	*Nocardiopsis kunsanensis* DSM 44524	85.14	77.64	22.7
330.	*Nocardiopsis aegyptia* DSM 44442	*Nocardiopsis alkaliphila* YIM 80379	85.25	77.63	22.6
331.	*Nocardiopsis flavescens* CGMCC 4.5723	*Nocardiopsis listeri* NBRC 13360	85.19	77.63	22
332.	*Nocardiopsis lambiniae* DSM 44743	*Nocardiopsis listeri* NBRC 13360	85.27	77.59	22
333.	*Nocardiopsis litoralis* KCTC 19473	*Nocardiopsis quinghaiensis* YIM 28A4	85.15	77.59	22.2
334.	*Nocardiopsis coralli* HNM0947	*Nocardiopsis flavescens* CGMCC 4.5723	85.11	77.57	21.8
335.	*Nocardiopsis coralli* HNM0947	*Nocardiopsis metallicus* JCM 12409	85.49	77.56	23
336.	*Nocardiopsis kunsanensis* DSM 44524	*Nocardiopsis quinghaiensis* YIM 28A4	85.13	77.56	22.1
337.	*Nocardiopsis baichengensis* YIM 90130	*Nocardiopsis trehalosi* NBRC 14201	84.76	77.53	21.6
338.	*Nocardiopsis ganjiahuensis* DSM 45031	*Nocardiopsis litoralis* KCTC 19473	85.00	77.52	22.1
339.	*Nocardiopsis coralli* HNM0947	*Nocardiopsis ganjiahuensis* DSM 45031	85.27	77.49	22.6
340.	*Nocardiopsis arvandica* DSM 45278	*Nocardiopsis kunsanensis* DSM 44524	85.09	77.48	22.3
341.	*Nocardiopsis codii* CT-R113	*Nocardiopsis coralli* HNM0947	85.19	77.48	22.6
342.	*Nocardiopsis algeriensis* CECT 8712	*Nocardiopsis listeri* NBRC 13360	85.32	77.44	22.2
343.	*Nocardiopsis aegyptia* DSM 44442	*Nocardiopsis litoralis* KCTC 19473	85.09	77.40	22.3
344.	*Nocardiopsis exhalans* JCM11759	*Nocardiopsis kunsanensis* DSM 44524	85.13	77.39	22.5
345.	*Nocardiopsis halotolerans* DSM 44410	*Nocardiopsis kunsanensis* DSM 44524	85.30	77.39	20.4
346.	*Nocardiopsis ganjiahuensis* DSM 45031	*Nocardiopsis kunsanensis* DSM 44524	84.99	77.36	22.1
347.	*Nocardiopsis alkaliphila* YIM 80379	*Nocardiopsis changdeensis* Mg02	85.32	77.32	22

348.	*Nocardiopsis codii* CT-R113	*Nocardiopsis litoralis* KCTC 19473	84.96	77.28	22.1
349.	*Nocardiopsis aegyptia* DSM 44442	*Nocardiopsis coralli* HNM0947	85.25	77.25	22.5
350.	*Nocardiopsis algeriensis* CECT 8712	*Nocardiopsis alkaliphila* YIM 80379	85.22	77.25	22.2
351.	*Nocardiopsis alkaliphila* YIM 80379	*Nocardiopsis lambiniae* DSM 44743	85.19	77.24	22
352.	*Nocardiopsis coralli* HNM0947	*Nocardiopsis valliformis* DSM 45023	85.32	77.24	22.5
353.	*Nocardiopsis aegyptia* DSM 44442	*Nocardiopsis kunsanensis* DSM 44524	85.08	77.22	22.3
354.	*Nocardiopsis alkaliphila* YIM 80379	*Nocardiopsis flavescens* CGMCC 4.5723	85.25	77.21	21.9
355.	*Nocardiopsis changdeensis* Mg02	*Nocardiopsis coralli* HNM0947	85.24	77.15	22.2
356.	*Nocardiopsis codii* CT-R113	*Nocardiopsis kunsanensis* DSM 44524	84.94	77.12	22.1
357.	*Nocardiopsis litoralis* KCTC 19473	*Nocardiopsis terrae* KCTC 19431	85.13	77.12	21.9
358.	*Nocardiopsis kunsanensis* DSM 44524	*Nocardiopsis terrae* KCTC 19431	85.09	77.09	21.9
359.	*Nocardiopsis coralli* HNM0947	*Nocardiopsis lambiniae* DSM 44743	85.01	77.06	21.8
360.	*Nocardiopsis litoralis* KCTC 19473	*Nocardiopsis tropica* JCM 10877	84.98	77.06	21.9
361.	*Nocardiopsis rhodophaea* JCM 15313	*Nocardiopsis sediminis* TBRC 1826	85.15	77.04	22.6
362.	*Nocardiopsis coralli* HNM0947	*Nocardiopsis listeri* NBRC 13360	85.10	77.03	21.9
363.	*Nocardiopsis mangrovi* CGMCC 4.7119	*Nocardiopsis rhodophaea* JCM 15313	85.14	77.03	22.5
364.	*Nocardiopsis algeriensis* CECT 8712	*Nocardiopsis coralli* HNM0947	85.16	77.01	22.2
365.	*Nocardiopsis algeriensis* CECT 8712	*Nocardiopsis litoralis* KCTC 19473	85.05	77.00	21.5
366.	*Nocardiopsis changdeensis* Mg02	*Nocardiopsis litoralis* KCTC 19473	85.01	76.98	21.8
367.	*Nocardiopsis litoralis* KCTC 19473	*Nocardiopsis prasina* DSM 43845	85.05	76.98	21.8
368.	*Nocardiopsis kunsanensis* DSM 44524	*Nocardiopsis tropica* JCM 10877	84.95	76.95	22
369.	*Nocardiopsis listeri* NBRC 13360	*Nocardiopsis litoralis* KCTC 19473	85.01	76.94	21.5
370.	*Nocardiopsis kunsanensis* DSM 44524	*Nocardiopsis prasina* DSM 43845	85.05	76.91	21.8
371.	*Nocardiopsis alba* DSM 43377	*Nocardiopsis litoralis* KCTC 19473	85.08	76.84	21.4
372.	*Nocardiopsis changdeensis* Mg02	*Nocardiopsis kunsanensis* DSM 44524	85.03	76.84	21.8

373.	*Nocardiopsis flavescens* CGMCC 4.5723	*Nocardiopsis litoralis* KCTC 19473	84.92	76.84	21.5
374.	*Nocardiopsis litoralis* KCTC 19473	*Nocardiopsis metallicus* JCM 12409	85.15	76.83	22.5
375.	*Nocardiopsis litoralis* KCTC 19473	*Nocardiopsis valliformis* DSM 45023	85.18	76.74	22.5
376.	*Nocardiopsis algeriensis* CECT 8712	*Nocardiopsis kunsanensis* DSM 44524	85.07	76.73	21.5
377.	*Nocardiopsis lambiniae* DSM 44743	*Nocardiopsis litoralis* KCTC 19473	84.87	76.72	21.6
378.	*Nocardiopsis kunsanensis* DSM 44524	*Nocardiopsis listeri* NBRC 13360	85.00	76.71	21.5
379.	*Nocardiopsis flavescens* CGMCC 4.5723	*Nocardiopsis kunsanensis* DSM 44524	84.90	76.69	21.5
380.	*Nocardiopsis alba* DSM 43377	*Nocardiopsis kunsanensis* DSM 44524	85.03	76.68	21.4
381.	*Nocardiopsis mangrovi* CGMCC 4.7119	*Nocardiopsis potens* DSM 45234	84.89	76.64	22
382.	*Nocardiopsis kunsanensis* DSM 44524	*Nocardiopsis metallicus* JCM 12409	85.10	76.61	22.4
383.	*Nocardiopsis alba* DSM 43377	*Nocardiopsis coralli* HNM0947	85.25	76.56	22
384.	*Nocardiopsis kunsanensis* DSM 44524	*Nocardiopsis valliformis* DSM 45023	85.08	76.54	22.3
385.	*Nocardiopsis potens* DSM 45234	*Nocardiopsis rhodophaea* JCM 15313	84.94	76.48	22.2
386.	*Nocardiopsis potens* DSM 45234	*Nocardiopsis sediminis* TBRC 1826	84.82	76.48	21.8
387.	*Nocardiopsis kunsanensis* DSM 44524	*Nocardiopsis lambiniae* DSM 44743	84.91	76.47	21.7
388.	*Nocardiopsis deserti* H13	*Nocardiopsis trehalosi* NBRC 14201	84.78	76.46	22
389.	*Nocardiopsis dassonvillei* subsp. *crassaminis* D1	*Nocardiopsis trehalosi* NBRC 14201	84.78	76.42	22.1
390.	*Nocardiopsis dassonvillei* subsp. *dassonvillei* DSM 43111	*Nocardiopsis trehalosi* NBRC 14201	84.72	76.40	21.8
391.	*Nocardiopsis alborubida* ATCC 23612	*Nocardiopsis trehalosi* NBRC 14201	84.74	76.37	21.7
392.	*Nocardiopsis alkaliphila* YIM 80379	*Nocardiopsis litoralis* KCTC 19473	84.89	76.37	21.5
393.	*Nocardiopsis akebiae* HDS12	*Nocardiopsis trehalosi* NBRC 14201	84.68	76.34	21.8
394.	*Nocardiopsis alkaliphila* YIM 80379	*Nocardiopsis kunsanensis* DSM 44524	84.91	76.23	21.4
395.	*Nocardiopsis sinuspersici* UTMC 00102	*Nocardiopsis trehalosi* NBRC 14201	84.62	76.16	21.5
396.	*Nocardiopsis arvandica* DSM 45278	*Nocardiopsis trehalosi* NBRC 14201	84.61	76.12	21.6
397.	*Nocardiopsis changdeensis* Mg02	*Nocardiopsis trehalosi* NBRC 14201	84.69	76.08	21.2

398.	*Nocardiopsis aegyptia* DSM 44442	*Nocardiopsis trehalosi* NBRC 14201	84.64	76.07	21.5
399.	*Nocardiopsis alkaliphila* YIM 80379	*Nocardiopsis coralli* HNM0947	84.92	76.04	21.6
400.	*Nocardiopsis baichengensis* YIM 90130	*Nocardiopsis sediminis* TBRC 1826	84.76	76.04	21.6
401.	*Nocardiopsis flavescens* CGMCC 4.5723	*Nocardiopsis trehalosi* NBRC 14201	84.69	75.97	21.2
402.	*Nocardiopsis baichengensis* YIM 90130	*Nocardiopsis mangrovi* CGMCC 4.7119	84.72	75.95	21.8
403.	*Nocardiopsis quinghaiensis* YIM 28A4	*Nocardiopsis trehalosi* NBRC 14201	84.66	75.94	21.1
404.	*Nocardiopsis baichengensis* YIM 90130	*Nocardiopsis rhodophaea* JCM 15313	84.75	75.88	22
405.	*Nocardiopsis halotolerans* DSM 44410	*Nocardiopsis trehalosi* NBRC 14201	84.72	75.86	24.3
406.	*Nocardiopsis algeriensis* CECT 8712	*Nocardiopsis trehalosi* NBRC 14201	84.74	75.83	21.4
407.	*Nocardiopsis codii* CT-R113	*Nocardiopsis trehalosi* NBRC 14201	84.42	75.78	21.4
408.	*Nocardiopsis dassonvillei* subsp. *crassaminis* D1	*Nocardiopsis potens* DSM 45234	84.91	75.62	22.1
409.	*Nocardiopsis ganjiahuensis* DSM 45031	*Nocardiopsis trehalosi* NBRC 14201	84.38	75.62	21.3
410.	*Nocardiopsis prasina* DSM 43845	*Nocardiopsis trehalosi* NBRC 14201	84.46	75.62	21.5
411.	*Nocardiopsis alborubida* ATCC 23612	*Nocardiopsis potens* DSM 45234	84.97	75.61	22
412.	*Nocardiopsis deserti* H13	*Nocardiopsis potens* DSM 45234	84.79	75.60	22
413.	*Nocardiopsis dassonvillei* subsp. *dassonvillei* DSM 43111	*Nocardiopsis potens* DSM 45234	84.89	75.57	21.9
414.	*Nocardiopsis changdeensis* Mg02	*Nocardiopsis potens* DSM 45234	84.81	75.54	21.5
415.	*Nocardiopsis lambiniae* DSM 44743	*Nocardiopsis trehalosi* NBRC 14201	84.45	75.52	21.3
416.	*Nocardiopsis akebiae* HDS12	*Nocardiopsis potens* DSM 45234	84.92	75.49	22
417.	*Nocardiopsis akebiae* HDS12	*Nocardiopsis baichengensis* YIM 90130	84.55	75.48	21.5
418.	*Nocardiopsis arvandica* DSM 45278	*Nocardiopsis potens* DSM 45234	84.72	75.46	21.6
419.	*Nocardiopsis potens* DSM 45234	*Nocardiopsis sinuspersici* UTMC 00102	84.74	75.44	21.7
420.	*Nocardiopsis alborubida* ATCC 23612	*Nocardiopsis baichengensis* YIM 90130	84.54	75.38	21.3
421.	*Nocardiopsis arvandica* DSM 45278	*Nocardiopsis baichengensis* YIM 90130	84.47	75.38	21.3
422.	*Nocardiopsis exhalans* JCM11759	*Nocardiopsis trehalosi* NBRC 14201	84.43	75.37	21.6

423.	*Nocardiopsis dassonvillei* subsp. *crassaminis* D1	*Nocardiopsis sediminis* TBRC 1826	84.70	75.35	22.1
424.	*Nocardiopsis dassonvillei* subsp. *dassonvillei* DSM 43111	*Nocardiopsis sediminis* TBRC 1826	84.66	75.31	21.7
425.	*Nocardiopsis metallicus* JCM 12409	*Nocardiopsis trehalosi* NBRC 14201	84.35	75.30	21.3
426.	*Nocardiopsis mangrovi* CGMCC 4.7119	*Nocardiopsis sinuspersici* UTMC 00102	84.56	75.29	21.9
427.	*Nocardiopsis aegyptia* DSM 44442	*Nocardiopsis potens* DSM 45234	84.60	75.28	21.7
428.	*Nocardiopsis dassonvillei* subsp. *crassaminis* D1	*Nocardiopsis mangrovi* CGMCC 4.7119	84.73	75.25	22.3
429.	*Nocardiopsis terrae* KCTC 19431	*Nocardiopsis trehalosi* NBRC 14201	84.42	75.25	21.1
430.	*Nocardiopsis aegyptia* DSM 44442	*Nocardiopsis baichengensis* YIM 90130	84.45	75.22	21.3
431.	*Nocardiopsis flavescens* CGMCC 4.5723	*Nocardiopsis potens* DSM 45234	84.75	75.21	21.3
432.	*Nocardiopsis alborubida* ATCC 23612	*Nocardiopsis sediminis* TBRC 1826	84.63	75.17	21.8
433.	*Nocardiopsis deserti* H13	*Nocardiopsis sediminis* TBRC 1826	84.68	75.17	21.7
434.	*Nocardiopsis halotolerans* DSM 44410	*Nocardiopsis potens* DSM 45234	84.81	75.16	22
435.	*Nocardiopsis akebiae* HDS12	*Nocardiopsis sediminis* TBRC 1826	84.66	75.15	21.7
436.	*Nocardiopsis sediminis* TBRC 1826	*Nocardiopsis sinuspersici* UTMC 00102	84.59	75.15	21.6
437.	*Nocardiopsis dassonvillei* subsp. *dassonvillei* DSM 43111	*Nocardiopsis mangrovi* CGMCC 4.7119	84.76	75.11	22.1
438.	*Nocardiopsis ganjiahuensis* DSM 45031	*Nocardiopsis potens* DSM 45234	84.65	75.11	21.4
439.	*Nocardiopsis metallicus* JCM 12409	*Nocardiopsis potens* DSM 45234	84.71	75.05	21.6
440.	*Nocardiopsis baichengensis* YIM 90130	*Nocardiopsis sinuspersici* UTMC 00102	84.46	75.04	21.4
441.	*Nocardiopsis deserti* H13	*Nocardiopsis mangrovi* CGMCC 4.7119	84.67	75.04	22
442.	*Nocardiopsis potens* DSM 45234	*Nocardiopsis quinghaiensis* YIM 28A4	84.73	75.04	21.6
443.	*Nocardiopsis akebiae* HDS12	*Nocardiopsis mangrovi* CGMCC 4.7119	84.67	75.03	22.1
444.	*Nocardiopsis codii* CT-R113	*Nocardiopsis potens* DSM 45234	84.60	75.03	21.5
445.	*Nocardiopsis alborubida* ATCC 23612	*Nocardiopsis mangrovi* CGMCC 4.7119	84.65	75.02	21.9
446.	*Nocardiopsis arvandica* DSM 45278	*Nocardiopsis sediminis* TBRC 1826	84.59	75.01	21.7
447.	*Nocardiopsis algeriensis* CECT 8712	*Nocardiopsis baichengensis* YIM 90130	84.63	74.98	21.2

448.	*Nocardiopsis baichengensis* YIM 90130	*Nocardiopsis dassonvillei* subsp. *dassonvillei* DSM 43111	84.56	74.97	21.4
449.	*Nocardiopsis coralli* HNM0947	*Nocardiopsis trehalosi* NBRC 14201	84.32	74.97	20.7
450.	*Nocardiopsis alba* DSM 43377	*Nocardiopsis trehalosi* NBRC 14201	84.60	74.96	21
451.	*Nocardiopsis changdeensis* Mg02	*Nocardiopsis sediminis* TBRC 1826	84.71	74.96	21.5
452.	*Nocardiopsis mangrovi* CGMCC 4.7119	*Nocardiopsis quinghaiensis* YIM 28A4	84.65	74.92	21.5
453.	*Nocardiopsis flavescens* CGMCC 4.5723	*Nocardiopsis sediminis* TBRC 1826	84.81	74.91	21.4
454.	*Nocardiopsis exhalans* JCM11759	*Nocardiopsis potens* DSM 45234	84.56	74.89	21.6
455.	*Nocardiopsis aegyptia* DSM 44442	*Nocardiopsis sediminis* TBRC 1826	84.54	74.87	21.7
456.	*Nocardiopsis algeriensis* CECT 8712	*Nocardiopsis potens* DSM 45234	84.91	74.87	21.4
457.	*Nocardiopsis quinghaiensis* YIM 28A4	*Nocardiopsis sediminis* TBRC 1826	84.55	74.87	21.7
458.	*Nocardiopsis dassonvillei* subsp. *crassaminis* D1	*Nocardiopsis rhodophaea* JCM 15313	84.82	74.83	22.3
459.	*Nocardiopsis baichengensis* YIM 90130	*Nocardiopsis quinghaiensis* YIM 28A4	84.52	74.81	21.1
460.	*Nocardiopsis lambiniae* DSM 44743	*Nocardiopsis potens* DSM 45234	84.66	74.81	21.3
461.	*Nocardiopsis trehalosi* NBRC 14201	*Nocardiopsis tropica* JCM 10877	84.55	74.81	21.4
462.	*Nocardiopsis arvandica* DSM 45278	*Nocardiopsis mangrovi* CGMCC 4.7119	84.58	74.79	21.9
463.	*Nocardiopsis changdeensis* Mg02	*Nocardiopsis mangrovi* CGMCC 4.7119	84.72	74.79	21.6
464.	*Nocardiopsis aegyptia* DSM 44442	*Nocardiopsis mangrovi* CGMCC 4.7119	84.52	74.78	21.9
465.	*Nocardiopsis aegyptia* DSM 44442	*Nocardiopsis rhodophaea* JCM 15313	84.60	74.78	22.2
466.	*Nocardiopsis codii* CT-R113	*Nocardiopsis sediminis* TBRC 1826	84.63	74.77	21.8
467.	*Nocardiopsis codii* CT-R113	*Nocardiopsis mangrovi* CGMCC 4.7119	84.58	74.76	21.7
468.	*Nocardiopsis listeri* NBRC 13360	*Nocardiopsis trehalosi* NBRC 14201	84.36	74.74	20.6
469.	*Nocardiopsis rhodophaea* JCM 15313	*Nocardiopsis sinuspersici* UTMC 00102	84.61	74.74	22.2
470.	*Nocardiopsis baichengensis* YIM 90130	*Nocardiopsis deserti* H13	84.58	74.73	21.5
471.	*Nocardiopsis baichengensis* YIM 90130	*Nocardiopsis changdeensis* Mg02	84.60	74.71	21.2
472.	*Nocardiopsis baichengensis* YIM 90130	*Nocardiopsis dassonvillei* subsp. *crassaminis* D1	84.60	74.71	21.7

473.	*Nocardiopsis baichengensis* YIM 90130	*Nocardiopsis flavescens* CGMCC 4.5723	84.43	74.69	21
474.	*Nocardiopsis quinghaiensis* YIM 28A4	*Nocardiopsis rhodophaea* JCM 15313	84.69	74.67	21.9
475.	*Nocardiopsis dassonvillei* subsp. *dassonvillei* DSM 43111	*Nocardiopsis rhodophaea* JCM 15313	84.76	74.66	22.2
476.	*Nocardiopsis halotolerans* DSM 44410	*Nocardiopsis mangrovi* CGMCC 4.7119	84.69	74.64	25.2
477.	*Nocardiopsis akebiae* HDS12	*Nocardiopsis rhodophaea* JCM 15313	84.76	74.63	22
478.	*Nocardiopsis potens* DSM 45234	*Nocardiopsis prasina* DSM 43845	84.61	74.63	21.5
479.	*Nocardiopsis halotolerans* DSM 44410	*Nocardiopsis sediminis* TBRC 1826	84.68	74.62	22.8
480.	*Nocardiopsis arvandica* DSM 45278	*Nocardiopsis rhodophaea* JCM 15313	84.67	74.61	22.1
481.	*Nocardiopsis deserti* H13	*Nocardiopsis rhodophaea* JCM 15313	84.72	74.59	22.1
482.	*Nocardiopsis flavescens* CGMCC 4.5723	*Nocardiopsis mangrovi* CGMCC 4.7119	84.68	74.59	21.3
483.	*Nocardiopsis alborubida* ATCC 23612	*Nocardiopsis rhodophaea* JCM 15313	84.76	74.58	22.1
484.	*Nocardiopsis algeriensis* CECT 8712	*Nocardiopsis sediminis* TBRC 1826	84.74	74.58	21.4
485.	*Nocardiopsis algeriensis* CECT 8712	*Nocardiopsis mangrovi* CGMCC 4.7119	84.76	74.57	21.4
486.	*Nocardiopsis lambiniae* DSM 44743	*Nocardiopsis sediminis* TBRC 1826	84.57	74.56	21.3
487.	*Nocardiopsis mangrovi* CGMCC 4.7119	*Nocardiopsis tropica* JCM 10877	84.71	74.52	21.7
488.	*Nocardiopsis potens* DSM 45234	*Nocardiopsis terrae* KCTC 19431	84.64	74.52	21.5
489.	*Nocardiopsis litoralis* KCTC 19473	*Nocardiopsis trehalosi* NBRC 14201	84.20	74.51	20.7
490.	*Nocardiopsis alba* DSM 43377	*Nocardiopsis potens* DSM 45234	84.81	74.50	21.1
491.	*Nocardiopsis prasina* DSM 43845	*Nocardiopsis sediminis* TBRC 1826	84.60	74.50	21.5
492.	*Nocardiopsis algeriensis* CECT 8712	*Nocardiopsis rhodophaea* JCM 15313	84.74	74.49	22
493.	*Nocardiopsis changdeensis* Mg02	*Nocardiopsis rhodophaea* JCM 15313	84.88	74.49	22
494.	*Nocardiopsis potens* DSM 45234	*Nocardiopsis tropica* JCM 10877	84.69	74.49	21.5
495.	*Nocardiopsis halotolerans* DSM 44410	*Nocardiopsis rhodophaea* JCM 15313	84.71	74.48	32.2
496.	*Nocardiopsis sediminis* TBRC 1826	*Nocardiopsis tropica* JCM 10877	84.62	74.47	21.6
497.	*Nocardiopsis alba* DSM 43377	*Nocardiopsis baichengensis* YIM 90130	84.51	74.45	20.8

498.	*Nocardiopsis ganjiahuensis* DSM 45031	*Nocardiopsis sediminis* TBRC 1826	84.44	74.45	21.6
499.	*Nocardiopsis kunsanensis* DSM 44524	*Nocardiopsis trehalosi* NBRC 14201	84.19	74.42	20.6
500.	*Nocardiopsis metallicus* JCM 12409	*Nocardiopsis sediminis* TBRC 1826	84.50	74.42	21.5
501.	*Nocardiopsis coralli* HNM0947	*Nocardiopsis potens* DSM 45234	84.45	74.40	20.9
502.	*Nocardiopsis exhalans* JCM11759	*Nocardiopsis sediminis* TBRC 1826	84.47	74.40	21.6
503.	*Nocardiopsis lambiniae* DSM 44743	*Nocardiopsis mangrovi* CGMCC 4.7119	84.58	74.39	21.3
504.	*Nocardiopsis ganjiahuensis* DSM 45031	*Nocardiopsis mangrovi* CGMCC 4.7119	84.58	74.38	21.6
505.	*Nocardiopsis mangrovi* CGMCC 4.7119	*Nocardiopsis prasina* DSM 43845	84.50	74.38	21.6
506.	*Nocardiopsis alkaliphila* YIM 80379	*Nocardiopsis trehalosi* NBRC 14201	84.24	74.35	20.9
507.	*Nocardiopsis baichengensis* YIM 90130	*Nocardiopsis halotolerans* DSM 44410	84.56	74.35	21.4
508.	*Nocardiopsis codii* CT-R113	*Nocardiopsis rhodophaea* JCM 15313	84.62	74.35	21.9
509.	*Nocardiopsis prasina* DSM 43845	*Nocardiopsis rhodophaea* JCM 15313	84.65	74.34	21.8
510.	*Nocardiopsis exhalans* JCM11759	*Nocardiopsis mangrovi* CGMCC 4.7119	84.68	74.32	21.9
511.	*Nocardiopsis ganjiahuensis* DSM 45031	*Nocardiopsis rhodophaea* JCM 15313	84.53	74.32	21.8
512.	*Nocardiopsis metallicus* JCM 12409	*Nocardiopsis rhodophaea* JCM 15313	84.48	74.30	21.8
513.	*Nocardiopsis baichengensis* YIM 90130	*Nocardiopsis lambiniae* DSM 44743	84.37	74.26	20.9
514.	*Nocardiopsis flavescens* CGMCC 4.5723	*Nocardiopsis rhodophaea* JCM 15313	84.61	74.26	21.6
515.	*Nocardiopsis exhalans* JCM11759	*Nocardiopsis rhodophaea* JCM 15313	84.53	74.24	22.1
516.	*Nocardiopsis lambiniae* DSM 44743	*Nocardiopsis rhodophaea* JCM 15313	84.68	74.24	21.6
517.	*Nocardiopsis mangrovi* CGMCC 4.7119	*Nocardiopsis terrae* KCTC 19431	84.51	74.23	21.5
518.	*Nocardiopsis listeri* NBRC 13360	*Nocardiopsis potens* DSM 45234	84.48	74.21	21.1
519.	*Nocardiopsis potens* DSM 45234	*Nocardiopsis valliformis* DSM 45023	84.45	74.19	21.6
520.	*Nocardiopsis sediminis* TBRC 1826	*Nocardiopsis terrae* KCTC 19431	84.50	74.17	21.2
521.	*Nocardiopsis baichengensis* YIM 90130	*Nocardiopsis terrae* KCTC 19431	84.40	74.12	21.1
522.	*Nocardiopsis rhodophaea* JCM 15313	*Nocardiopsis terrae* KCTC 19431	84.55	74.10	21.7

523.	*Nocardiopsis baichengensis* YIM 90130	*Nocardiopsis prasina* DSM 43845	84.46	74.03	21
524.	*Nocardiopsis baichengensis* YIM 90130	*Nocardiopsis tropica* JCM 10877	84.33	74.02	21
525.	*Nocardiopsis baichengensis* YIM 90130	*Nocardiopsis codii* CT-R113	84.32	73.97	21.1
526.	*Nocardiopsis trehalosi* NBRC 14201	*Nocardiopsis valliformis* DSM 45023	84.35	73.94	21.3
527.	*Nocardiopsis alba* DSM 43377	*Nocardiopsis rhodophaea* JCM 15313	84.75	73.90	21.6
528.	*Nocardiopsis coralli* HNM0947	*Nocardiopsis rhodophaea* JCM 15313	84.42	73.90	21.4
529.	*Nocardiopsis rhodophaea* JCM 15313	*Nocardiopsis tropica* JCM 10877	84.74	73.90	21.9
530.	*Nocardiopsis mangrovi* CGMCC 4.7119	*Nocardiopsis metallicus* JCM 12409	84.43	73.87	21.7
531.	*Nocardiopsis alkaliphila* YIM 80379	*Nocardiopsis baichengensis* YIM 90130	84.32	73.86	20.7
532.	*Nocardiopsis litoralis* KCTC 19473	*Nocardiopsis potens* DSM 45234	84.32	73.84	21
533.	*Nocardiopsis alba* DSM 43377	*Nocardiopsis sediminis* TBRC 1826	84.52	73.83	20.8
534.	*Nocardiopsis alba* DSM 43377	*Nocardiopsis mangrovi* CGMCC 4.7119	84.61	73.82	21
535.	*Nocardiopsis listeri* NBRC 13360	*Nocardiopsis rhodophaea* JCM 15313	84.40	73.81	21.4
536.	*Nocardiopsis mangrovi* CGMCC 4.7119	*Nocardiopsis valliformis* DSM 45023	84.51	73.81	21.7
537.	*Nocardiopsis sediminis* TBRC 1826	*Nocardiopsis valliformis* DSM 45023	84.36	73.81	21.3
538.	*Nocardiopsis kunsanensis* DSM 44524	*Nocardiopsis potens* DSM 45234	84.33	73.79	21
539.	*Nocardiopsis coralli* HNM0947	*Nocardiopsis sediminis* TBRC 1826	84.30	73.78	20.8
540.	*Nocardiopsis baichengensis* YIM 90130	*Nocardiopsis metallicus* JCM 12409	84.20	73.75	20.9
541.	*Nocardiopsis listeri* NBRC 13360	*Nocardiopsis sediminis* TBRC 1826	84.24	73.75	20.9
542.	*Nocardiopsis baichengensis* YIM 90130	*Nocardiopsis ganjiahuensis* DSM 45031	84.34	73.72	20.9
543.	*Nocardiopsis baichengensis* YIM 90130	*Nocardiopsis litoralis* KCTC 19473	84.20	73.72	20.9
544.	*Nocardiopsis alkaliphila* YIM 80379	*Nocardiopsis potens* DSM 45234	84.42	73.70	21
545.	*Nocardiopsis coralli* HNM0947	*Nocardiopsis mangrovi* CGMCC 4.7119	84.42	73.70	21
546.	*Nocardiopsis baichengensis* YIM 90130	*Nocardiopsis exhalans* JCM11759	84.3	73.67	21
547.	*Nocardiopsis listeri* NBRC 13360	*Nocardiopsis mangrovi* CGMCC 4.7119	84.23	73.67	21.1

548.	*Nocardiopsis rhodophaea* JCM 15313	*Nocardiopsis valliformis* DSM 45023	84.53	73.67	22.1
549.	*Nocardiopsis baichengensis* YIM 90130	*Nocardiopsis coralli* HNM0947	84.25	73.60	20.6
550.	*Nocardiopsis baichengensis* YIM 90130	*Nocardiopsis valliformis* DSM 45023	84.24	73.57	20.7
551.	*Nocardiopsis litoralis* KCTC 19473	*Nocardiopsis mangrovi* CGMCC 4.7119	84.20	73.52	21.1
552.	*Nocardiopsis baichengensis* YIM 90130	*Nocardiopsis listeri* NBRC 13360	84.28	73.48	20.6
553.	*Nocardiopsis litoralis* KCTC 19473	*Nocardiopsis sediminis* TBRC 1826	84.16	73.47	20.6
554.	*Nocardiopsis alkaliphila* YIM 80379	*Nocardiopsis sediminis* TBRC 1826	84.13	73.46	20.9
555.	*Nocardiopsis baichengensis* YIM 90130	*Nocardiopsis kunsanensis* DSM 44524	84.24	73.45	20.8
556.	*Nocardiopsis kunsanensis* DSM 44524	*Nocardiopsis mangrovi* CGMCC 4.7119	84.20	73.45	21.1
557.	*Nocardiopsis kunsanensis* DSM 44524	*Nocardiopsis rhodophaea* JCM 15313	84.26	73.44	21.2
558.	*Nocardiopsis kunsanensis* DSM 44524	*Nocardiopsis sediminis* TBRC 1826	84.18	73.41	20.6
559.	*Nocardiopsis alkaliphila* YIM 80379	*Nocardiopsis mangrovi* CGMCC 4.7119	84.19	73.39	21.1
560.	*Nocardiopsis litoralis* KCTC 19473	*Nocardiopsis rhodophaea* JCM 15313	84.26	73.39	21.3
561.	*Nocardiopsis alkaliphila* YIM 80379	*Nocardiopsis rhodophaea* JCM 15313	84.32	73.35	21.2

Note: Completeness >90%, Contamination<10%.

Table S6. ANI and dDDH values of 780 pairs of *Nocardiopsis* species.

No.	Strain 1	Strain 2	ANIm	ANIb	dDDH
1.	*Nocardiopsis kunsanensis* DSM 44524	*Nocardiopsis litoralis* KCTC 19473	99.48	99.25	95.7
2.	*Nocardiopsis arvandica* DSM 45278	*Nocardiopsis sinuspersici* UTMC 00102	99.05	98.41	90.8
3.	*Nocardiopsis baichengensis* YIM 90130	*Nocardiopsis halophila* DSM 44494	98.25	97.68	82.9
4.	*Nocardiopsis dassonvillei* subsp. *crassaminis* D1	*Nocardiopsis dassonvillei* subsp. *dassonvillei* DSM 43111	97.83	97.43	79.50
5.	*Nocardiopsis exhalans* JCM11759	*Nocardiopsis valliformis* DSM 45023	96.33	95.24	66.6
6.	*Nocardiopsis alborubida* ATCC 23612	*Nocardiopsis exhalans* JCM11759	95.58	94.61	62.20
7.	*Nocardiopsis akebiae* HDS12	*Nocardiopsis dassonvillei* subsp. *dassonvillei* DSM 43111	95.35	94.54	60.40
8.	*Nocardiopsis exhalans* JCM11759	*Nocardiopsis metallicus* JCM 12409	95.57	94.46	61.2
9.	*Nocardiopsis akebiae* HDS12	*Nocardiopsis dassonvillei* subsp. *crassaminis* D1	95.39	94.27	60.50
10.	*Nocardiopsis metallicus* JCM 12409	*Nocardiopsis valliformis* DSM 45023	94.98	93.67	57.7
11.	*Nocardiopsis alborubida* ATCC 23612	*Nocardiopsis deserti* H13	94.27	93.54	53.60
12.	*Nocardiopsis alborubida* ATCC 23612	*Nocardiopsis dassonvillei* subsp. *dassonvillei* DSM 43111	94.32	93.14	53.70
13.	*Nocardiopsis dassonvillei* subsp. *crassaminis* D1	*Nocardiopsis deserti* H13	94.21	92.67	52.90
14.	*Nocardiopsis dassonvillei* subsp. *dassonvillei* DSM 43111	*Nocardiopsis deserti* H13	94.14	92.63	52.60
15.	*Nocardiopsis akebiae* HDS12	*Nocardiopsis alborubida* ATCC 23612	93.72	92.32	50.50
16.	*Nocardiopsis akebiae* HDS12	*Nocardiopsis deserti* H13	93.68	92.28	50.40
17.	*Nocardiopsis arvandica* DSM 45278	*Nocardiopsis quinghaiensis* YIM 28A4	92.89	91.78	47.7
18.	*Nocardiopsis quinghaiensis* YIM 28A4	*Nocardiopsis sinuspersici* UTMC 00102	92.88	91.78	47.5
19.	*Nocardiopsis coralli* HNM0947	*Nocardiopsis salina* YIM 90010	92.26	91.32	45.30
20.	*Nocardiopsis gilva* YIM 90087	*Nocardiopsis rhodophaea* JCM 15313	91.36	89.98	41.3
21.	*Nocardiopsis kunsanensis* DSM 44524	*Nocardiopsis xinjiangensis* YIM 90004	90.95	89.96	40.5
22.	*Nocardiopsis litoralis* KCTC 19473	*Nocardiopsis xinjiangensis* YIM 90004	90.89	89.87	40.3
23.	*Nocardiopsis codii* CT-R113	*Nocardiopsis tropica* JCM 10877	90.90	89.40	38.5

24.	*Nocardiopsis mangrovi* CGMCC 4.7119	*Nocardiopsis sediminis* TBRC 1826	90.88	89.34	38.9
25.	*Nocardiopsis changdeensis* Mg02	*Nocardiopsis flavescens* CGMCC 4.5723	90.11	88.49	36.7
26.	*Nocardiopsis deserti* H13	*Nocardiopsis halotolerans* DSM 44410	89.57	87.15	34.2
27.	*Nocardiopsis alborubida* ATCC 23612	*Nocardiopsis halotolerans* DSM 44410	89.53	87.02	34.10
28.	*Nocardiopsis dassonvillei* subsp. *crassaminis* D1	*Nocardiopsis halotolerans* DSM 44410	89.61	86.95	34.50
29.	*Nocardiopsis ganjiahuensis* DSM 45031	*Nocardiopsis metallicus* JCM 12409	89.57	86.92	34
30.	*Nocardiopsis dassonvillei* subsp. *dassonvillei* DSM 43111	*Nocardiopsis halotolerans* DSM 44410	89.49	86.81	34.10
31.	*Nocardiopsis ganjiahuensis* DSM 45031	*Nocardiopsis valliformis* DSM 45023	89.53	86.69	33.7
32.	*Nocardiopsis exhalans* JCM11759	*Nocardiopsis ganjiahuensis* DSM 45031	89.65	86.67	34.3
33.	*Nocardiopsis halotolerans* DSM 44410	*Nocardiopsis sinuspersici* UTMC 00102	89.02	86.66	21.7
34.	*Nocardiopsis deserti* H13	*Nocardiopsis sinuspersici* UTMC 00102	89.02	86.56	32.2
35.	*Nocardiopsis alborubida* ATCC 23612	*Nocardiopsis arvandica* DSM 45278	88.94	86.39	32.10
36.	*Nocardiopsis ganjiahuensis* DSM 45031	*Nocardiopsis terrae* KCTC 19431	88.85	86.39	32.2
37.	*Nocardiopsis halotolerans* DSM 44410	*Nocardiopsis quinghaiensis* YIM 28A4	88.90	86.39	24.4
38.	*Nocardiopsis akebiae* HDS12	*Nocardiopsis halotolerans* DSM 44410	89.28	86.38	33.50
39.	*Nocardiopsis dassonvillei* subsp. *crassaminis* D1	*Nocardiopsis sinuspersici* UTMC 00102	89.02	86.38	32.5
40.	*Nocardiopsis alborubida* ATCC 23612	*Nocardiopsis sinuspersici* UTMC 00102	88.93	86.37	32.1
41.	*Nocardiopsis dassonvillei* subsp. *dassonvillei* DSM 43111	*Nocardiopsis sinuspersici* UTMC 00102	88.87	86.20	32
42.	*Nocardiopsis deserti* H13	*Nocardiopsis quinghaiensis* YIM 28A4	88.90	86.16	32.3
43.	*Nocardiopsis arvandica* DSM 45278	*Nocardiopsis halotolerans* DSM 44410	88.99	86.07	32.5
44.	*Nocardiopsis alborubida* ATCC 23612	*Nocardiopsis quinghaiensis* YIM 28A4	88.92	85.99	32
45.	*Nocardiopsis dassonvillei* subsp. *crassaminis* D1	*Nocardiopsis quinghaiensis* YIM 28A4	88.98	85.94	32.5
46.	*Nocardiopsis akebiae* HDS12	*Nocardiopsis sinuspersici* UTMC 00102	88.72	85.91	31.6
47.	*Nocardiopsis arvandica* DSM 45278	*Nocardiopsis dassonvillei* subsp. *dassonvillei* DSM 43111	88.87	85.88	32
48.	*Nocardiopsis dassonvillei* subsp. dassonvillei DSM 43111	*Nocardiopsis quinghaiensis* YIM 28A4	88.81	85.84	31.9

49.	*Nocardiopsis arvandica* DSM 45278	*Nocardiopsis dassonvillei* subsp. *crassaminis* D1	89.01	85.82	32.5
50.	*Nocardiopsis akebiae* HDS12	*Nocardiopsis arvandica* DSM 45278	88.69	85.81	31.50
51.	*Nocardiopsis arvandica* DSM 45278	*Nocardiopsis deserti* H13	89.02	85.62	32.2
52.	*Nocardiopsis akebiae* HDS12	*Nocardiopsis quinghaiensis* YIM 28A4	88.61	85.61	31.3
53.	*Nocardiopsis metallicus* JCM 12409	*Nocardiopsis terrae* KCTC 19431	88.35	85.50	30.5
54.	*Nocardiopsis coralli* HNM0947	*Nocardiopsis xinjiangensis* YIM 90004	87.85	85.49	30.80
55.	*Nocardiopsis exhalans* JCM11759	*Nocardiopsis terrae* KCTC 19431	88.37	85.38	30.5
56.	*Nocardiopsis ganjiahuensis* DSM 45031	*Nocardiopsis prasina* DSM 43845	88.40	85.37	30.9
57.	*Nocardiopsis changdeensis* Mg02	*Nocardiopsis lambiniae* DSM 44743	88.04	85.32	30.4
58.	*Nocardiopsis coralli* HNM0947	*Nocardiopsis litoralis* KCTC 19473	87.55	84.98	30
59.	*Nocardiopsis exhalans* JCM11759	*Nocardiopsis prasina* DSM 43845	88.21	84.93	30.2
60.	*Nocardiopsis metallicus* JCM 12409	*Nocardiopsis prasina* DSM 43845	88.13	84.92	30.1
61.	*Nocardiopsis coralli* HNM0947	*Nocardiopsis kunsanensis* DSM 44524	87.56	84.90	29.9
62.	*Nocardiopsis salina* YIM 90010	*Nocardiopsis xinjiangensis* YIM 90004	87.42	84.89	29.6
63.	*Nocardiopsis terrae* KCTC 19431	*Nocardiopsis valliformi*s DSM 45023	88.28	84.57	30.3
64.	*Nocardiopsis gilva* YIM 90087	*Nocardiopsis mwathae* DSM 46659	87.61	84.52	28.8
65.	*Nocardiopsis flavescens* CGMCC 4.5723	*Nocardiopsis lambiniae* DSM 44743	87.58	84.50	29.1
66.	*Nocardiopsis prasina* DSM 43845	*Nocardiopsis valliformi*s DSM 45023	88.10	84.19	29.8
67.	*Nocardiopsis prasina* DSM 43845	*Nocardiopsis terrae* KCTC 19431	87.69	84.16	29.0
68.	*Nocardiopsis kunsanensis* DSM 44524	*Nocardiopsis salina* YIM 90010	87.16	83.85	28.6
69.	*Nocardiopsis mwathae* DSM 46659	*Nocardiopsis rhodophaea* JCM 15313	87.37	83.85	28.1
70.	*Nocardiopsis litoralis* KCTC 19473	*Nocardiopsis salina* YIM 90010	87.17	83.83	28.6
71.	*Nocardiopsis codii* CT-R113	*Nocardiopsis dassonvillei* subsp. *dassonville*i DSM 43111	87.01	83.27	27.5
72.	*Nocardiopsis dassonvillei* subsp. *crassaminis* D1	*Nocardiopsis tropica* JCM 10877	87.19	83.11	28
73.	*Nocardiopsis codii* CT-R113	*Nocardiopsis sinuspersici* UTMC 00102	86.93	83.08	27.5

74.	*Nocardiopsis dassonvillei* subsp. *dassonvillei* DSM 43111	*Nocardiopsis tropica* JCM 10877	87.07	83.08	27.3
75.	*Nocardiopsis deserti* H13	*Nocardiopsis tropica* JCM 10877	87.20	83.04	27.7
76.	*Nocardiopsis codii* CT-R113	*Nocardiopsis dassonvillei* subsp. *crassaminis* D1	87.16	83.03	27.9
77.	*Nocardiopsis alborubida* ATCC 23612	*Nocardiopsis tropica* JCM 10877	87.19	83.00	27.6
78.	*Nocardiopsis codii* CT-R113	*Nocardiopsis quinghaiensis* YIM 28A4	87.00	82.96	27.4
79.	*Nocardiopsis akebiae* HDS12	*Nocardiopsis tropica* JCM 10877	87.04	82.87	27.3
80.	*Nocardiopsis codii* CT-R113	*Nocardiopsis deserti* H13	87.10	82.70	27.6
81.	*Nocardiopsis alborubida* ATCC 23612	*Nocardiopsis codii* CT-R113	87.07	82.63	27.50
82.	*Nocardiopsis arvandica* DSM 45278	*Nocardiopsis codii* CT-R113	86.95	82.50	27.4
83.	*Nocardiopsis arvandica* DSM 45278	*Nocardiopsis tropica* JCM 10877	86.96	82.47	27.2
84.	*Nocardiopsis akebiae* HDS12	*Nocardiopsis codii* CT-R113	86.95	82.46	27.00
85.	*Nocardiopsis sinuspersici* UTMC 00102	*Nocardiopsis tropica* JCM 10877	86.96	82.44	27.1
86.	*Nocardiopsis alkaliphila* YIM 80379	*Nocardiopsis listeri* NBRC 13360	86.54	82.36	26.3
87.	*Nocardiopsis codii* CT-R113	*Nocardiopsis halotolerans* DSM 44410	86.87	82.34	27.2
88.	*Nocardiopsis quinghaiensis* YIM 28A4	*Nocardiopsis tropica* JCM 10877	86.94	82.34	27.0
89.	*Nocardiopsis halotolerans* DSM 44410	*Nocardiopsis tropica* JCM 10877	86.79	82.09	21.7
90.	*Nocardiopsis aegyptia* DSM 44442	*Nocardiopsis arvandica* DSM 45278	86.47	81.25	25.50
91.	*Nocardiopsis alborubida* ATCC 23612	*Nocardiopsis algeriensis* CECT 8712	86.33	81.23	25.30
92.	*Nocardiopsis aegyptia* DSM 44442	*Nocardiopsis sinuspersici* UTMC 00102	86.48	81.22	25.40
93.	*Nocardiopsis akebiae* HDS12	*Nocardiopsis algeriensis* CECT 8712	86.26	81.2	25.20
94.	*Nocardiopsis ganjiahuensis* DSM 45031	*Nocardiopsis listeri* NBRC 13360	86.24	81.17	25.1
95.	*Nocardiopsis sediminis* TBRC 1826	*Nocardiopsis trehalosi* NBRC 14201	86.00	81.14	24.4
96.	*Nocardiopsis mangrovi* CGMCC 4.7119	*Nocardiopsis trehalosi* NBRC 14201	85.89	81.10	24.5
97.	*Nocardiopsis aegyptia* DSM 44442	*Nocardiopsis quinghaiensis* YIM 28A4	86.26	81.08	25.00
98.	*Nocardiopsis exhalans* JCM11759	*Nocardiopsis listeri* NBRC 13360	86.29	81.05	25.1

99.	*Nocardiopsis alba* DSM 43377	*Nocardiopsis terrae* KCTC 19431	86.39	81.01	25
100.	*Nocardiopsis aegyptia* DSM 44442	*Nocardiopsis dassonvillei* subsp. *dassonvillei* DSM 43111	86.45	80.98	25.30
101.	*Nocardiopsis listeri* NBRC 13360	*Nocardiopsis terrae* KCTC 19431	86.26	80.97	24.7
102.	*Nocardiopsis alba* DSM 43377	*Nocardiopsis alkaliphila* YIM 80379	86.15	80.95	24.90
103.	*Nocardiopsis alba* DSM 43377	*Nocardiopsis listeri* NBRC 13360	86.23	80.89	24.90
104.	*Nocardiopsis algeriensis* CECT 8712	*Nocardiopsis sinuspersici* UTMC 00102	86.20	80.80	24.9
105.	*Nocardiopsis aegyptia* DSM 44442	*Nocardiopsis akebiae* HDS12	86.43	80.79	25.20
106.	*Nocardiopsis aegyptia* DSM 44442	*Nocardiopsis dassonvillei* subsp. *crassaminis* D1	86.51	80.77	25.80
107.	*Nocardiopsis algeriensis* CECT 8712	*Nocardiopsis dassonvillei* subsp. *dassonvillei* DSM 43111	86.39	80.77	25.4
108.	*Nocardiopsis changdeensis* Mg02	*Nocardiopsis dassonvillei* subsp. *dassonvillei* DSM 43111	86.29	80.74	24.7
109.	*Nocardiopsis algeriensis* CECT 8712	*Nocardiopsis arvandica* DSM 45278	86.21	80.72	24.90
110.	*Nocardiopsis algeriensis* CECT 8712	*Nocardiopsis dassonvillei* subsp. *crassaminis* D1	86.51	80.61	25.8
111.	*Nocardiopsis alba* DSM 43377	*Nocardiopsis exhalans* JCM11759	86.5	80.59	25.10
112.	*Nocardiopsis alba* DSM 43377	*Nocardiopsis prasina* DSM 43845	86.44	80.59	24.7
113.	*Nocardiopsis dassonvillei* subsp. *crassaminis* D1	*Nocardiopsis flavescens* CGMCC 4.5723	86.23	80.54	24.80
114.	*Nocardiopsis lucentensis* DSM 44048	*Nocardiopsis sediminis* TBRC 1826	86.20	80.54	25.2
115.	*Nocardiopsis algeriensis* CECT 8712	*Nocardiopsis quinghaiensis* YIM 28A4	86.10	80.52	24.7
116.	*Nocardiopsis ganjiahuensis* DSM 45031	*Nocardiopsis sinuspersici* UTMC 00102	86.20	80.50	24.5
117.	*Nocardiopsis alkaliphila* YIM 80379	*Nocardiopsis terrae* KCTC 19431	86.01	80.49	24.5
118.	*Nocardiopsis changdeensis* Mg02	*Nocardiopsis sinuspersici* UTMC 00102	86.19	80.48	24.2
119.	*Nocardiopsis listeri* NBRC 13360	*Nocardiopsis metallicus* JCM 12409	86.29	80.47	25
120.	*Nocardiopsis aegyptia* DSM 44442	*Nocardiopsis deserti* H13	86.46	80.45	25.40
121.	*Nocardiopsis dassonvillei* subsp. *dassonvillei* DSM 43111	*Nocardiopsis flavescens* CGMCC 4.5723	86.17	80.43	24.20
122.	*Nocardiopsis changdeensis* Mg02	*Nocardiopsis dassonvillei* subsp. *crassaminis* D1	86.39	80.41	25.2
123.	*Nocardiopsis aegyptia* DSM 44442	*Nocardiopsis alborubida* ATCC 23612	86.49	80.39	25.30

124.	*Nocardiopsis alba* DSM 43377	*Nocardiopsis ganjiahuensis* DSM 45031	86.47	80.39	25.30
125.	*Nocardiopsis alba* DSM 43377	*Nocardiopsis metallicus* JCM 12409	86.47	80.39	25.10
126.	*Nocardiopsis alborubida* ATCC 23612	*Nocardiopsis changdeensis* Mg02	86.23	80.37	24.70
127.	*Nocardiopsis alborubida* ATCC 23612	*Nocardiopsis flavescens* CGMCC 4.5723	86.17	80.36	24.40
128.	*Nocardiopsis deserti* H13	*Nocardiopsis flavescens* CGMCC 4.5723	86.19	80.33	24.5
129.	*Nocardiopsis sinuspersici* UTMC 00102	*Nocardiopsis terrae* KCTC 19431	86.23	80.29	24.3
130.	*Nocardiopsis aegyptia* DSM 44442	*Nocardiopsis halotolerans* DSM 44410	86.22	80.28	25.10
131.	*Nocardiopsis aegyptia* DSM 44442	*Nocardiopsis lucentensis* DSM 44048	86.32	80.28	25.00
132.	*Nocardiopsis algeriensis* CECT 8712	*Nocardiopsis deserti* H13	86.34	80.28	25.5
133.	*Nocardiopsis akebiae* HDS12	*Nocardiopsis flavescens* CGMCC 4.5723	86.16	80.27	24.20
134.	*Nocardiopsis exhalans* JCM11759	*Nocardiopsis sinuspersici* UTMC 00102	86.18	80.26	24.6
135.	*Nocardiopsis listeri* NBRC 13360	*Nocardiopsis valliformi*s DSM 45023	86.22	80.26	24.9
136.	*Nocardiopsis akebiae* HDS12	*Nocardiopsis changdeensis* Mg02	86.34	80.25	24.70
137.	*Nocardiopsis dassonvillei* subsp. *crassaminis* D1	*Nocardiopsis lucentensis* DSM 44048	86.47	80.25	25.60
138.	*Nocardiopsis dassonvillei* subsp. *crassaminis* D1	*Nocardiopsis terrae* KCTC 19431	86.33	80.24	25
139.	*Nocardiopsis exhalans* JCM11759	*Nocardiopsis quinghaiensis* YIM 28A4	86.16	80.24	24.4
140.	*Nocardiopsis listeri* NBRC 13360	*Nocardiopsis prasina* DSM 43845	86.00	80.24	24.2
141.	*Nocardiopsis quinghaiensis* YIM 28A4	*Nocardiopsis terrae* KCTC 19431	86.16	80.23	24.1
142.	*Nocardiopsis changdeensis* Mg02	*Nocardiopsis quinghaiensis* YIM 28A4	85.99	80.22	23.9
143.	*Nocardiopsis aegyptia* DSM 44442	*Nocardiopsis tropica* JCM 10877	86.14	80.21	24.80
144.	*Nocardiopsis dassonvillei* subsp. *crassaminis* D1	*Nocardiopsis lambiniae* DSM 44743	86.18	80.21	24.80
145.	*Nocardiopsis lucentensis* DSM 44048	*Nocardiopsis prasina* DSM 43845	86.22	80.21	24.8
146.	*Nocardiopsis algeriensis* CECT 8712	*Nocardiopsis changdeensis* Mg02	86.37	80.2	24.60
147.	*Nocardiopsis metallicus* JCM 12409	*Nocardiopsis sinuspersici* UTMC 00102	86.08	80.19	24.3
148.	*Nocardiopsis alkaliphila* YIM 80379	*Nocardiopsis exhalans* JCM11759	86.12	80.18	24.9

149.	*Nocardiopsis deserti* H13	*Nocardiopsis terrae* KCTC 19431	86.27	80.18	24.6
150.	*Nocardiopsis algeriensis* CECT 8712	*Nocardiopsis flavescens* CGMCC 4.5723	86.11	80.17	24.1
151.	*Nocardiopsis alkaliphila* YIM 80379	*Nocardiopsis metallicus* JCM 12409	86.07	80.17	24.9
152.	*Nocardiopsis arvandica* DSM 45278	*Nocardiopsis terrae* KCTC 19431	86.25	80.17	24.3
153.	*Nocardiopsis ganjiahuensis* DSM 45031	*Nocardiopsis quinghaiensis* YIM 28A4	86.14	80.17	24.2
154.	*Nocardiopsis changdeensis* Mg02	*Nocardiopsis deserti* H13	86.26	80.16	24.7
155.	*Nocardiopsis codii* CT-R113	*Nocardiopsis flavescens* CGMCC 4.5723	85.76	80.15	23.6
156.	*Nocardiopsis arvandica* DSM 45278	*Nocardiopsis lucentensis* DSM 44048	86.20	80.14	25.2
157.	*Nocardiopsis alkaliphila* YIM 80379	*Nocardiopsis ganjiahuensis* DSM 45031	86.10	80.13	24.9
158.	*Nocardiopsis lambiniae* DSM 44743	*Nocardiopsis sinuspersici* UTMC 00102	85.82	80.13	24.1
159.	*Nocardiopsis alborubida* ATCC 23612	*Nocardiopsis lambiniae* DSM 44743	86.06	80.11	24.50
160.	*Nocardiopsis alkaliphila* YIM 80379	*Nocardiopsis valliformi*s DSM 45023	86.13	80.11	24.8
161.	*Nocardiopsis alborubida* ATCC 23612	*Nocardiopsis terrae* KCTC 19431	86.21	80.09	24.5
162.	*Nocardiopsis flavescens* CGMCC 4.5723	*Nocardiopsis sinuspersici* UTMC 00102	85.96	80.09	23.8
163.	*Nocardiopsis alba* DSM 43377	*Nocardiopsis valliformi*s DSM 45023	86.40	80.08	24.9
164.	*Nocardiopsis deserti* H13	*Nocardiopsis lucentensis* DSM 44048	86.43	80.06	25.2
165.	*Nocardiopsis metallicus* JCM 12409	*Nocardiopsis quinghaiensis* YIM 28A4	86.12	80.06	24.3
166.	*Nocardiopsis alborubida* ATCC 23612	*Nocardiopsis lucentensis* DSM 44048	86.38	80.05	25.30
167.	*Nocardiopsis arvandica* DSM 45278	*Nocardiopsis flavescens* CGMCC 4.5723	85.95	80.05	23.9
168.	*Nocardiopsis dassonvillei* subsp. *dassonvillei* DSM 43111	*Nocardiopsis lambiniae* DSM 44743	86.02	80.04	24.40
169.	*Nocardiopsis arvandica* DSM 45278	*Nocardiopsis changdeensis* Mg02	86.12	80.03	24.2
170.	*Nocardiopsis algeriensis* CECT 8712	*Nocardiopsis halotolerans* DSM 44410	86.15	80.02	25
171.	*Nocardiopsis dassonvillei* subsp. *dassonville*i DSM 43111	*Nocardiopsis terrae* KCTC 19431	86.20	80.01	24.5
172.	*Nocardiopsis deserti* H13	*Nocardiopsis lambiniae* DSM 44743	86.02	79.99	24.3
173.	*Nocardiopsis dassonvillei* subsp. *dassonvillei* DSM 43111	*Nocardiopsis lucentensis* DSM 44048	86.41	79.96	25.20

174.	*Nocardiopsis algeriensis* CECT 8712	*Nocardiopsis lambiniae* DSM 44743	86.10	79.95	24.2
175.	*Nocardiopsis halotolerans* DSM 44410	*Nocardiopsis lucentensis* DSM 44048	86.32	79.95	22.7
176.	*Nocardiopsis aegyptia* DSM 44442	*Nocardiopsis codii* CT-R113	86.12	79.93	24.80
177.	*Nocardiopsis akebiae* HDS12	*Nocardiopsis lucentensis* DSM 44048	86.4	79.93	25.20
178.	*Nocardiopsis akebiae* HDS12	*Nocardiopsis terrae* KCTC 19431	86.17	79.92	24.4
179.	*Nocardiopsis codii* CT-R113	*Nocardiopsis terrae* KCTC 19431	86.03	79.91	24.2
180.	*Nocardiopsis flavescens* CGMCC 4.5723	*Nocardiopsis quinghaiensis* YIM 28A4	85.86	79.91	23.5
181.	*Nocardiopsis halotolerans* DSM 44410	*Nocardiopsis terrae* KCTC 19431	86.07	79.91	21.6
182.	*Nocardiopsis ganjiahuensis* DSM 45031	*Nocardiopsis tropica* JCM 10877	86.02	79.90	24
183.	*Nocardiopsis akebiae* HDS12	*Nocardiopsis lambiniae* DSM 44743	86.09	79.89	24.30
184.	*Nocardiopsis lambiniae* DSM 44743	*Nocardiopsis quinghaiensis* YIM 28A4	85.82	79.87	23.7
185.	*Nocardiopsis arvandica* DSM 45278	*Nocardiopsis lambiniae* DSM 44743	85.84	79.83	24.1
186.	*Nocardiopsis prasina* DSM 43845	*Nocardiopsis sinuspersici* UTMC 00102	85.90	79.83	24.2
187.	*Nocardiopsis baichengensis* YIM 90130	*Nocardiopsis potens* DSM 45234	85.74	79.82	23.6
188.	*Nocardiopsis changdeensis* Mg02	*Nocardiopsis tropica* JCM 10877	85.99	79.82	24
189.	*Nocardiopsis dassonvillei subsp. crassaminis* D1	*Nocardiopsis prasina* DSM 43845	86.2	79.82	24.90
190.	*Nocardiopsis halophila* DSM 44494	*Nocardiopsis potens* DSM 45234	85.75	79.82	23.6
191.	*Nocardiopsis alborubida* ATCC 23612	*Nocardiopsis metallicus* JCM 12409	86.53	79.81	25.30
192.	*Nocardiopsis aegyptia* DSM 44442	*Nocardiopsis algeriensis* CECT 8712	85.73	79.79	23.80
193.	*Nocardiopsis deserti* H13	*Nocardiopsis prasina* DSM 43845	86.11	79.77	24.6
194.	*Nocardiopsis algeriensis* CECT 8712	*Nocardiopsis tropica* JCM 10877	85.87	79.73	24.4
195.	*Nocardiopsis ganjiahuensis* DSM 45031	*Nocardiopsis halotolerans* DSM 44410	86.09	79.73	24.5
196.	*Nocardiopsis codii* CT-R113	*Nocardiopsis lambiniae* DSM 44743	85.74	79.72	23.7
197.	*Nocardiopsis dassonvillei* subsp. *dassonvillei* DSM 43111	*Nocardiopsis prasina* DSM 43845	86.15	79.72	24.4
198.	*Nocardiopsis flavescens* CGMCC 4.5723	*Nocardiopsis tropica* JCM 10877	85.87	79.72	23.8

199.	*Nocardiopsis prasina* DSM 43845	*Nocardiopsis quinghaiensis* YIM 28A4	85.93	79.72	23.9
200.	*Nocardiopsis dassonvillei* subsp. *crassaminis* D1	*Nocardiopsis ganjiahuensis* DSM 45031	86.29	79.71	25.00
201.	*Nocardiopsis changdeensis* Mg02	*Nocardiopsis codii* CT-R113	85.88	79.70	23.9
202.	*Nocardiopsis alborubida* ATCC 23612	*Nocardiopsis prasina* DSM 43845	86.08	79.69	24.5
203.	*Nocardiopsis akebiae* HDS12	*Nocardiopsis prasina* DSM 43845	85.99	79.68	24.3
204.	*Nocardiopsis changdeensis* Mg02	*Nocardiopsis halotolerans* DSM 44410	86.07	79.68	24.2
205.	*Nocardiopsis codii* CT-R113	*Nocardiopsis prasina* DSM 43845	85.87	79.66	24
206.	*Nocardiopsis dassonvillei* subsp. *crassaminis* D1	*Nocardiopsis exhalans* JCM11759	86.32	79.64	25.30
207.	*Nocardiopsis alkaliphila* YIM 80379	*Nocardiopsis prasina* DSM 43845	85.88	79.63	24
208.	*Nocardiopsis halotolerans* DSM 44410	*Nocardiopsis lambiniae* DSM 44743	85.86	79.62	22.5
209.	*Nocardiopsis dassonvillei* subsp. dassonvillei DSM 43111	*Nocardiopsis ganjiahuensis* DSM 45031	86.17	79.61	24.50
210.	*Nocardiopsis terrae* KCTC 19431	*Nocardiopsis tropica* JCM 10877	85.95	79.60	24
211.	*Nocardiopsis aegyptia* DSM 44442	*Nocardiopsis terrae* KCTC 19431	86.12	79.59	23.80
212.	*Nocardiopsis dassonvillei* subsp. *crassaminis* D1	*Nocardiopsis metallicus* JCM 12409	86.33	79.59	25.20
213.	*Nocardiopsis alborubida* ATCC 23612	*Nocardiopsis ganjiahuensis* DSM 45031	86.2	79.57	24.70
214.	*Nocardiopsis prasina* DSM 43845	*Nocardiopsis tropica* JCM 10877	85.81	79.57	23.8
215.	*Nocardiopsis arvandica* DSM 45278	*Nocardiopsis ganjiahuensis* DSM 45031	86.18	79.56	24.6
216.	*Nocardiopsis arvandica* DSM 45278	*Nocardiopsis exhalans* JCM11759	86.17	79.52	24.6
217.	*Nocardiopsis dassonvillei* subsp. *dassonvillei* DSM 43111	*Nocardiopsis exhalans* JCM11759	86.22	79.52	24.80
218.	*Nocardiopsis metallicus* JCM 12409	*Nocardiopsis tropica* JCM 10877	85.99	79.52	24
219.	*Nocardiopsis codii* CT-R113	*Nocardiopsis lucentensis* DSM 44048	85.98	79.51	24.6
220.	*Nocardiopsis deserti* H13	*Nocardiopsis ganjiahuensis* DSM 45031	86.24	79.51	24.6
221.	*Nocardiopsis exhalans* JCM11759	*Nocardiopsis halotolerans* DSM 44410	86.05	79.47	24.6
222.	*Nocardiopsis arvandica* DSM 45278	*Nocardiopsis prasina* DSM 43845	85.92	79.45	24.1
223.	*Nocardiopsis dassonvillei* subsp. *dassonvillei* DSM 43111	*Nocardiopsis metallicus* JCM 12409	86.24	79.45	24.60

224.	*Nocardiopsis akebiae* HDS12	*Nocardiopsis ganjiahuensis* DSM 45031	86.2	79.43	24.30
225.	*Nocardiopsis codii* CT-R113	*Nocardiopsis ganjiahuensis* DSM 45031	86.05	79.43	24.2
226.	*Nocardiopsis flavescens* CGMCC 4.5723	*Nocardiopsis halotolerans* DSM 44410	85.80	79.41	23.8
227.	*Nocardiopsis algeriensis* CECT 8712	*Nocardiopsis codii* CT-R113	85.88	79.40	24.4
228.	*Nocardiopsis arvandica* DSM 45278	*Nocardiopsis valliformi*s DSM 45023	86.04	79.40	24.5
229.	*Nocardiopsis dassonvillei* subsp. *crassaminis* D1	*Nocardiopsis valliformi*s DSM 45023	86.28	79.40	25
230.	*Nocardiopsis deserti* H13	Nocardiopsis exhalans JCM11759	86.20	79.40	24.8
231.	*Nocardiopsis exhalans* JCM11759	*Nocardiopsis tropica* JCM 10877	86.01	79.40	24.1
232.	*Nocardiopsis lambiniae* DSM 44743	*Nocardiopsis tropica* JCM 10877	85.78	79.39	23.7
233.	*Nocardiopsis deserti* H13	*Nocardiopsis metallicus* JCM 12409	86.19	79.37	24.8
234.	*Nocardiopsis arvandica* DSM 45278	*Nocardiopsis metallicus* JCM 12409	86.08	79.36	24.4
235.	*Nocardiopsis akebiae* HDS12	*Nocardiopsis exhalans* JCM11759	86.23	79.33	24.60
236.	*Nocardiopsis akebiae* HDS12	*Nocardiopsis metallicus* JCM 12409	86.22	79.29	24.50
237.	*Nocardiopsis sinuspersici* UTMC 00102	*Nocardiopsis valliformi*s DSM 45023	86.01	79.27	24.4
238.	*Nocardiopsis dassonvillei* subsp. *dassonvillei* DSM 43111	*Nocardiopsis valliformi*s DSM 45023	86.17	79.26	24.6
239.	*Nocardiopsis deserti* H13	*Nocardiopsis valliformi*s DSM 45023	86.13	79.25	24.6
240.	*Nocardiopsis halotolerans* DSM 44410	*Nocardiopsis prasina* DSM 43845	86.01	79.22	21.9
241.	*Nocardiopsis codii* CT-R113	*Nocardiopsis exhalans* JCM11759	86.00	79.20	24.2
242.	*Nocardiopsis halotolerans* DSM 44410	*Nocardiopsis metallicus* JCM 12409	86.08	79.20	22
243.	*Nocardiopsis lucentensis* DSM 44048	*Nocardiopsis trehalosi* NBRC 14201	86.00	79.19	24.4
244.	*Nocardiopsis alborubida* ATCC 23612	*Nocardiopsis valliformi*s DSM 45023	86.13	79.18	24.6
245.	*Nocardiopsis aegyptia* DSM 44442	*Nocardiopsis changdeensis* Mg02	85.91	79.17	23.50
246.	*Nocardiopsis codii* CT-R113	*Nocardiopsis metallicus* JCM 12409	86.02	79.14	24.2
247.	*Nocardiopsis codii* CT-R113	*Nocardiopsis valliformi*s DSM 45023	85.95	79.13	24.1
248.	*Nocardiopsis akebiae* HDS12	*Nocardiopsis valliformi*s DSM 45023	86.18	79.12	24.4

249.	*Nocardiopsis quinghaiensis* YIM 28A4	*Nocardiopsis valliform*is DSM 45023	86.02	79.11	24.1
250.	*Nocardiopsis aegyptia* DSM 44442	*Nocardiopsis prasina* DSM 43845	*85.88*	79.02	23.50
251.	*Nocardiopsis aegyptia* DSM 44442	*Nocardiopsis exhalans* JCM11759	85.97	79	24.00
252.	*Nocardiopsis lucentensis* DSM 44048	*Nocardiopsis sinuspersici* UTMC 00102	85.92	79.00	23.6
253.	*Nocardiopsis alba* DSM 43377	*Nocardiopsis dassonvillei* subsp. *dassonvillei* DSM 43111	85.89	78.98	23.70
254.	*Nocardiopsis halotolerans* DSM 44410	*Nocardiopsis valliform*is DSM 45023	86.04	78.98	26.8
255.	*Nocardiopsis ganjiahuensis* DSM 45031	*Nocardiopsis lucentensis* DSM 44048	85.94	78.94	24
256.	*Nocardiopsis tropica* JCM 10877	*Nocardiopsis valliform*is DSM 45023	85.90	78.94	23.9
257.	*Nocardiopsis ganjiahuensis* DSM 45031	*Nocardiopsis lambiniae* DSM 44743	85.77	78.93	23
258.	*Nocardiopsis aegyptia* DSM 44442	*Nocardiopsis lambiniae* DSM 44743	85.72	78.91	23.40
259.	*Nocardiopsis alba* DSM 43377	*Nocardiopsis sinuspersici* UTMC 00102	85.71	78.91	23.4
260.	*Nocardiopsis aegyptia* DSM 44442	*Nocardiopsis metallicus* JCM 12409	85.97	78.9	24.00
261.	*Nocardiopsis aegyptia* DSM 44442	*Nocardiopsis flavescens* CGMCC 4.5723	85.64	78.89	23.20
262.	*Nocardiopsis listeri* NBRC 13360	*Nocardiopsis sinuspersici* UTMC 00102	85.56	78.89	23.1
263.	*Nocardiopsis aegyptia* DSM 44442	*Nocardiopsis ganjiahuensis* DSM 45031	86.09	78.88	24.20
264.	*Nocardiopsis exhalans* JCM11759	*Nocardiopsis lucentensis* DSM 44048	85.85	78.88	23.9
265.	*Nocardiopsis alba* DSM 43377	*Nocardiopsis arvandica* DSM 45278	85.73	78.86	23.40
266.	*Nocardiopsis lambiniae* DSM 44743	*Nocardiopsis terrae* KCTC 19431	85.70	78.86	22.7
267.	*Nocardiopsis changdeensis* Mg02	*Nocardiopsis terrae* KCTC 19431	85.63	78.84	22.7
268.	*Nocardiopsis dassonvillei* subsp. *crassaminis* D1	*Nocardiopsis listeri* NBRC 13360	85.6	78.84	23.70
269.	*Nocardiopsis aegyptia* DSM 44442	*Nocardiopsis valliform*is DSM 45023	85.92	78.79	23.90
270.	*Nocardiopsis exhalans* JCM11759	*Nocardiopsis flavescens* CGMCC 4.5723	85.61	78.79	23
271.	*Nocardiopsis exhalans* JCM11759	*Nocardiopsis lambiniae* DSM 44743	85.68	78.77	23.1
272.	*Nocardiopsis listeri* NBRC 13360	*Nocardiopsis quinghaiensis* YIM 28A4	85.61	78.76	22.8
273.	*Nocardiopsis alborubida* ATCC 23612	*Nocardiopsis listeri* NBRC 13360	85.59	78.72	23.20

274.	*Nocardiopsis algeriensis* CECT 8712	*Nocardiopsis lucentensis* DSM 44048	85.91	78.71	23.8
275.	*Nocardiopsis akebiae* HDS12	*Nocardiopsis alba* DSM 43377	85.84	78.67	23.50
276.	*Nocardiopsis alba* DSM 43377	*Nocardiopsis quinghaiensis* YIM 28A4	85.70	78.67	23.1
277.	*Nocardiopsis deserti* H13	*Nocardiopsis listeri* NBRC 13360	85.59	78.66	23.3
278.	*Nocardiopsis dassonvillei* subsp. *dassonvillei* DSM 43111	*Nocardiopsis listeri* NBRC 13360	85.55	78.64	23.30
279.	*Nocardiopsis alba* DSM 43377	*Nocardiopsis dassonvillei* subsp. *crassaminis* D1	85.89	78.63	24.10
280.	*Nocardiopsis akebiae* HDS12	*Nocardiopsis listeri* NBRC 13360	85.55	78.61	23.20
281.	*Nocardiopsis algeriensis* CECT 8712	*Nocardiopsis terrae* KCTC 19431	85.55	78.56	23.1
282.	*Nocardiopsis arvandica* DSM 45278	*Nocardiopsis listeri* NBRC 13360	85.57	78.56	23.2
283.	*Nocardiopsis alkaliphila* YIM 80379	*Nocardiopsis arvandica* DSM 45278	85.41	78.53	23.1
284.	*Nocardiopsis changdeensis* Mg02	*Nocardiopsis prasina* DSM 43845	85.73	78.50	22.9
285.	*Nocardiopsis flavescens* CGMCC 4.5723	*Nocardiopsis terrae* KCTC 19431	85.50	78.50	22.6
286.	*Nocardiopsis mwathae* DSM 46659	*Nocardiopsis trehalosi* NBRC 14201	85.32	78.49	23.0
287.	*Nocardiopsis changdeensis* Mg02	*Nocardiopsis ganjiahuensis* DSM 45031	85.80	78.47	23.1
288.	*Nocardiopsis changdeensis* Mg02	*Nocardiopsis lucentensis* DSM 44048	85.93	78.45	23.4
289.	*Nocardiopsis lucentensis* DSM 44048	*Nocardiopsis potens* DSM 45234	85.80	78.45	23.6
290.	*Nocardiopsis alba* DSM 43377	*Nocardiopsis alborubida* ATCC 23612	85.8	78.44	23.60
291.	*Nocardiopsis alba* DSM 43377	*Nocardiopsis deserti* H13	85.79	78.44	23.70
292.	*Nocardiopsis lambiniae* DSM 44743	*Nocardiopsis lucentensis* DSM 44048	85.69	78.42	23.2
293.	*Nocardiopsis lucentensis* DSM 44048	*Nocardiopsis metallicus* JCM 12409	85.90	78.41	24
294.	*Nocardiopsis codii* CT-R113	*Nocardiopsis listeri* NBRC 13360	85.47	78.39	23
295.	*Nocardiopsis alba* DSM 43377	*Nocardiopsis tropica* JCM 10877	85.60	78.37	22.9
296.	*Nocardiopsis coralli* HNM0947	*Nocardiopsis dassonvillei* subsp. *dassonvillei* DSM 43111	85.47	78.33	23.2
297.	*Nocardiopsis flavescens* CGMCC 4.5723	*Nocardiopsis prasina* DSM 43845	85.57	78.33	22.7
298.	*Nocardiopsis alkaliphila* YIM 80379	*Nocardiopsis dassonvillei* subsp. *crassaminis* D1	85.54	78.32	23.5

299.	*Nocardiopsis coralli* HNM0947	*Nocardiopsis sinuspersici* UTMC 00102	85.4	78.32	22.90
300.	*Nocardiopsis flavescens* CGMCC 4.5723	*Nocardiopsis lucentensis* DSM 44048	85.69	78.29	23
301.	*Nocardiopsis halotolerans* DSM 44410	*Nocardiopsis listeri* NBRC 13360	85.54	78.29	23.9
302.	*Nocardiopsis alborubida* ATCC 23612	*Nocardiopsis alkaliphila* YIM 80379	85.42	78.28	23.00
303.	*Nocardiopsis lucentensis* DSM 44048	*Nocardiopsis tropica* JCM 10877	85.72	78.27	23.9
304.	*Nocardiopsis alkaliphila* YIM 80379	*Nocardiopsis dassonvillei* subsp. *dassonvillei* DSM 43111	85.49	78.25	23.3
305.	*Nocardiopsis alkaliphila* YIM 80379	*Nocardiopsis sinuspersici* UTMC 00102	85.41	78.25	22.9
306.	*Nocardiopsis akebiae* HDS12	*Nocardiopsis alkaliphila* YIM 80379	85.51	78.24	23.20
307.	*Nocardiopsis alba* DSM 43377	*Nocardiopsis halotolerans* DSM 44410	85.78	78.24	23.50
308.	*Nocardiopsis lambiniae* DSM 44743	*Nocardiopsis prasina* DSM 43845	85.63	78.23	22.6
309.	*Nocardiopsis listeri* NBRC 13360	*Nocardiopsis tropica* JCM 10877	85.46	78.22	22.8
310.	*Nocardiopsis alba* DSM 43377	*Nocardiopsis algeriensis* CECT 8712	85.6	78.2	22.40
311.	*Nocardiopsis coralli* HNM0947	*Nocardiopsis dassonvillei* subsp. *crassaminis* D1	85.52	78.20	23.6
312.	*Nocardiopsis changdeensis* Mg02	*Nocardiopsis metallicus* JCM 12409	85.75	78.19	23.1
313.	*Nocardiopsis coralli* HNM0947	*Nocardiopsis quinghaiensis* YIM 28A4	85.35	78.18	22.60
314.	*Nocardiopsis changdeensis* Mg02	*Nocardiopsis valliformi*s DSM 45023	85.65	78.16	23.1
315.	*Nocardiopsis alba* DSM 43377	*Nocardiopsis codii* CT-R113	85.7	78.15	23.00
316.	*Nocardiopsis flavescens* CGMCC 4.5723	*Nocardiopsis ganjiahuensis* DSM 45031	85.64	78.15	22.9
317.	*Nocardiopsis flavescens* CGMCC 4.5723	*Nocardiopsis metallicus* JCM 12409	85.65	78.15	23
318.	*Nocardiopsis lambiniae* DSM 44743	*Nocardiopsis metallicus* JCM 12409	85.62	78.15	22.9
319.	*Nocardiopsis algeriensis* CECT 8712	*Nocardiopsis ganjiahuensis* DSM 45031	85.72	78.14	23.6
320.	*Nocardiopsis algeriensis* CECT 8712	*Nocardiopsis prasina* DSM 43845	85.57	78.14	23.1
321.	*Nocardiopsis dassonvillei* subsp. *crassaminis* D1	*Nocardiopsis xinjiangensis* YIM 90004	85.31	78.14	23.1
322.	*Nocardiopsis dassonvillei* subsp. *crassaminis* D1	*Nocardiopsis litoralis* KCTC 19473	85.29	78.13	23.10
323.	*Nocardiopsis potens* DSM 45234	*Nocardiopsis trehalosi* NBRC 14201	85.10	78.13	22.1

324.	*Nocardiopsis alkaliphila* YIM 80379	*Nocardiopsis quinghaiensis* YIM 28A4	85.36	78.12	22.7
325.	*Nocardiopsis changdeensis* Mg02	*Nocardiopsis exhalans* JCM11759	85.75	78.10	23.2
326.	*Nocardiopsis gilva* YIM 90087	*Nocardiopsis trehalosi* NBRC 14201	85.23	78.09	22.9
327.	*Nocardiopsis aegyptia* DSM 44442	*Nocardiopsis alba* DSM 43377	85.7	78.08	23.10
328.	*Nocardiopsis algeriensis* CECT 8712	*Nocardiopsis exhalans* JCM11759	85.72	78.08	23.6
329.	*Nocardiopsis algeriensis* CECT 8712	*Nocardiopsis valliformi*s DSM 45023	85.59	78.08	23.4
330.	*Nocardiopsis algeriensis* CECT 8712	*Nocardiopsis metallicus* JCM 12409	85.73	78.07	23.6
331.	*Nocardiopsis lambiniae* DSM 44743	*Nocardiopsis valliformi*s DSM 45023	85.71	78.02	23.2
332.	*Nocardiopsis rhodophaea* JCM 15313	*Nocardiopsis trehalosi* NBRC 14201	85.24	78.02	22.5
333.	*Nocardiopsis deserti* H13	*Nocardiopsis xinjiangensis* YIM 90004	85.16	78.00	22.7
334.	*Nocardiopsis aegyptia* DSM 44442	*Nocardiopsis listeri* NBRC 13360	85.45	77.99	22.70
335.	*Nocardiopsis alba* DSM 43377	*Nocardiopsis changdeensis* Mg02	85.65	77.98	22.50
336.	*Nocardiopsis alkaliphila* YIM 80379	*Nocardiopsis deserti* H13	85.41	77.98	23.1
337.	*Nocardiopsis salina* YIM 90010	*Nocardiopsis sinuspersici* UTMC 00102	85.31	77.96	22.6
338.	*Nocardiopsis deserti* H13	*Nocardiopsis litoralis* KCTC 19473	85.12	77.95	22.6
339.	*Nocardiopsis akebiae* HDS12	*Nocardiopsis litoralis* KCTC 19473	85.12	77.93	22.60
340.	*Nocardiopsis alkaliphila* YIM 80379	*Nocardiopsis halotolerans* DSM 44410	85.52	77.92	23
341.	*Nocardiopsis dassonvillei* subsp. *dassonvillei* DSM 43111	*Nocardiopsis xinjiangensis* YIM 90004	85.26	77.92	22.7
342.	*Nocardiopsis alborubida* ATCC 23612	*Nocardiopsis xinjiangensis* YIM 90004	85.17	77.91	22.7
343.	*Nocardiopsis dassonvillei* subsp. *crassaminis* D1	*Nocardiopsis kunsanensis* DSM 44524	85.27	77.91	23.00
344.	*Nocardiopsis coralli* HNM0947	*Nocardiopsis halotolerans* DSM 44410	85.47	77.86	23.1
345.	*Nocardiopsis dassonvillei* subsp. *dassonville*i DSM 43111	*Nocardiopsis litoralis* KCTC 19473	85.29	77.86	22.60
346.	*Nocardiopsis alba* DSM 43377	*Nocardiopsis flavescens* CGMCC 4.5723	85.44	77.85	22.10
347.	*Nocardiopsis coralli* HNM0947	*Nocardiopsis deserti* H13	85.42	77.85	23.1
348.	*Nocardiopsis coralli* HNM0947	*Nocardiopsis terrae* KCTC 19431	85.34	77.85	22.30

349.	*Nocardiopsis flavescens* CGMCC 4.5723	*Nocardiopsis valliformi*s DSM 45023	85.55	77.84	22.8
350.	*Nocardiopsis litoralis* KCTC 19473	*Nocardiopsis sinuspersici* UTMC 00102	85.13	77.84	22.3
351.	*Nocardiopsis sinuspersici* UTMC 00102	*Nocardiopsis xinjiangensis* YIM 90004	85.21	77.84	22.5
352.	*Nocardiopsis akebiae* HDS12	*Nocardiopsis coralli* HNM0947	85.48	77.82	23.00
353.	*Nocardiopsis akebiae* HDS12	*Nocardiopsis xinjiangensis* YIM 90004	85.22	77.81	22.7
354.	*Nocardiopsis alborubida* ATCC 23612	*Nocardiopsis litoralis* KCTC 19473	85.13	77.81	22.70
355.	*Nocardiopsis arvandica* DSM 45278	*Nocardiopsis xinjiangensis* YIM 90004	85.24	77.80	22.5
356.	*Nocardiopsis changdeensis* Mg02	*Nocardiopsis listeri* NBRC 13360	85.32	77.80	22.1
357.	*Nocardiopsis alborubida* ATCC 23612	*Nocardiopsis dassonvillei* subsp. *crassaminis* D1	85.5	77.78	23.20
358.	*Nocardiopsis arvandica* DSM 45278	*Nocardiopsis litoralis* KCTC 19473	85.07	77.78	22.3
359.	*Nocardiopsis dassonvillei* subsp. *crassaminis* D1	*Nocardiopsis salina* YIM 90010	85.38	77.78	23.2
360.	*Nocardiopsis alba* DSM 43377	*Nocardiopsis lucentensis* DSM 44048	85.66	77.76	23.00
361.	*Nocardiopsis ganjiahuensis* DSM 45031	*Nocardiopsis xinjiangensis* YIM 90004	85.00	77.74	22.3
362.	*Nocardiopsis dassonvillei* subsp. *dassonvillei* DSM 43111	*Nocardiopsis kunsanensis* DSM 44524	85.26	77.73	22.50
363.	*Nocardiopsis kunsanensis* DSM 44524	*Nocardiopsis sinuspersici* UTMC 00102	85.12	77.73	22.2
364.	*Nocardiopsis listeri* NBRC 13360	*Nocardiopsis lucentensis* DSM 44048	85.51	77.73	22.9
365.	*Nocardiopsis alba* DSM 43377	*Nocardiopsis lambiniae* DSM 44743	85.57	77.72	22.40
366.	*Nocardiopsis coralli* HNM0947	*Nocardiopsis prasina* DSM 43845	85.14	77.72	22.30
367.	*Nocardiopsis halotolerans* DSM 44410	*Nocardiopsis xinjiangensis* YIM 90004	85.31	77.72	24.6
368.	*Nocardiopsis akebiae* HDS12	*Nocardiopsis kunsanensis* DSM 44524	85.11	77.7	22.50
369.	*Nocardiopsis alborubida* ATCC 23612	*Nocardiopsis salina* YIM 90010	85.36	77.69	22.9
370.	*Nocardiopsis halotolerans* DSM 44410	*Nocardiopsis litoralis* KCTC 19473	85.40	77.69	23.2
371.	*Nocardiopsis coralli* HNM0947	*Nocardiopsis tropica* JCM 10877	85.21	77.68	22.50
372.	*Nocardiopsis dassonvillei* subsp. *dassonvillei* DSM 43111	*Nocardiopsis salina* YIM 90010	85.34	77.68	22.8
373.	*Nocardiopsis exhalans* JCM11759	*Nocardiopsis litoralis* KCTC 19473	85.14	77.68	22.5

374.	*Nocardiopsis quinghaiensis* YIM 28A4	*Nocardiopsis xinjiangensis* YIM 90004	85.17	77.68	22.3
375.	*Nocardiopsis alborubida* ATCC 23612	*Nocardiopsis kunsanensis* DSM 44524	85.13	77.67	22.70
376.	*Nocardiopsis arvandica* DSM 45278	*Nocardiopsis coralli* HNM0947	85.45	77.67	22.9
377.	*Nocardiopsis alkaliphila* YIM 80379	*Nocardiopsis codii* CT-R113	85.33	77.66	22.5
378.	*Nocardiopsis alkaliphila* YIM 80379	*Nocardiopsis tropica* JCM 10877	85.25	77.66	22.6
379.	*Nocardiopsis coralli* HNM0947	*Nocardiopsis exhalans* JCM11759	85.44	77.64	22.8
380.	*Nocardiopsis deserti* H13	*Nocardiopsis kunsanensis* DSM 44524	85.14	77.64	22.7
381.	*Nocardiopsis aegyptia* DSM 44442	*Nocardiopsis alkaliphila* YIM 80379	85.25	77.63	22.60
382.	*Nocardiopsis flavescens* CGMCC 4.5723	*Nocardiopsis listeri* NBRC 13360	85.19	77.63	22
383.	*Nocardiopsis metallicus* JCM 12409	*Nocardiopsis xinjiangensis* YIM 90004	85.05	77.63	22.4
384.	*Nocardiopsis exhalans* JCM11759	*Nocardiopsis xinjiangensis* YIM 90004	85.10	77.62	22.4
385.	*Nocardiopsis deserti* H13	*Nocardiopsis salina* YIM 90010	85.30	77.61	22.8
386.	*Nocardiopsis lambiniae* DSM 44743	*Nocardiopsis listeri* NBRC 13360	85.27	77.59	22
387.	*Nocardiopsis litoralis* KCTC 19473	*Nocardiopsis quinghaiensis* YIM 28A4	85.15	77.59	22.2
388.	*Nocardiopsis akebiae* HDS12	*Nocardiopsis salina* YIM 90010	85.25	77.57	22.8
389.	*Nocardiopsis coralli* HNM0947	*Nocardiopsis flavescens* CGMCC 4.5723	85.11	77.57	21.8
390.	*Nocardiopsis metallicus* JCM 12409	*Nocardiopsis salina* YIM 90010	85.40	77.57	22.9
391.	*Nocardiopsis coralli* HNM0947	*Nocardiopsis metallicus* JCM 12409	85.49	77.56	23
392.	*Nocardiopsis kunsanensis* DSM 44524	*Nocardiopsis quinghaiensis* YIM 28A4	85.13	77.56	22.1
393.	*Nocardiopsis baichengensis* YIM 90130	*Nocardiopsis trehalosi* NBRC 14201	84.76	77.53	21.6
394.	*Nocardiopsis ganjiahuensis* DSM 45031	*Nocardiopsis litoralis* KCTC 19473	85.00	77.52	22.1
395.	*Nocardiopsis mangrovi* CGMCC 4.7119	*Nocardiopsis mwathae* DSM 46659	85.20	77.52	22.6
396.	*Nocardiopsis arvandica* DSM 45278	*Nocardiopsis salina* YIM 90010	85.30	77.50	22.6
397.	*Nocardiopsis halotolerans* DSM 44410	*Nocardiopsis salina* YIM 90010	85.27	77.50	21.9
398.	*Nocardiopsis coralli* HNM0947	*Nocardiopsis ganjiahuensis* DSM 45031	85.27	77.49	22.6

399.	*Nocardiopsis ganjiahuensis* DSM 45031	*Nocardiopsis salina* YIM 90010	85.21	77.49	22.2
400.	*Nocardiopsis halophila* DSM 44494	*Nocardiopsis trehalosi* NBRC 14201	84.77	77.49	21
401.	*Nocardiopsis arvandica* DSM 45278	*Nocardiopsis kunsanensis* DSM 44524	85.09	77.48	22.3
402.	*Nocardiopsis codii* CT-R113	*Nocardiopsis coralli* HNM0947	85.19	77.48	22.6
403.	*Nocardiopsis valliformi*s DSM 45023	*Nocardiopsis xinjiangensis* YIM 90004	85.00	77.47	22.4
404.	*Nocardiopsis salina* YIM 90010	*Nocardiopsis terrae* KCTC 19431	85.23	77.46	22
405.	*Nocardiopsis algeriensis* CECT 8712	*Nocardiopsis listeri* NBRC 13360	85.32	77.44	22.2
406.	*Nocardiopsis tropica* JCM 10877	*Nocardiopsis xinjiangensis* YIM 90004	84.93	77.44	22.1
407.	*Nocardiopsis codii* CT-R113	*Nocardiopsis xinjiangensis* YIM 90004	84.89	77.43	22.2
408.	*Nocardiopsis aegyptia* DSM 44442	*Nocardiopsis litoralis* KCTC 19473	85.09	77.4	22.30
409.	*Nocardiopsis quinghaiensis* YIM 28A4	*Nocardiopsis salina* YIM 90010	85.28	77.40	22.3
410.	*Nocardiopsis exhalans* JCM11759	*Nocardiopsis kunsanensis* DSM 44524	85.13	77.39	22.5
411.	*Nocardiopsis halotolerans* DSM 44410	*Nocardiopsis kunsanensis* DSM 44524	85.30	77.39	20.4
412.	*Nocardiopsis coralli* HNM0947	*Nocardiopsis lucentensis* DSM 44048	85.37	77.36	22.8
413.	*Nocardiopsis ganjiahuensis* DSM 45031	*Nocardiopsis kunsanensis* DSM 44524	84.99	77.36	22.1
414.	*Nocardiopsis alkaliphila* YIM 80379	*Nocardiopsis lucentensis* DSM 44048	85.24	77.35	22.6
415.	*Nocardiopsis terrae* KCTC 19431	*Nocardiopsis xinjiangensis* YIM 90004	85.12	77.34	22
416.	*Nocardiopsis alkaliphila* YIM 80379	*Nocardiopsis changdeensis* Mg02	85.32	77.32	22
417.	*Nocardiopsis aegyptia* DSM 44442	*Nocardiopsis xinjiangensis* YIM 90004	84.98	77.31	22.30
418.	*Nocardiopsis exhalans* JCM11759	*Nocardiopsis salina* YIM 90010	85.21	77.31	22.7
419.	*Nocardiopsis codii* CT-R113	*Nocardiopsis litoralis* KCTC 19473	84.96	77.28	22.1
420.	*Nocardiopsis salina* YIM 90010	*Nocardiopsis tropica* JCM 10877	85.07	77.28	22.2
421.	*Nocardiopsis aegyptia* DSM 44442	*Nocardiopsis coralli* HNM0947	85.25	77.25	22.50
422.	*Nocardiopsis algeriensis* CECT 8712	*Nocardiopsis alkaliphila* YIM 80379	85.22	77.25	22.20
423.	*Nocardiopsis alkaliphila* YIM 80379	*Nocardiopsis lambiniae* DSM 44743	85.19	77.24	22

424.	*Nocardiopsis coralli* HNM0947	*Nocardiopsis valliformi*s DSM 45023	85.32	77.24	22.50
425.	*Nocardiopsis aegyptia* DSM 44442	*Nocardiopsis kunsanensis* DSM 44524	85.08	77.22	22.30
426.	*Nocardiopsis mwathae* DSM 46659	*Nocardiopsis sediminis* TBRC 1826	85.25	77.22	22.6
427.	*Nocardiopsis alkaliphila* YIM 80379	*Nocardiopsis flavescens* CGMCC 4.5723	85.25	77.21	21.9
428.	*Nocardiopsis prasina* DSM 43845	*Nocardiopsis xinjiangensis* YIM 90004	84.98	77.20	21.7
429.	*Nocardiopsis aegyptia* DSM 44442	*Nocardiopsis salina* YIM 90010	85.27	77.16	22.50
430.	*Nocardiopsis changdeensis* Mg02	*Nocardiopsis coralli* HNM0947	85.24	77.15	22.2
431.	*Nocardiopsis codii* CT-R113	*Nocardiopsis salina* YIM 90010	84.99	77.15	22.4
432.	*Nocardiopsis gilva* YIM 90087	*Nocardiopsis sediminis* TBRC 1826	85.13	77.14	22.7
433.	*Nocardiopsis codii* CT-R113	*Nocardiopsis kunsanensis* DSM 44524	84.94	77.12	22.1
434.	*Nocardiopsis litoralis* KCTC 19473	*Nocardiopsis terrae* KCTC 19431	85.13	77.12	21.9
435.	*Nocardiopsis mwathae* DSM 46659	*Nocardiopsis potens* DSM 45234	85.06	77.12	22.3
436.	*Nocardiopsis changdeensis* Mg02	*Nocardiopsis xinjiangensis* YIM 90004	84.94	77.10	22
437.	*Nocardiopsis kunsanensis* DSM 44524	*Nocardiopsis terrae* KCTC 19431	85.09	77.09	21.9
438.	*Nocardiopsis salina* YIM 90010	*Nocardiopsis valliformi*s DSM 45023	85.19	77.08	22.4
439.	*Nocardiopsis coralli* HNM0947	*Nocardiopsis lambiniae* DSM 44743	85.01	77.06	21.8
440.	*Nocardiopsis litoralis* KCTC 19473	*Nocardiopsis tropica* JCM 10877	84.98	77.06	21.9
441.	*Nocardiopsis prasina* DSM 43845	*Nocardiopsis salina* YIM 90010	85.12	77.04	22.1
442.	*Nocardiopsis rhodophaea* JCM 15313	*Nocardiopsis sediminis* TBRC 1826	85.15	77.04	22.6
443.	*Nocardiopsis coralli* HNM0947	*Nocardiopsis listeri* NBRC 13360	85.10	77.03	21.9
444.	*Nocardiopsis mangrovi* CGMCC 4.7119	*Nocardiopsis rhodophaea* JCM 15313	85.14	77.03	22.5
445.	*Nocardiopsis algeriensis* CECT 8712	*Nocardiopsis coralli* HNM0947	85.16	77.01	22.2
446.	*Nocardiopsis algeriensis* CECT 8712	*Nocardiopsis litoralis* KCTC 19473	85.05	77.00	21.5
447.	*Nocardiopsis changdeensis* Mg02	*Nocardiopsis litoralis* KCTC 19473	85.01	76.98	21.8
448.	*Nocardiopsis litoralis* KCTC 19473	*Nocardiopsis prasina* DSM 43845	85.05	76.98	21.8

449.	*Nocardiopsis kunsanensis* DSM 44524	*Nocardiopsis tropica* JCM 10877	84.95	76.95	22
450.	*Nocardiopsis listeri* NBRC 13360	*Nocardiopsis litoralis* KCTC 19473	85.01	76.94	21.5
451.	*Nocardiopsis gilva* YIM 90087	*Nocardiopsis mangrovi* CGMCC 4.7119	85.15	76.93	22.8
452.	*Nocardiopsis listeri* NBRC 13360	*Nocardiopsis xinjiangensis* YIM 90004	85.09	76.93	21.5
453.	*Nocardiopsis lucentensis* DSM 44048	*Nocardiopsis valliformi*s DSM 45023	85.16	76.93	22.3
454.	*Nocardiopsis kunsanensis* DSM 44524	*Nocardiopsis prasina* DSM 43845	85.05	76.91	21.8
455.	*Nocardiopsis changdeensis* Mg02	*Nocardiopsis salina* YIM 90010	85.12	76.89	22
456.	*Nocardiopsis alba* DSM 43377	*Nocardiopsis xinjiangensis* YIM 90004	85.02	76.86	21.5
457.	*Nocardiopsis algeriensis* CECT 8712	*Nocardiopsis xinjiangensis* YIM 90004	84.98	76.86	21.7
458.	*Nocardiopsis flavescens* CGMCC 4.5723	*Nocardiopsis xinjiangensis* YIM 90004	84.82	76.85	21.5
459.	*Nocardiopsis alba* DSM 43377	*Nocardiopsis litoralis* KCTC 19473	85.08	76.84	21.40
460.	*Nocardiopsis changdeensis* Mg02	*Nocardiopsis kunsanensis* DSM 44524	85.03	76.84	21.8
461.	*Nocardiopsis flavescens* CGMCC 4.5723	*Nocardiopsis litoralis* KCTC 19473	84.92	76.84	21.5
462.	*Nocardiopsis litoralis* KCTC 19473	*Nocardiopsis metallicus* JCM 12409	85.15	76.83	22.5
463.	*Nocardiopsis algeriensis* CECT 8712	*Nocardiopsis salina* YIM 90010	85.20	76.80	22
464.	*Nocardiopsis gilva* YIM 90087	*Nocardiopsis potens* DSM 45234	85.09	76.79	22.4
465.	*Nocardiopsis litoralis* KCTC 19473	*Nocardiopsis lucentensis* DSM 44048	85.17	76.79	22.40
466.	*Nocardiopsis lucentensis* DSM 44048	*Nocardiopsis rhodophaea* JCM 15313	85.25	76.77	22.4
467.	*Nocardiopsis flavescens* CGMCC 4.5723	*Nocardiopsis salina* YIM 90010	85.04	76.75	21.7
468.	*Nocardiopsis litoralis* KCTC 19473	*Nocardiopsis valliformi*s DSM 45023	85.18	76.74	22.5
469.	*Nocardiopsis algeriensis* CECT 8712	*Nocardiopsis kunsanensis* DSM 44524	85.07	76.73	21.5
470.	*Nocardiopsis kunsanensis* DSM 44524	*Nocardiopsis lucentensis* DSM 44048	85.22	76.73	22.5
471.	*Nocardiopsis lambiniae* DSM 44743	*Nocardiopsis litoralis* KCTC 19473	84.87	76.72	21.6
472.	*Nocardiopsis kunsanensis* DSM 44524	*Nocardiopsis listeri* NBRC 13360	85.00	76.71	21.5
473.	*Nocardiopsis flavescens* CGMCC 4.5723	*Nocardiopsis kunsanensis* DSM 44524	84.90	76.69	21.5

474.	*Nocardiopsis alba* DSM 43377	*Nocardiopsis kunsanensis* DSM 44524	85.03	76.68	21.40
475.	*Nocardiopsis lambiniae* DSM 44743	*Nocardiopsis xinjiangensis* YIM 90004	84.94	76.68	21.5
476.	*Nocardiopsis mangrovi* CGMCC 4.7119	*Nocardiopsis potens* DSM 45234	84.89	76.64	22
477.	*Nocardiopsis alba* DSM 43377	*Nocardiopsis salina* YIM 90010	85.24	76.62	21.6
478.	*Nocardiopsis gilva* YIM 90087	*Nocardiopsis halophila* DSM 44494	84.74	76.61	21.9
479.	*Nocardiopsis kunsanensis* DSM 44524	*Nocardiopsis metallicus* JCM 12409	85.10	76.61	22.4
480.	*Nocardiopsis lambiniae* DSM 44743	*Nocardiopsis salina* YIM 90010	85.04	76.58	21.7
481.	*Nocardiopsis alba* DSM 43377	*Nocardiopsis coralli* HNM0947	85.25	76.56	22.00
482.	*Nocardiopsis listeri* NBRC 13360	*Nocardiopsis salina* YIM 90010	85.05	76.55	21.5
483.	*Nocardiopsis kunsanensis* DSM 44524	*Nocardiopsis valliformi*s DSM 45023	85.08	76.54	22.3
484.	*Nocardiopsis halophila* DSM 44494	*Nocardiopsis mwathae* DSM 46659	84.83	76.51	22.1
485.	*Nocardiopsis potens* DSM 45234	*Nocardiopsis rhodophaea* JCM 15313	84.94	76.48	22.2
486.	*Nocardiopsis potens* DSM 45234	*Nocardiopsis sediminis* TBRC 1826	84.82	76.48	21.8
487.	*Nocardiopsis kunsanensis* DSM 44524	*Nocardiopsis lambiniae* DSM 44743	84.91	76.47	21.7
488.	*Nocardiopsis deserti* H13	*Nocardiopsis trehalosi* NBRC 14201	84.78	76.46	22
489.	*Nocardiopsis baichengensis* YIM 90130	*Nocardiopsis mwathae* DSM 46659	84.79	76.45	22.1
490.	*Nocardiopsis dassonvillei* subsp. *crassaminis* D1	*Nocardiopsis trehalosi* NBRC 14201	84.78	76.42	22.1
491.	*Nocardiopsis dassonvillei* subsp. *dassonvillei* DSM 43111	*Nocardiopsis trehalosi* NBRC 14201	84.72	76.40	21.8
492.	*Nocardiopsis alkaliphila* YIM 80379	*Nocardiopsis xinjiangensis* YIM 90004	84.84	76.38	21.3
493.	*Nocardiopsis alborubida* ATCC 23612	*Nocardiopsis trehalosi* NBRC 14201	84.74	76.37	21.7
494.	*Nocardiopsis alkaliphila* YIM 80379	*Nocardiopsis litoralis* KCTC 19473	84.89	76.37	21.5
495.	*Nocardiopsis akebiae* HDS12	*Nocardiopsis trehalosi* NBRC 14201	84.68	76.34	21.8
496.	*Nocardiopsis alkaliphila* YIM 80379	*Nocardiopsis kunsanensis* DSM 44524	84.91	76.23	21.4
497.	*Nocardiopsis sinuspersici* UTMC 00102	*Nocardiopsis trehalosi* NBRC 14201	84.62	76.16	21.5
498.	*Nocardiopsis arvandica* DSM 45278	*Nocardiopsis trehalosi* NBRC 14201	84.61	76.12	21.6

499.	*Nocardiopsis baichengensis* YIM 90130	*Nocardiopsis gilva* YIM 90087	84.76	76.12	21.9
500.	*Nocardiopsis changdeensis* Mg02	*Nocardiopsis trehalosi* NBRC 14201	84.69	76.08	21.2
501.	*Nocardiopsis aegyptia* DSM 44442	*Nocardiopsis trehalosi* NBRC 14201	84.64	76.07	21.50
502.	*Nocardiopsis halophila* DSM 44494	*Nocardiopsis sediminis* TBRC 1826	84.76	76.06	21.6
503.	*Nocardiopsis alkaliphila* YIM 80379	*Nocardiopsis coralli* HNM0947	84.92	76.04	21.6
504.	*Nocardiopsis baichengensis* YIM 90130	*Nocardiopsis sediminis* TBRC 1826	84.76	76.04	21.6
505.	*Nocardiopsis halophila* DSM 44494	*Nocardiopsis rhodophaea* JCM 15313	84.72	76.04	21.9
506.	*Nocardiopsis alkaliphila* YIM 80379	*Nocardiopsis salina* YIM 90010	84.91	76.01	21.4
507.	*Nocardiopsis flavescens* CGMCC 4.5723	*Nocardiopsis trehalosi* NBRC 14201	84.69	75.97	21.2
508.	*Nocardiopsis baichengensis* YIM 90130	*Nocardiopsis mangrovi* CGMCC 4.7119	84.72	75.95	21.8
509.	*Nocardiopsis quinghaiensis* YIM 28A4	*Nocardiopsis trehalosi* NBRC 14201	84.66	75.94	21.1
510.	*Nocardiopsis halophila* DSM 44494	*Nocardiopsis mangrovi* CGMCC 4.7119	84.73	75.93	21.8
511.	*Nocardiopsis baichengensis* YIM 90130	*Nocardiopsis rhodophaea* JCM 15313	84.75	75.88	22
512.	*Nocardiopsis halotolerans* DSM 44410	*Nocardiopsis trehalosi* NBRC 14201	84.72	75.86	24.3
513.	*Nocardiopsis algeriensis* CECT 8712	*Nocardiopsis trehalosi* NBRC 14201	84.74	75.83	21.4
514.	*Nocardiopsis codii* CT-R113	*Nocardiopsis trehalosi* NBRC 14201	84.42	75.78	21.4
515.	*Nocardiopsis dassonvillei* subsp. *crassaminis* D1	*Nocardiopsis halophila* DSM 44494	84.57	75.65	21.60
516.	*Nocardiopsis dassonvillei* subsp. *crassaminis* D1	*Nocardiopsis potens* DSM 45234	84.91	75.62	22.10
517.	*Nocardiopsis ganjiahuensis* DSM 45031	*Nocardiopsis trehalosi* NBRC 14201	84.38	75.62	21.3
518.	*Nocardiopsis prasina* DSM 43845	*Nocardiopsis trehalosi* NBRC 14201	84.46	75.62	21.5
519.	*Nocardiopsis alborubida* ATCC 23612	*Nocardiopsis potens* DSM 45234	84.97	75.61	22
520.	*Nocardiopsis deserti* H13	*Nocardiopsis potens* DSM 45234	84.79	75.60	22
521.	*Nocardiopsis dassonvillei* subsp. *dassonvillei* DSM 43111	*Nocardiopsis halophila* DSM 44494	84.58	75.58	21.40
522.	*Nocardiopsis dassonvillei* subsp. *dassonvillei* DSM 43111	*Nocardiopsis potens* DSM 45234	84.89	75.57	21.9
523.	*Nocardiopsis dassonvillei* subsp. *crassaminis* D1	*Nocardiopsis mwathae* DSM 46659	84.77	75.56	22.20

524.	*Nocardiopsis changdeensis* Mg02	*Nocardiopsis potens* DSM 45234	84.81	75.54	21.5
525.	*Nocardiopsis alborubida* ATCC 23612	*Nocardiopsis halophila* DSM 44494	84.55	75.53	21.40
526.	*Nocardiopsis deserti* H13	*Nocardiopsis halophila* DSM 44494	84.56	75.53	21.4
527.	*Nocardiopsis lambiniae* DSM 44743	*Nocardiopsis trehalosi* NBRC 14201	84.45	75.52	21.3
528.	*Nocardiopsis akebiae* HDS12	*Nocardiopsis potens* DSM 45234	84.92	75.49	22
529.	*Nocardiopsis akebiae* HDS12	*Nocardiopsis baichengensis* YIM 90130	84.55	75.48	21.50
530.	*Nocardiopsis lucentensis* DSM 44048	*Nocardiopsis terrae* KCTC 19431	84.74	75.48	21.7
531.	*Nocardiopsis arvandica* DSM 45278	*Nocardiopsis halophila* DSM 44494	84.48	75.47	21.4
532.	*Nocardiopsis arvandica* DSM 45278	*Nocardiopsis potens* DSM 45234	84.72	75.46	21.6
533.	*Nocardiopsis akebiae* HDS12	*Nocardiopsis halophila* DSM 44494	84.52	75.45	21.50
534.	*Nocardiopsis potens* DSM 45234	*Nocardiopsis sinuspersici* UTMC 00102	84.74	75.44	21.7
535.	*Nocardiopsis alborubida* ATCC 23612	*Nocardiopsis baichengensis* YIM 90130	84.54	75.38	21.30
536.	*Nocardiopsis arvandica* DSM 45278	*Nocardiopsis baichengensis* YIM 90130	84.47	75.38	21.3
537.	*Nocardiopsis exhalans* JCM11759	*Nocardiopsis trehalosi* NBRC 14201	84.43	75.37	21.6
538.	*Nocardiopsis alborubida* ATCC 23612	*Nocardiopsis coralli* HNM0947	84.87	75.36	22.30
539.	*Nocardiopsis dassonvillei* subsp. *crassaminis* D1	*Nocardiopsis sediminis* TBRC 1826	84.70	75.35	22.1
540.	*Nocardiopsis akebiae* HDS12	*Nocardiopsis mwathae* DSM 46659	84.85	75.34	22.00
541.	*Nocardiopsis changdeensis* Mg02	*Nocardiopsis halophila* DSM 44494	84.56	75.34	21.1
542.	*Nocardiopsis dassonvillei* subsp. *dassonville*i DSM 43111	*Nocardiopsis mwathae* DSM 46659	84.79	75.34	22.10
543.	*Nocardiopsis arvandica* DSM 45278	*Nocardiopsis mwathae* DSM 46659	84.74	75.33	22
544.	*Nocardiopsis aegyptia* DSM 44442	*Nocardiopsis halophila* DSM 44494	84.46	75.31	21.30
545.	*Nocardiopsis dassonvillei* subsp. *dassonvillei* DSM 43111	*Nocardiopsis sediminis* TBRC 1826	84.66	75.31	21.7
546.	*Nocardiopsis deserti* H13	*Nocardiopsis mwathae* DSM 46659	84.66	75.31	22.2
547.	*Nocardiopsis aegyptia* DSM 44442	*Nocardiopsis mwathae* DSM 46659	84.61	75.3	22.20
548.	*Nocardiopsis metallicus* JCM 12409	*Nocardiopsis trehalosi* NBRC 14201	84.35	75.30	21.3

549.	*Nocardiopsis mangrovi* CGMCC 4.7119	*Nocardiopsis sinuspersici* UTMC 00102	84.56	75.29	21.9
550.	*Nocardiopsis aegyptia* DSM 44442	*Nocardiopsis potens* DSM 45234	84.6	75.28	21.70
551.	*Nocardiopsis flavescens* CGMCC 4.5723	*Nocardiopsis halophila* DSM 44494	84.42	75.28	20.9
552.	*Nocardiopsis changdeensis* Mg02	*Nocardiopsis mwathae* DSM 46659	84.79	75.26	21.9
553.	*Nocardiopsis dassonvillei* subsp. *crassaminis* D1	*Nocardiopsis mangrovi* CGMCC 4.7119	84.73	75.25	22.30
554.	*Nocardiopsis terrae* KCTC 19431	*Nocardiopsis trehalosi* NBRC 14201	84.42	75.25	21.1
555.	*Nocardiopsis alborubida* ATCC 23612	*Nocardiopsis mwathae* DSM 46659	84.72	75.24	22.00
556.	*Nocardiopsis aegyptia* DSM 44442	*Nocardiopsis baichengensis* YIM 90130	84.45	75.22	21.30
557.	*Nocardiopsis flavescens* CGMCC 4.5723	*Nocardiopsis potens* DSM 45234	84.75	75.21	21.3
558.	*Nocardiopsis alborubida* ATCC 23612	*Nocardiopsis sediminis* TBRC 1826	84.63	75.17	21.8
559.	*Nocardiopsis deserti* H13	*Nocardiopsis sediminis* TBRC 1826	84.68	75.17	21.7
560.	*Nocardiopsis halotolerans* DSM 44410	*Nocardiopsis potens* DSM 45234	84.81	75.16	22
561.	*Nocardiopsis mwathae* DSM 46659	*Nocardiopsis sinuspersici* UTMC 00102	84.67	75.16	22.0
562.	*Nocardiopsis akebiae* HDS12	*Nocardiopsis sediminis* TBRC 1826	84.66	75.15	21.7
563.	*Nocardiopsis sediminis* TBRC 1826	*Nocardiopsis sinuspersici* UTMC 00102	84.59	75.15	21.6
564.	*Nocardiopsis halotolerans* DSM 44410	*Nocardiopsis mwathae* DSM 46659	84.70	75.14	24.7
565.	*Nocardiopsis halophila* DSM 44494	*Nocardiopsis sinuspersici* UTMC 00102	84.44	75.12	21.5
566.	*Nocardiopsis dassonvillei* subsp. *dassonvillei* DSM 43111	*Nocardiopsis mangrovi* CGMCC 4.7119	84.76	75.11	22.10
567.	*Nocardiopsis ganjiahuensis* DSM 45031	*Nocardiopsis potens* DSM 45234	84.65	75.11	21.4
568.	*Nocardiopsis lucentensis* DSM 44048	*Nocardiopsis xinjiangensis* YIM 90004	84.78	75.08	22
569.	*Nocardiopsis metallicus* JCM 12409	*Nocardiopsis potens* DSM 45234	84.71	75.05	21.6
570.	*Nocardiopsis algeriensis* CECT 8712	*Nocardiopsis halophila* DSM 44494	84.61	75.04	21.2
571.	*Nocardiopsis baichengensis* YIM 90130	*Nocardiopsis sinuspersici* UTMC 00102	84.46	75.04	21.4
572.	*Nocardiopsis dassonvillei* subsp. *crassaminis* D1	*Nocardiopsis gilva* YIM 90087	84.71	75.04	22.60
573.	*Nocardiopsis deserti* H13	*Nocardiopsis mangrovi* CGMCC 4.7119	84.67	75.04	22

574.	*Nocardiopsis potens* DSM 45234	*Nocardiopsis quinghaiensis* YIM 28A4	84.73	75.04	21.6
575.	*Nocardiopsis akebiae* HDS12	*Nocardiopsis mangrovi* CGMCC 4.7119	84.67	75.03	22.10
576.	*Nocardiopsis codii* CT-R113	*Nocardiopsis potens* DSM 45234	84.60	75.03	21.5
577.	*Nocardiopsis exhalans* JCM11759	*Nocardiopsis mwathae* DSM 46659	84.61	75.03	22
578.	*Nocardiopsis alborubida* ATCC 23612	*Nocardiopsis mangrovi* CGMCC 4.7119	84.65	75.02	21.90
579.	*Nocardiopsis flavescens* CGMCC 4.5723	*Nocardiopsis mwathae* DSM 46659	84.66	75.02	21.8
580.	*Nocardiopsis arvandica* DSM 45278	*Nocardiopsis sediminis* TBRC 1826	84.59	75.01	21.7
581.	*Nocardiopsis ganjiahuensis* DSM 45031	*Nocardiopsis mwathae* DSM 46659	84.53	75.01	22
582.	*Nocardiopsis halophila* DSM 44494	*Nocardiopsis quinghaiensis* YIM 28A4	84.51	74.99	21.1
583.	*Nocardiopsis algeriensis* CECT 8712	*Nocardiopsis baichengensis* YIM 90130	84.63	74.98	21.20
584.	*Nocardiopsis baichengensis* YIM 90130	*Nocardiopsis dassonvillei* subsp. *dassonvillei* DSM 43111	84.56	74.97	21.4
585.	*Nocardiopsis coralli* HNM0947	*Nocardiopsis trehalosi* NBRC 14201	84.32	74.97	20.70
586.	*Nocardiopsis ganjiahuensis* DSM 45031	*Nocardiopsis halophila* DSM 44494	84.32	74.97	21
587.	*Nocardiopsis gilva* YIM 90087	*Nocardiopsis sinuspersici* UTMC 00102	84.59	74.97	22.3
588.	*Nocardiopsis mwathae* DSM 46659	*Nocardiopsis quinghaiensis* YIM 28A4	84.62	74.97	21.8
589.	*Nocardiopsis alba* DSM 43377	*Nocardiopsis trehalosi* NBRC 14201	84.60	74.96	21
590.	*Nocardiopsis changdeensis* Mg02	*Nocardiopsis sediminis* TBRC 1826	84.71	74.96	21.5
591.	*Nocardiopsis lucentensis* DSM 44048	*Nocardiopsis mwathae* DSM 46659	84.85	74.96	21.9
592.	*Nocardiopsis codii* CT-R113	*Nocardiopsis mwathae* DSM 46659	84.62	74.95	21.8
593.	*Nocardiopsis codii* CT-R113	*Nocardiopsis halophila* DSM 44494	84.32	74.92	21.2
594.	*Nocardiopsis lambiniae* DSM 44743	*Nocardiopsis mwathae* DSM 46659	84.70	74.92	21.7
595.	*Nocardiopsis mangrovi* CGMCC 4.7119	*Nocardiopsis quinghaiensis* YIM 28A4	84.65	74.92	21.5
596.	*Nocardiopsis dassonvillei* subsp. *dassonvillei* DSM 43111	*Nocardiopsis gilva* YIM 90087	84.78	74.91	22.40
597.	*Nocardiopsis flavescens* CGMCC 4.5723	*Nocardiopsis sediminis* TBRC 1826	84.81	74.91	21.4
598.	*Nocardiopsis aegyptia* DSM 44442	*Nocardiopsis gilva* YIM 90087	84.58	74.89	22.40

599.	*Nocardiopsis exhalans* JCM11759	*Nocardiopsis potens* DSM 45234	84.56	74.89	21.6
600.	*Nocardiopsis arvandica* DSM 45278	*Nocardiopsis gilva* YIM 90087	84.62	74.88	22.2
601.	*Nocardiopsis gilva* YIM 90087	*Nocardiopsis quinghaiensis* YIM 28A4	84.65	74.88	22.2
602.	*Nocardiopsis aegyptia* DSM 44442	*Nocardiopsis sediminis* TBRC 1826	84.54	74.87	21.70
603.	*Nocardiopsis algeriensis* CECT 8712	*Nocardiopsis potens* DSM 45234	84.91	74.87	21.4
604.	*Nocardiopsis quinghaiensis* YIM 28A4	*Nocardiopsis sediminis* TBRC 1826	84.55	74.87	21.7
605.	*Nocardiopsis akebiae* HDS12	*Nocardiopsis gilva* YIM 90087	84.86	74.85	22.20
606.	*Nocardiopsis deserti* H13	*Nocardiopsis gilva* YIM 90087	84.70	74.84	22.4
607.	*Nocardiopsis alborubida* ATCC 23612	*Nocardiopsis gilva* YIM 90087	84.79	74.83	22.40
608.	*Nocardiopsis dassonvillei* subsp. *crassaminis* D1	*Nocardiopsis rhodophaea* JCM 15313	84.82	74.83	22.3
609.	*Nocardiopsis metallicus* JCM 12409	*Nocardiopsis mwathae* DSM 46659	84.51	74.83	21.8
610.	*Nocardiopsis baichengensis* YIM 90130	*Nocardiopsis quinghaiensis* YIM 28A4	84.52	74.81	21.1
611.	*Nocardiopsis lambiniae* DSM 44743	*Nocardiopsis potens* DSM 45234	84.66	74.81	21.3
612.	*Nocardiopsis trehalosi* NBRC 14201	*Nocardiopsis tropica* JCM 10877	84.55	74.81	21.4
613.	*Nocardiopsis algeriensis* CECT 8712	*Nocardiopsis mwathae* DSM 46659	84.78	74.80	21.9
614.	*Nocardiopsis arvandica* DSM 45278	*Nocardiopsis mangrovi* CGMCC 4.7119	84.58	74.79	21.9
615.	*Nocardiopsis changdeensis* Mg02	*Nocardiopsis mangrovi* CGMCC 4.7119	84.72	74.79	21.6
616.	*Nocardiopsis aegyptia* DSM 44442	*Nocardiopsis mangrovi* CGMCC 4.7119	84.52	74.78	21.90
617.	*Nocardiopsis aegyptia* DSM 44442	*Nocardiopsis rhodophaea* JCM 15313	84.6	74.78	22.20
618.	*Nocardiopsis codii* CT-R113	*Nocardiopsis sediminis* TBRC 1826	84.63	74.77	21.8
619.	*Nocardiopsis codii* CT-R113	*Nocardiopsis mangrovi* CGMCC 4.7119	84.58	74.76	21.7
620.	*Nocardiopsis exhalans* JCM11759	*Nocardiopsis halophila* DSM 44494	84.33	74.75	21
621.	*Nocardiopsis listeri* NBRC 13360	*Nocardiopsis trehalosi* NBRC 14201	84.36	74.74	20.6
622.	*Nocardiopsis rhodophaea* JCM 15313	*Nocardiopsis sinuspersici* UTMC 00102	84.61	74.74	22.2
623.	*Nocardiopsis salina* YIM 90010	*Nocardiopsis trehalosi* NBRC 14201	84.34	74.74	20.4

624.	*Nocardiopsis baichengensis* YIM 90130	*Nocardiopsis deserti* H13	84.58	74.73	21.5
625.	*Nocardiopsis baichengensis* YIM 90130	*Nocardiopsis changdeensis* Mg02	84.60	74.71	21.2
626.	*Nocardiopsis baichengensis* YIM 90130	*Nocardiopsis dassonvillei* subsp. *crassaminis* D1	84.60	74.71	21.7
627.	*Nocardiopsis baichengensis* YIM 90130	*Nocardiopsis flavescens* CGMCC 4.5723	84.43	74.69	21
628.	*Nocardiopsis changdeensis* Mg02	*Nocardiopsis gilva* YIM 90087	84.75	74.69	22.2
629.	*Nocardiopsis quinghaiensis* YIM 28A4	*Nocardiopsis rhodophaea* JCM 15313	84.69	74.67	21.9
630.	*Nocardiopsis dassonvillei* subsp. *dassonvillei* DSM 43111	*Nocardiopsis rhodophaea* JCM 15313	84.76	74.66	22.2
631.	*Nocardiopsis halotolerans* DSM 44410	*Nocardiopsis mangrovi* CGMCC 4.7119	84.69	74.64	25.2
632.	*Nocardiopsis akebiae* HDS12	*Nocardiopsis rhodophaea* JCM 15313	84.76	74.63	22
633.	*Nocardiopsis potens* DSM 45234	*Nocardiopsis prasina* DSM 43845	84.61	74.63	21.5
634.	*Nocardiopsis halotolerans* DSM 44410	*Nocardiopsis sediminis* TBRC 1826	84.68	74.62	22.8
635.	*Nocardiopsis arvandica* DSM 45278	*Nocardiopsis rhodophaea* JCM 15313	84.67	74.61	22.1
636.	*Nocardiopsis deserti* H13	*Nocardiopsis rhodophaea* JCM 15313	84.72	74.59	22.1
637.	*Nocardiopsis flavescens* CGMCC 4.5723	*Nocardiopsis mangrovi* CGMCC 4.7119	84.68	74.59	21.3
638.	*Nocardiopsis alborubida* ATCC 23612	*Nocardiopsis rhodophaea* JCM 15313	84.76	74.58	22.1
639.	*Nocardiopsis algeriensis* CECT 8712	*Nocardiopsis sediminis* TBRC 1826	84.74	74.58	21.4
640.	*Nocardiopsis algeriensis* CECT 8712	*Nocardiopsis mangrovi* CGMCC 4.7119	84.76	74.57	21.4
641.	*Nocardiopsis coralli* HNM0947	*Nocardiopsis halophila* DSM 44494	84.27	74.56	20.5
642.	*Nocardiopsis lambiniae* DSM 44743	*Nocardiopsis sediminis* TBRC 1826	84.57	74.56	21.3
643.	*Nocardiopsis codii* CT-R113	*Nocardiopsis gilva* YIM 90087	84.66	74.53	22
644.	*Nocardiopsis mangrovi* CGMCC 4.7119	*Nocardiopsis tropica* JCM 10877	84.71	74.52	21.7
645.	*Nocardiopsis potens* DSM 45234	*Nocardiopsis terrae* KCTC 19431	84.64	74.52	21.5
646.	*Nocardiopsis litoralis* KCTC 19473	*Nocardiopsis trehalosi* NBRC 14201	84.20	74.51	20.7
647.	*Nocardiopsis mwathae* DSM 46659	*Nocardiopsis prasina* DSM 43845	84.78	74.51	21.9
648.	*Nocardiopsis alba* DSM 43377	*Nocardiopsis mwathae* DSM 46659	84.62	74.5	21.80

649.	*Nocardiopsis alba* DSM 43377	*Nocardiopsis potens* DSM 45234	84.81	74.50	21.1
650.	*Nocardiopsis flavescens* CGMCC 4.5723	*Nocardiopsis gilva* YIM 90087	84.64	74.50	21.6
651.	*Nocardiopsis prasina* DSM 43845	*Nocardiopsis sediminis* TBRC 1826	84.60	74.50	21.5
652.	*Nocardiopsis algeriensis* CECT 8712	*Nocardiopsis rhodophaea* JCM 15313	84.74	74.49	22
653.	*Nocardiopsis changdeensis* Mg02	*Nocardiopsis rhodophaea* JCM 15313	84.88	74.49	22
654.	*Nocardiopsis potens* DSM 45234	*Nocardiopsis tropica* JCM 10877	84.69	74.49	21.5
655.	*Nocardiopsis halotolerans* DSM 44410	*Nocardiopsis rhodophaea* JCM 15313	84.71	74.48	32.2
656.	*Nocardiopsis sediminis* TBRC 1826	*Nocardiopsis tropica* JCM 10877	84.62	74.47	21.6
657.	*Nocardiopsis alba* DSM 43377	*Nocardiopsis baichengensis* YIM 90130	84.51	74.45	20.80
658.	*Nocardiopsis algeriensis* CECT 8712	*Nocardiopsis gilva* YIM 90087	84.68	74.45	22.1
659.	*Nocardiopsis ganjiahuensis* DSM 45031	*Nocardiopsis gilva* YIM 90087	84.51	74.45	22.1
660.	*Nocardiopsis ganjiahuensis* DSM 45031	*Nocardiopsis sediminis* TBRC 1826	84.44	74.45	21.6
661.	*Nocardiopsis gilva* YIM 90087	*Nocardiopsis lucentensis* DSM 44048	84.79	74.45	22.3
662.	*Nocardiopsis lucentensis* DSM 44048	*Nocardiopsis quinghaiensis* YIM 28A4	84.81	74.45	22.2
663.	*Nocardiopsis alba* DSM 43377	*Nocardiopsis halophila* DSM 44494	84.45	74.44	20.80
664.	*Nocardiopsis lucentensis* DSM 44048	*Nocardiopsis salina* YIM 90010	84.69	74.44	21.7
665.	*Nocardiopsis mwathae* DSM 46659	*Nocardiopsis terrae* KCTC 19431	84.64	74.44	21.7
666.	*Nocardiopsis exhalans* JCM11759	*Nocardiopsis gilva* YIM 90087	84.55	74.42	22.1
667.	*Nocardiopsis kunsanensis* DSM 44524	*Nocardiopsis trehalosi* NBRC 14201	84.19	74.42	20.6
668.	*Nocardiopsis metallicus* JCM 12409	*Nocardiopsis sediminis* TBRC 1826	84.50	74.42	21.5
669.	*Nocardiopsis coralli* HNM0947	*Nocardiopsis mwathae* DSM 46659	84.48	74.41	21.1
670.	*Nocardiopsis coralli* HNM0947	*Nocardiopsis potens* DSM 45234	84.45	74.4	20.90
671.	*Nocardiopsis exhalans* JCM11759	*Nocardiopsis sediminis* TBRC 1826	84.47	74.40	21.6
672.	*Nocardiopsis lambiniae* DSM 44743	*Nocardiopsis mangrovi* CGMCC 4.7119	84.58	74.39	21.3
673.	*Nocardiopsis ganjiahuensis* DSM 45031	*Nocardiopsis mangrovi* CGMCC 4.7119	84.58	74.38	21.6

674.	*Nocardiopsis gilva* YIM 90087	*Nocardiopsis lambiniae* DSM 44743	84.68	74.38	22
675.	*Nocardiopsis gilva* YIM 90087	*Nocardiopsis prasina* DSM 43845	84.66	74.38	21.9
676.	*Nocardiopsis mangrovi* CGMCC 4.7119	*Nocardiopsis prasina* DSM 43845	84.50	74.38	21.6
677.	*Nocardiopsis halophila* DSM 44494	*Nocardiopsis lucentensis* DSM 44048	84.77	74.37	21.5
678.	*Nocardiopsis listeri* NBRC 13360	*Nocardiopsis mwathae* DSM 46659	84.56	74.37	21.5
679.	*Nocardiopsis lucentensis* DSM 44048	*Nocardiopsis mangrovi* CGMCC 4.7119	84.80	74.37	21.8
680.	*Nocardiopsis alkaliphila* YIM 80379	*Nocardiopsis trehalosi* NBRC 14201	84.24	74.35	20.9
681.	*Nocardiopsis baichengensis* YIM 90130	*Nocardiopsis halotolerans* DSM 44410	84.56	74.35	21.4
682.	*Nocardiopsis codii* CT-R113	*Nocardiopsis rhodophaea* JCM 15313	84.62	74.35	21.9
683.	*Nocardiopsis gilva* YIM 90087	*Nocardiopsis halotolerans* DSM 44410	84.81	74.35	22.3
684.	*Nocardiopsis halophila* DSM 44494	*Nocardiopsis halotolerans* DSM 44410	84.55	74.34	21.4
685.	*Nocardiopsis prasina* DSM 43845	*Nocardiopsis rhodophaea* JCM 15313	84.65	74.34	21.8
686.	*Nocardiopsis baichengensis* YIM 90130	*Nocardiopsis lucentensis* DSM 44048	84.76	74.33	21.5
687.	*Nocardiopsis exhalans* JCM11759	*Nocardiopsis mangrovi* CGMCC 4.7119	84.68	74.32	21.9
688.	*Nocardiopsis ganjiahuensis* DSM 45031	*Nocardiopsis rhodophaea* JCM 15313	84.53	74.32	21.8
689.	*Nocardiopsis metallicus* JCM 12409	*Nocardiopsis rhodophaea* JCM 15313	84.48	74.30	21.8
690.	*Nocardiopsis halophila* DSM 44494	*Nocardiopsis lambiniae* DSM 44743	84.41	74.27	21
691.	*Nocardiopsis baichengensis* YIM 90130	*Nocardiopsis lambiniae* DSM 44743	84.37	74.26	20.9
692.	*Nocardiopsis flavescens* CGMCC 4.5723	*Nocardiopsis rhodophaea* JCM 15313	84.61	74.26	21.6
693.	*Nocardiopsis mwathae* DSM 46659	*Nocardiopsis tropica* JCM 10877	84.73	74.25	21.7
694.	*Nocardiopsis exhalans* JCM11759	*Nocardiopsis rhodophaea* JCM 15313	84.53	74.24	22.1
695.	*Nocardiopsis lambiniae* DSM 44743	*Nocardiopsis rhodophaea* JCM 15313	84.68	74.24	21.6
696.	*Nocardiopsis mangrovi* CGMCC 4.7119	*Nocardiopsis terrae* KCTC 19431	84.51	74.23	21.5
697.	*Nocardiopsis gilva* YIM 90087	*Nocardiopsis terrae* KCTC 19431	84.60	74.21	21.8
698.	*Nocardiopsis listeri* NBRC 13360	*Nocardiopsis potens* DSM 45234	84.48	74.21	21.1

699.	*Nocardiopsis potens* DSM 45234	*Nocardiopsis valliform*is DSM 45023	84.45	74.19	21.6
700.	*Nocardiopsis sediminis* TBRC 1826	*Nocardiopsis terrae* KCTC 19431	84.50	74.17	21.2
701.	*Nocardiopsis baichengensis* YIM 90130	*Nocardiopsis terrae* KCTC 19431	84.40	74.12	21.1
702.	*Nocardiopsis halophila* DSM 44494	*Nocardiopsis terrae* KCTC 19431	84.44	74.12	32
703.	*Nocardiopsis rhodophaea* JCM 15313	*Nocardiopsis terrae* KCTC 19431	84.55	74.10	21.7
704.	*Nocardiopsis halophila* DSM 44494	*Nocardiopsis tropica* JCM 10877	84.39	74.09	21.7
705.	*Nocardiopsis halophila* DSM 44494	*Nocardiopsis prasina* DSM 43845	84.46	74.07	21
706.	*Nocardiopsis baichengensis* YIM 90130	*Nocardiopsis prasina* DSM 43845	84.46	74.03	21
707.	*Nocardiopsis baichengensis* YIM 90130	*Nocardiopsis tropica* JCM 10877	84.33	74.02	21
708.	*Nocardiopsis mwathae* DSM 46659	*Nocardiopsis valliform*is DSM 45023	84.57	74.01	22.1
709.	*Nocardiopsis gilva* YIM 90087	*Nocardiopsis tropica* JCM 10877	84.63	74.00	22
710.	*Nocardiopsis baichengensis* YIM 90130	*Nocardiopsis codii* CT-R113	84.32	73.97	21.1
711.	*Nocardiopsis coralli* HNM0947	*Nocardiopsis gilva* YIM 90087	84.53	73.95	21.5
712.	*Nocardiopsis alba* DSM 43377	*Nocardiopsis gilva* YIM 90087	84.67	73.94	22.00
713.	*Nocardiopsis trehalosi* NBRC 14201	*Nocardiopsis valliform*is DSM 45023	84.35	73.94	21.3
714.	*Nocardiopsis gilva* YIM 90087	*Nocardiopsis metallicus* JCM 12409	84.47	73.91	21.8
715.	*Nocardiopsis alba* DSM 43377	*Nocardiopsis rhodophaea* JCM 15313	84.75	73.90	21.6
716.	*Nocardiopsis coralli* HNM0947	*Nocardiopsis rhodophaea* JCM 15313	84.42	73.9	21.40
717.	*Nocardiopsis rhodophaea* JCM 15313	*Nocardiopsis tropica* JCM 10877	84.74	73.90	21.9
718.	*Nocardiopsis litoralis* KCTC 19473	*Nocardiopsis mwathae* DSM 46659	84.29	73.89	21.1
719.	*Nocardiopsis mangrovi* CGMCC 4.7119	*Nocardiopsis metallicus* JCM 12409	84.43	73.87	21.7
720.	*Nocardiopsis alkaliphila* YIM 80379	*Nocardiopsis baichengensis* YIM 90130	84.32	73.86	20.7
721.	*Nocardiopsis halophila* DSM 44494	*Nocardiopsis metallicus* JCM 12409	84.27	73.86	21
722.	*Nocardiopsis gilva* YIM 90087	*Nocardiopsis valliform*is DSM 45023	84.55	73.85	22
723.	*Nocardiopsis gilva* YIM 90087	*Nocardiopsis listeri* NBRC 13360	84.30	73.84	21.6

724.	*Nocardiopsis kunsanensis* DSM 44524	*Nocardiopsis mwathae* DSM 46659	84.32	73.84	21.1
725.	*Nocardiopsis litoralis* KCTC 19473	*Nocardiopsis potens* DSM 45234	84.32	73.84	21
726.	*Nocardiopsis alba* DSM 43377	*Nocardiopsis sediminis* TBRC 1826	84.52	73.83	20.8
727.	*Nocardiopsis alba* DSM 43377	*Nocardiopsis mangrovi* CGMCC 4.7119	84.61	73.82	21.00
728.	*Nocardiopsis alkaliphila* YIM 80379	*Nocardiopsis mwathae* DSM 46659	84.42	73.82	21.4
729.	*Nocardiopsis listeri* NBRC 13360	*Nocardiopsis rhodophaea* JCM 15313	84.40	73.81	21.4
730.	*Nocardiopsis mangrovi* CGMCC 4.7119	*Nocardiopsis valliformi*s DSM 45023	84.51	73.81	21.7
731.	*Nocardiopsis sediminis* TBRC 1826	*Nocardiopsis valliformi*s DSM 45023	84.36	73.81	21.3
732.	*Nocardiopsis kunsanensis* DSM 44524	*Nocardiopsis potens* DSM 45234	84.33	73.79	21
733.	*Nocardiopsis coralli* HNM0947	*Nocardiopsis sediminis* TBRC 1826	84.3	73.78	20.80
734.	*Nocardiopsis baichengensis* YIM 90130	*Nocardiopsis metallicus* JCM 12409	84.20	73.75	20.9
735.	*Nocardiopsis listeri* NBRC 13360	*Nocardiopsis sediminis* TBRC 1826	84.24	73.75	20.9
736.	*Nocardiopsis baichengensis* YIM 90130	*Nocardiopsis ganjiahuensis* DSM 45031	84.34	73.72	20.9
737.	*Nocardiopsis baichengensis* YIM 90130	*Nocardiopsis litoralis* KCTC 19473	84.20	73.72	20.9
738.	*Nocardiopsis alkaliphila* YIM 80379	*Nocardiopsis halophila* DSM 44494	84.35	73.71	20.8
739.	*Nocardiopsis alkaliphila* YIM 80379	*Nocardiopsis potens* DSM 45234	84.42	73.70	21
740.	*Nocardiopsis coralli* HNM0947	*Nocardiopsis mangrovi* CGMCC 4.7119	84.42	73.70	21
741.	*Nocardiopsis salina* YIM 90010	*Nocardiopsis sediminis* TBRC 1826	84.30	73.70	20.5
742.	*Nocardiopsis baichengensis* YIM 90130	*Nocardiopsis exhalans* JCM11759	84.30	73.67	21
743.	*Nocardiopsis baichengensis* YIM 90130	*Nocardiopsis xinjiangensis* YIM 90004	84.19	73.67	20.5
744.	*Nocardiopsis listeri* NBRC 13360	*Nocardiopsis mangrovi* CGMCC 4.7119	84.23	73.67	21.1
745.	*Nocardiopsis mwathae* DSM 46659	*Nocardiopsis xinjiangensis* YIM 90004	84.27	73.67	20.8
746.	*Nocardiopsis rhodophaea* JCM 15313	*Nocardiopsis valliformi*s DSM 45023	84.53	73.67	22.1
747.	*Nocardiopsis gilva* YIM 90087	*Nocardiopsis litoralis* KCTC 19473	84.20	73.65	21.2
748.	*Nocardiopsis potens* DSM 45234	*Nocardiopsis xinjiangensis* YIM 90004	84.29	73.65	20.8

749.	*Nocardiopsis trehalosi* NBRC 14201	*Nocardiopsis xinjiangensis* YIM 90004	84.23	73.65	20.6
750.	*Nocardiopsis halophila* DSM 44494	*Nocardiopsis litoralis* KCTC 19473	84.23	73.61	20.7
751.	*Nocardiopsis halophila* DSM 44494	*Nocardiopsis valliformi*s DSM 45023	84.23	73.61	21
752.	*Nocardiopsis baichengensis* YIM 90130	*Nocardiopsis coralli* HNM0947	84.25	73.60	20.6
753.	*Nocardiopsis baichengensis* YIM 90130	*Nocardiopsis valliformi*s DSM 45023	84.24	73.57	20.7
754.	*Nocardiopsis halophila* DSM 44494	*Nocardiopsis xinjiangensis* YIM 90004	84.09	73.54	20.7
755.	*Nocardiopsis gilva* YIM 90087	*Nocardiopsis xinjiangensis* YIM 90004	84.27	73.52	21.2
756.	*Nocardiopsis litoralis* KCTC 19473	*Nocardiopsis mangrovi* CGMCC 4.7119	84.20	73.52	21.1
757.	*Nocardiopsis mwathae* DSM 46659	*Nocardiopsis salina* YIM 90010	84.32	73.52	21.1
758.	*Nocardiopsis gilva* YIM 90087	*Nocardiopsis salina* YIM 90010	84.27	73.51	21.3
759.	*Nocardiopsis baichengensis* YIM 90130	*Nocardiopsis listeri* NBRC 13360	84.28	73.48	20.6
760.	*Nocardiopsis halophila* DSM 44494	*Nocardiopsis kunsanensis* DSM 44524	84.24	73.47	20.6
761.	*Nocardiopsis litoralis* KCTC 19473	*Nocardiopsis sediminis* TBRC 1826	84.16	73.47	20.6
762.	*Nocardiopsis mangrovi* CGMCC 4.7119	*Nocardiopsis xinjiangensis* YIM 90004	84.09	73.47	20.6
763.	*Nocardiopsis alkaliphila* YIM 80379	*Nocardiopsis sediminis* TBRC 1826	84.13	73.46	20.9
764.	*Nocardiopsis potens* DSM 45234	*Nocardiopsis salina* YIM 90010	84.48	73.46	20.9
765.	*Nocardiopsis alkaliphila* YIM 80379	*Nocardiopsis gilva* YIM 90087	84.31	73.45	21.7
766.	*Nocardiopsis baichengensis* YIM 90130	*Nocardiopsis kunsanensis* DSM 44524	84.24	73.45	20.8
767.	*Nocardiopsis kunsanensis* DSM 44524	*Nocardiopsis mangrovi* CGMCC 4.7119	84.20	73.45	21.1
768.	*Nocardiopsis gilva* YIM 90087	*Nocardiopsis kunsanensis* DSM 44524	84.23	73.44	21.2
769.	*Nocardiopsis kunsanensis* DSM 44524	*Nocardiopsis rhodophaea* JCM 15313	84.26	73.44	21.2
770.	*Nocardiopsis halophila* DSM 44494	*Nocardiopsis listeri* NBRC 13360	84.30	73.42	20.5
771.	*Nocardiopsis kunsanensis* DSM 44524	*Nocardiopsis sediminis* TBRC 1826	84.18	73.41	20.6
772.	*Nocardiopsis sediminis* TBRC 1826	*Nocardiopsis xinjiangensis* YIM 90004	84.11	73.41	20.7
773.	*Nocardiopsis alkaliphila* YIM 80379	*Nocardiopsis mangrovi* CGMCC 4.7119	84.19	73.39	21.1

774.	*Nocardiopsis litoralis* KCTC 19473	*Nocardiopsis rhodophaea* JCM 15313	84.26	73.39	21.3
775.	*Nocardiopsis mangrovi* CGMCC 4.7119	*Nocardiopsis salina* YIM 90010	84.36	73.38	20.8
776.	*Nocardiopsis baichengensis* YIM 90130	*Nocardiopsis salina* YIM 90010	84.28	73.37	20.7
777.	*Nocardiopsis halophila* DSM 44494	*Nocardiopsis salina* YIM 90010	84.26	73.36	20.7
778.	*Nocardiopsis alkaliphila* YIM 80379	*Nocardiopsis rhodophaea* JCM 15313	84.32	73.35	21.2
779.	*Nocardiopsis rhodophaea* JCM 15313	*Nocardiopsis xinjiangensis* YIM 90004	84.19	73.35	21.0
780	*Nocardiopsis rhodophaea* JCM 15313	*Nocardiopsis salina* YIM 90010	84.30	73.25	21.2

Note: Completeness >50%, Contamination<10%.

Table S7. Estimated ANIm and ANIb species delineation thresholds for the genus *Nocardiopsis* based on different genome quality criteria

Genome Quality Criteria	No. of Strains	No. of Pairs	Estimated ANIm Threshold (%)	Estimated ANIb Threshold (%)
>90% completeness, <5% contamination	13	78	96.68	96.15
>90% completeness, <10% contamination	34	561	96.70	96.02
>50% completeness, <10% contamination	40	780	96.69	96.06

Table S8. Cultural characteristics of HUAS JQ3^{T} and *N. akebiae* HDS 12^{T}.

Characteristics	1	2
Color of aerial mycelium on R2A	Pale olive buff	Pale olive buff
Color of substrate mycelium on R2A	Citrine drab	Fawn color
Diffusible pigment on R2A	None	None
Color of aerial mycelium on No.1	Ivory yellow	White
Color of substrate mycelium on No.1	Dark greenish olive	Lasbella color
Diffusible pigment on No.1	None	None
Color of aerial mycelium on ISP 2	White and eoru-drab	White
Color of substrate mycelium on ISP 2	Benzo brown	Orange citrine
Diffusible pigment on ISP 2	Light drab	None
Color of aerial mycelium on ISP 3	Smoke gray and blue	White
Color of substrate mycelium on ISP 3	Light grayish olive	Martius yellow
Diffusible pigment on ISP 3	None	None
Color of aerial mycelium on ISP 4	Pale olive-buff	None
Color of substrate mycelium on ISP 4	Deep olive-buff	None
Diffusible pigment on ISP 4	Deep olive-buff	None
Color of aerial mycelium on ISP 5	Gray to White	White
Color of substrate mycelium on ISP 5	Citrine-drab	White
Diffusible pigment on ISP 5	None	None
Color of aerial mycelium on ISP 6	White	Sulphur yellow
Color of substrate mycelium on ISP 6	Olive Lake	Citrine
Diffusible pigment on ISP 6	None	None
Color of aerial mycelium on ISP 7	Jouvence blue and White	Sulphur yellow
Color of substrate mycelium on ISP 7	Jouvence blue	Citrine
Diffusible pigment on ISP 7	None	None

Note: 1, HUAS JQ3^{T}; 2, *N. akebiae* HDS 12^{T}. R2A, Reasoner' 2A; No.1, Gause's synthetic No.1 medium. All data were from this study.

Table S9. The fatty acid composition of strains HUAS JQ3^T and *N. akebiae* HDS 12^T.

Fatty acid (%)	HUAS JQ3^T	[a]HDS 12^T
$C_{9:0}$ 3 OH	0.6	–
$C_{10:0}$ 3OH	–	tr
$C_{12:0}$	tr	–
anteiso-$C_{13:0}$	0.4	–
iso-$C_{14:0}$	0.5	1.0
$C_{14:0}$	0.6	0.6
iso-$C_{15:0}$	tr	0.6
anteiso-$C_{15:0}$	0.6	1.3
$C_{15.1}$ ω8c	–	tr
iso-$C_{16:0}$	33.6	43.2
$C_{16:0}$	4.0	2.5
iso-$C_{17:0}$	2.0	1.3
anteiso-$C_{17:0}$	9.4	6.0
$C_{17:1}$ ω8c	2.6	7.3
$C_{17:0}$	0.7	1.1
$C_{17:0}$ 10-methyl	3.7	1.7
iso-$C_{18:0}$	4.3	3.0
$C_{18:1}$ ω9c	17.4	19.0
$C_{18:0}$	2.1	3.7
iso-$C_{17:0}$ 3 OH	–	0.6
$C_{18:0}$ 10-methyl (TBSA)	10.9	1.2
iso-$C_{19:0}$	–	tr
anteiso-$C_{20:0}$	–	tr
$C_{20:0}$	–	tr
anteiso-$C_{19:0}$	0.5	–
Summed Feature 3	1.4	2.6
Summed Feature 5	2.4	tr
Summed Feature 8	0.5	–
Summed Feature 9	0.8	–

Note: [a]Data are from Mo et al. (2022) [46]. tr, trace amount (<0.5%); –, not detected. Summed Feature 3 contained $C_{16:1}$ ω7c/$C_{16:1}$ ω6c; Summed Feature 5 C18:2 ω6,9c/ $C_{18:0}$ ante; Summed Feature 8 contained $C_{17:1}$ ω7c/$C_{18:1}$ ω6c; Summed Feature 9 contained *iso*-$C_{17:1}$ ω9c and/or $C_{16:0}$ 10-methyl.

Table S10. Genome features of strains HUAS JQ3^T and *N. akebiae* HDS 12^T.

Strains	1	2
Size (bp)	6,813,696	6,602,217
Genes (total)	5,953	5,671
CDSs (total)	5,875	5,595
Genes (coding)	5,820	5,454
CDSs (with protein)	5,820	5,454
Genes (RNA)	78	76
rRNAs	15	15
complete rRNAs	15	15
tRNAs	60	58
ncRNAs	3	3
Pseudo Genes (total)	55	141
CDSs (without protein)	55	141
Pseudo Genes (ambiguous residues)	0	0
Pseudo Genes (frameshifted)	16	66
Pseudo Genes (incomplete)	45	85
Pseudo Genes (internal stop)	3	12
Pseudo Genes (multiple problems)	9	22
DNA G+C content	72.4	72.4
Accession numbers	CP118614	CP074132

Note: 1, HUAS JQ3^T; 2, *N. akebiae* HDS 12^T.

Table S11. The subsystem category number of genes of strains HUAS JQ3^T and *N. akebiae* HDS 12^T based on RAST annotation server.

Subsystem Feature Counts	1	2
Carbohydrates	294	301
Amino Acids and Derivatives	266	275
Protein Metabolism	196	199
Cofactors, Vitamins, Prosthetic Groups, Pigments	165	164
Fatty Acids, Lipids, and Isoprenoids	94	101
Nucleosides and Nucleotides	91	94
DNA Metabolism	85	72
Respiration	77	77
Virulence, Disease and Defense	48	39
Membrane Transport	47	47
Stress Response	47	47
RNA Metabolism	48	48
Regulation and Cell signaling	27	22
Iron acquisition and metabolism	26	26
Nitrogen Metabolism	25	25
Cell Wall and Capsule	24	24
Miscellaneous	24	27
Phosphorus Metabolism	22	22
Metabolism of Aromatic Compounds	21	20
Secondary Metabolism	9	6
Sulfur Metabolism	6	7
Potassium metabolism	5	2
Dormancy and Sporulation	5	5

Note: 1, HUAS JQ3^T; 2, *N. akebiae* HDS 12^T.

Table S12. The distribution of biosynthetic gene clusters in the genome of strains HUAS JQ3^T and *N. akebiae* HDS 12^T by antiSMASH analyses.

	1	2
NRPS	4	3
Terpene-precursor	3	3
RiPP-like	2	2
T1PKS	2	2
Terpene	2	2
CDPS	1	1
Deazapurine	1	0
Ectoine	1	1
Lanthipeptide-class-i	1	1
Lanthipeptide-class-iii	1	1
Lassopeptide	1	0
NI-siderophore	1	1
NRP-metallophore	1	1
Oligosaccharide	1	1
Phenazine	1	1
Phosphonate	1	0
PKS-like	1	2
T2PKS	1	1
Triceptide	1	0
Butyrolactone	0	1
Deazapurine	0	1
TransAT-PKS	0	1
Other	0	1

Note: 1, HUAS JQ3^T; 2, *N. akebiae* HDS 12^T. NRPS, Non-ribosomal peptide synthetase; terpene-precursor, Compound likely used as a terpene precursor; RiPP-like, Other unspecified ribosomally synthesised and post-translationally modified peptide product (RiPP); T1PKS, Type I PKS (Polyketide synthase); CDPS, tRNA-dependent cyclodipeptide synthases; Lanthipeptide-class-i, Class I lanthipeptides like nisin; lanthipeptide class III , Class III lanthipeptides like labyrinthopeptin (FN178622); NI-siderophore, NRPS-independent, IucA/IucC-like siderophores (siderophore prior to 7.0); NRP-metallophore, Non-ribosomal peptide

metallophores; PKS-like, Other types of PKS; transAT-PKS-like, Trans-AT PKS fragment, with trans-AT domain not found; other, Cluster containing a secondary metabolite-related protein that does not fit into any other category.
